# Increased overall cortical connectivity with syndrome specific local decreases suggested by atypical sleep-EEG synchronization in Williams syndrome

**DOI:** 10.1038/s41598-017-06280-2

**Published:** 2017-07-21

**Authors:** Ferenc Gombos, Róbert Bódizs, Ilona Kovács

**Affiliations:** 1 Department of General Psychology, Institute of Psychology, Pázmány Péter Catholic University, 1088 Budapest, Hungary; 2 Institute of Behavioural Sciences, Semmelweis University, 1089 Budapest, Hungary

## Abstract

Williams syndrome (7q11.23 microdeletion) is characterized by specific alterations in neurocognitive architecture and functioning, as well as disordered sleep. Here we analyze the region, sleep state and frequency-specific EEG synchronization of whole night sleep recordings of 21 Williams syndrome and 21 typically developing age- and gender-matched subjects by calculating weighted phase lag indexes. We found broadband increases in inter- and intrahemispheric neural connectivity for both NREM and REM sleep EEG of Williams syndrome subjects. These effects consisted of increased theta, high sigma, and beta/low gamma synchronization, whereas alpha synchronization was characterized by a peculiar Williams syndrome-specific decrease during NREM states (intra- and interhemispheric centro-temporal) and REM phases of sleep (occipital intra-area synchronization). We also found a decrease in short range, occipital connectivity of NREM sleep EEG theta activity. The striking increased overall synchronization of sleep EEG in Williams syndrome subjects is consistent with the recently reported increase in synaptic and dendritic density in stem-cell based Williams syndrome models, whereas decreased alpha and occipital connectivity might reflect and underpin the altered microarchitecture of primary visual cortex and disordered visuospatial functioning of Williams syndrome subjects.

## Introduction

Neurodevelopmental disorders are caused by significant and persistent disruption of the dynamic inter-relationship between genetic, brain, cognitive, emotional, and behavioural processes across the developmental lifespan^1^. Due to the high economic burden caused by these conditions, it is of utmost importance to reveal new technologies providing sensitive biomarkers that can be related to the brain and behavioural developmental integrity^1^.

Heritable and de novo genomic lesions are among the six causes of neurodisability in children^1^. The hemideletion of 25 to 28 genes at 7q11.23 causes a neurodevelopmental disorder known as Williams syndrome (or Williams-Beuren syndrome). The disorder occurs in 1 of 20 000 live birthsand is characterized by mild to moderate intellectual disability, learning difficulties, cardiovascular abnormalities, high sociability and empathy and a distinctive cognitive-linguistic profile^2, 3^. Attention deficit/hyperactivity disorder, specific phobias and generalized anxiety disorder are among the particularly frequent comorbid psychiatric syndromes of subjects with Williams syndrome^2^.

Subjects with neurodevelopmental disabilities are often affected by sleep disorders^4^. Williams syndrome has been characterized by altered sleep in several respects. Difficulties in initiating and maintaining sleep, decreases in total sleep, rapid eye movement (REM) sleep and sleep efficiency, as well as increases in intra-sleep wakefulness, slow wave sleep and daytime sleepiness are among the common findings^5–11^. We have shown that sleep cycles are fragmented and the cyclicity of sleep is disorganized^8^. In addition to that, we have also shown increases in non- rapid eye movement (NREM) sleep frontal EEG delta activity, region-independent decreases in alpha and sigma waves, as well as accelerations of sigma peak frequencies^8, 10, 12^. These sleep macrostructural and EEG alterations are present both in children and young adults with Williams syndrome.

Functional connectivity is a state and trait-dependent feature of neural networks. Sleep is a period of spontaneous synchronization of the neural architecture. Recent findings suggest that NREM sleep EEG coherence reflects the programmed unfolding of neuronal networks during ontogenetic development in children^13^. Indeed, plastic changes in sleep EEG connectivity have currently been associated with the development of complex cognitive functions^14^. Thus, the peculiarities of network synchronization are major candidates for unravelling neural signatures of developmental and neurobehavioural disabilities^15, 16^. Studies suggest that specific patterns of task-related synchronization, resting-state-related synchronization and sleep-state-specific synchronization patterns are characteristic features of different neuropsychiatric and neurodevelopmental conditions^17, 18^. The specific sleep-EEG alterations of Williams syndrome subjects revealed by our team so far^8, 10, 12, 19^ lead us to focus on the synchronization properties of NREM and REM sleep EEG of subjects with Williams syndrome. Recent resting state fMRI investigations of Williams syndrome populations revealed a peculiar pattern of Default Mode Network (DMN) underconnectivity together with a concomitant global interhemispheric and between network overconnectivity^20, 21^. Furthermore, altered network connectivity, longer total dendrites, as well as an increased number of spines and synapses are characteristic features of a Williams syndrome model based on human induced pluripotent stem cells^22^. In addition to that, both micro- and macro-anatomical alterations of the primary visual cortices [occipital lobes] were reported as peculiar features of Williams syndrome: increases in neuronal-packing density and decreases in cell sizes^23^ were paralleled by volume losses of gray matter structures^24^ and decreased performances in basic visual tasks^25^. Evidence suggests the correlation between structural and functional connectivity of neuronal structures^26, 27^. Despite all these convergent findings, sleep-state-dependent neural synchronization/ connectivity has not been tested in Williams syndrome before.

Based on the above findings, we aim to characterize the peculiarities of functional neural connectivity during sleep in subjects with Williams syndrome. Home recorded sleep EEG of Williams syndrome and typically developing subjects was analyzed by the Weighted Phase Lag Index (WPLI) method^28^ and averaged over standard frequency bands [in addition to the non-standard broadband-1 (0.5–30 Hz) and broadband-2 (0.5–100 Hz) frequencies], sleep states (NREM and REM), as well as specific, pre-defined inter- and intra-regional cortical pairings (for details see Methods). On the basis of the cited literature, we hypothesize a significant broadband overconnectivity of neural oscillations during sleep in subjects with Williams syndrome (H1). Moreover, patterns of underconnectivity, specific for the DMN inter-area synchronizations and local occipito-occipital (O-O) relationships are also expected (H2).

## Results

Statistical analyses aiming to unravel the Williams syndrome vs. typically developing group differences were performed in a stepwise manner starting from the tests of regional unspecific and broadband effects, gradually attaining region- and frequency-specific peculiarities in the synchronization of sleep EEG in Williams syndrome.

### NREM sleep

#### Spatially unspecific broadband connectivity

Higher overall connectivity in Williams syndrome (F = 9.36; d.f. = 1, 38; p = 0.0040), higher broadband-1 vs broadband-2 connectivity (F = 184.11; d.f. = 1, 38; p < 0.0001), as well as an unequal broadband-1 vs broadband-2 difference between the groups (F = 4.91; d.f. = 1, 38; p = 0.0327) were revealed by a 2-way ANOVA [Group × Band (broadband-1 vs broadband-2)]. Post-hoc tests (Fisher Least Square Differences) indicated a significantly increased NREM sleep EEG broadband-1 WPLI mean in Williams syndrome as compared to the typically developing group (p = 0.0003), and the lack of a significant group difference in the broadband-2 range (p = 0.1651) (see Supplementary Fig. S1).

### Broadband intra- and interhemispheric connectivity

**Figure 1:**
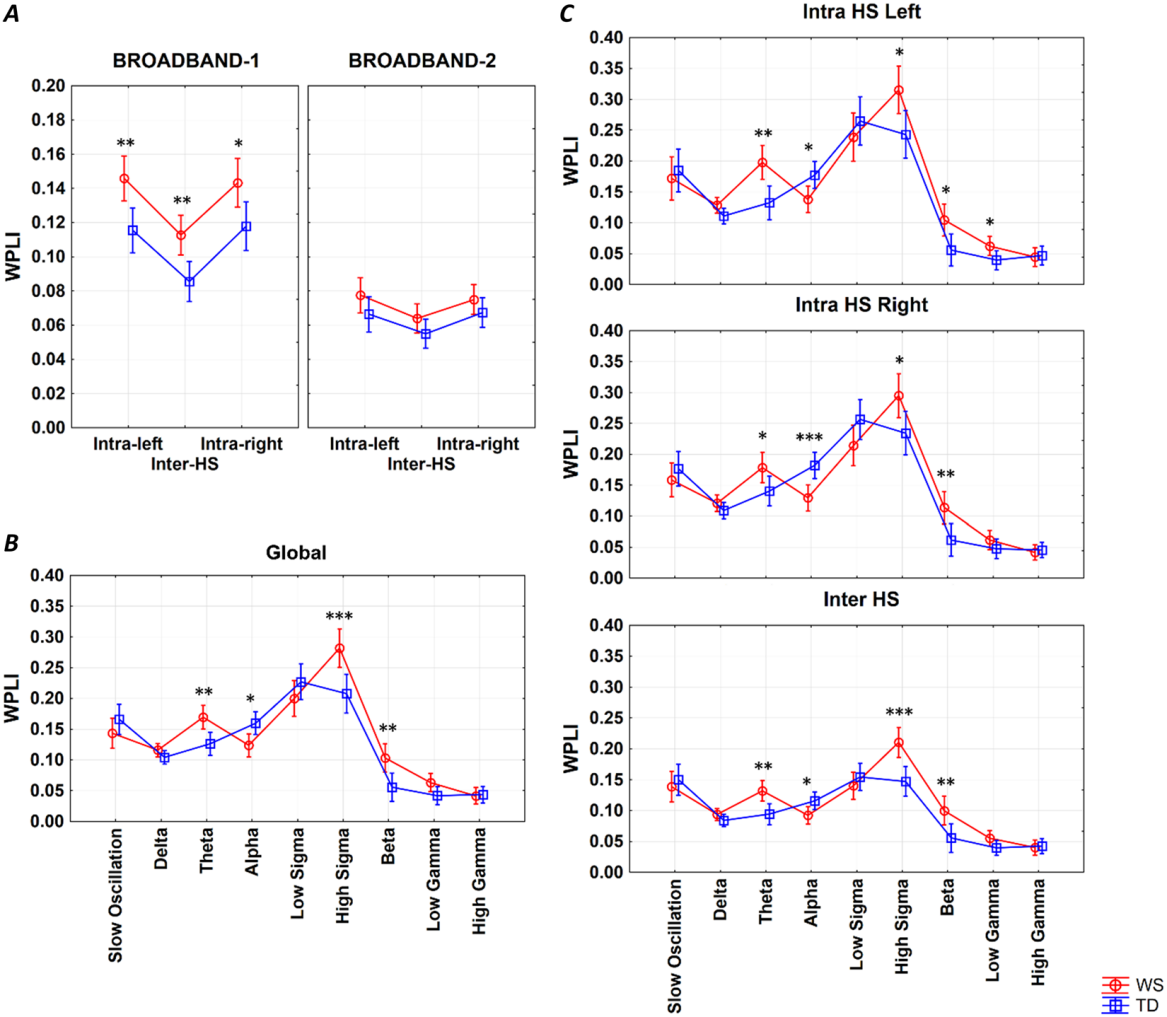
NREM sleep EEG WPLI means. (**A**) Intra- and interhemispheric NREM sleep EEG broadband-1 and broadband-2 WPLI means of Williams syndrome and typically developing subjects (means and 95% confidence intervals). Intra-left – left intrahemispheric, Inter-HS – interhemispheric, Intra-right – right intrahemispheric. *p < 0.05, **p < 0.01, according to post-hoc Fisher LSD tests following a 3-way ANOVA with repeated measures (Group × Intrahemispheric/Interhemispheric × Band). (**B**) Band-limited global WPLI means of Williams syndrome and typically developing subjects’ NREM sleep EEG recordings (means and 95% confidence intervals). *p < 0.05, **p < 0.01, ***p < 0.001, according to post-hoc Fisher LSD tests following a 2-way ANOVA with repeated measures (Group × Band). (**C**) Band-limited intra- and interhemispheric WPLI means of Williams syndrome and typically developing subjects’ NREM sleep EEG recordings (means and 95% confidence intervals). *p < 0.05, **p < 0.01, *** p < 0.001, according to one-way ANOVAs.

We observed overall enhanced broadband connectivity of Williams syndrome subjects (main effect: F = 8.66; d.f. = 1, 38; p < 0.0054). This resulted from higher broadband-1 WPLI of Williams syndrome subjects as compared to typically developing participants (Group × Band: F = 5.44; d.f. = 1, 38; p = 0.0250). Both intrahemispheric (left and right) and interhemispheric broadband-1 connectivity was significantly higher in Williams syndrome as compared to typically developing subjects (results supported by post-hoc tests, see Fig. 1A and Supplementary Table S1).

Increased intrahemispheric as compared to interhemispheric connectivity (F = 71.73; d.f. = 2, 76; p < 0.0001), increased broadband-1 as compared to broadband-2 WPLI (F = 178.25; d.f. = 1, 38; p < 0.0001), as well as unequal Intra/inter hemispheric connection differences in the two bands (F = 45.68; d.f. = 2, 76; p < 0.0001) were seen in both groups (Williams syndrome, typically developing).

### Band-limited, spatially unspecific connectivity

The increased overall connectivity of Williams syndrome subjects (F = 5.59; d.f. = 1, 38; p = 0.0232) was unevenly distributed among the different frequency bands (Group × Band: F = 6.35; d.f. = 8, 304; p < 0.0001), as follows. Enhanced WPLI values for theta (4.75–7.25 Hz), high sigma (13–15 Hz) and beta (15.25–30 Hz) activities were found in Williams syndrome subjects (post-hoc tests, see Fig. 1B). In turn, the synchronization of the NREM sleep EEG alpha (7.5–10.75 Hz) frequency activities of Williams syndrome subjects was characterized by a striking decrease (Fig. 1B and Supplementary Table S2). It has to be mentioned, that different frequency bands were characterized by different levels of connectivity during NREM sleep in both groups (main effect of band: F = 84.08; d.f. = 8, 304; p < 0.0001).

### Band-limited intra- and interhemispheric connectivity

Williams syndrome as compared to typically developing subjects were characterized by significantly higher theta, high sigma and beta connectivity in both left and right intrahemispheric, as well as interhemipsheric electrode pairings. In turn alpha WPLI values were decreased in Williams syndrome subjects independent of intra- or interhemispheric focus Furthermore, left intrahemispheric low gamma WPLI was increased in Williams syndrome as compared to the typically developing subjects (Fig. 1C and Supplementary Table S3).

### Region-specific broadband connectivity

**Figure 2:**
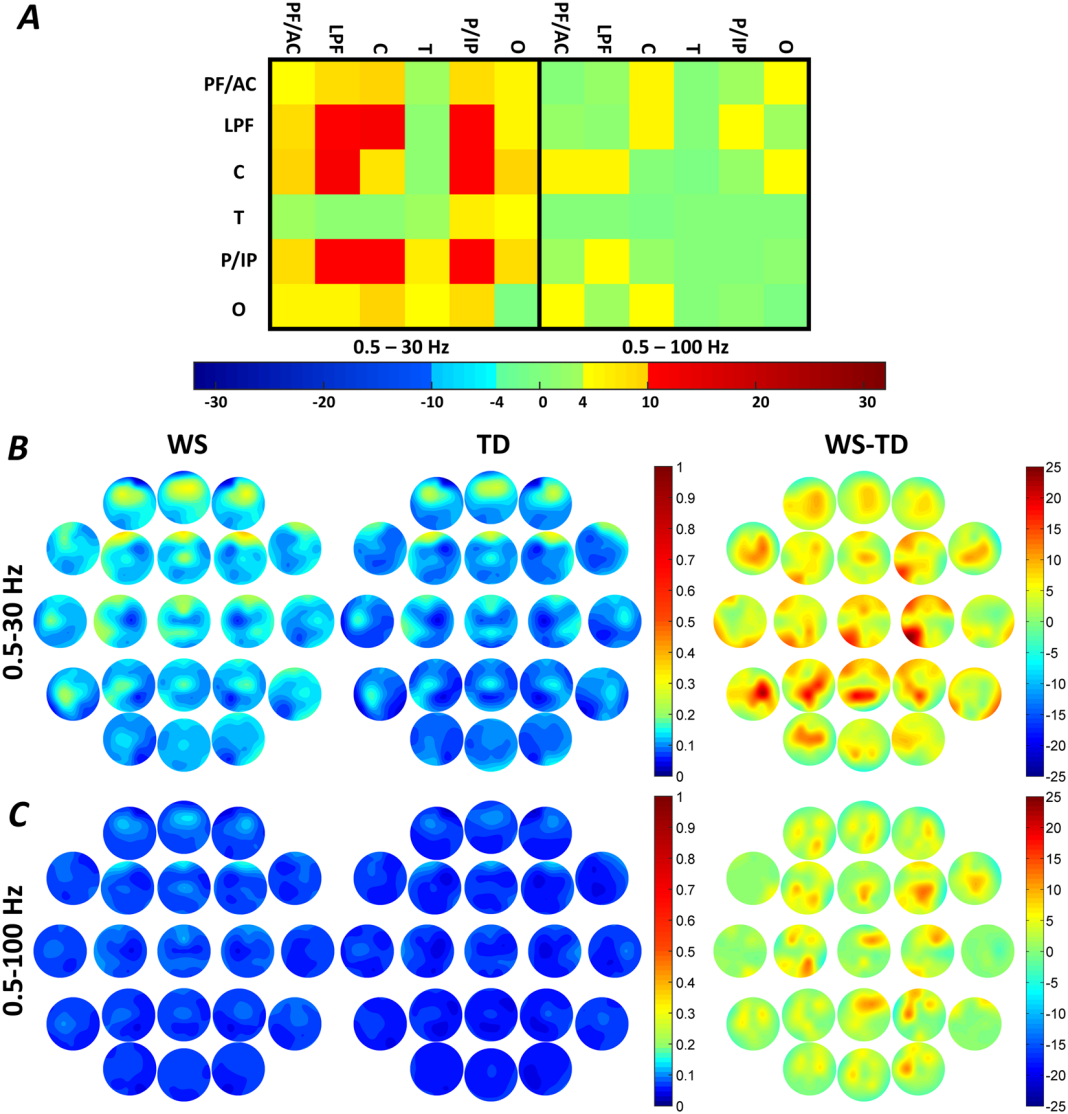
Region-specific NREM sleep EEG broadband-1 (0.5–30 Hz) and broadband-2 (0.5–100 Hz) WPLI differences. (**A**) Broadband-1 WPLIs are significantly higher in Williams syndrome as compared to the typically developing group in several intra- and inter-regional pairings. Color codes: Red = Williams syndrome > typically developing (B-H corrected), Yellow-orange = Williams syndrome > typically developing (uncorrected), Green = Williams syndrome ≈ typically developing, Light blue = Williams syndrome < typically developing (uncorrected), Blue = Williams syndrome < typically developing (B-H corrected). (**B**,**C**) Broadband-1 and broadband-2 WPLI maps highlighting the patterns of absolute group means (left: Williams syndrome; middle: typically developing) and Williams syndrome-typically developing differences (right). Positions of the maps represent the seed derivation of the synchronization analyses (according to the 10–20 system), while the color patterns are the representations of the variable strengths in the synchronization (WPLI) between the respective region and the seed derivation.

Williams syndrome subjects were characterized by significant increases in intra-regional lateral prefrontal (LPF) and parietal-inferior parietal (P/IP) broadband-1 WPLI means. In addition, increased inter-regional LPF-central (C), LPF-P/IP and P/IP-C broadband-1 WPLI means of the Williams syndrome group were also unraveled (Fig. 2 and Supplementary Tables S4, S5 and S6). No significant intra- and inter-regional broadband-2 WPLI differences were found in NREM sleep.

### Region specific, band-limited connectivity

#### WPLI decreases of Williams syndrome subjects

**Figure 3:**
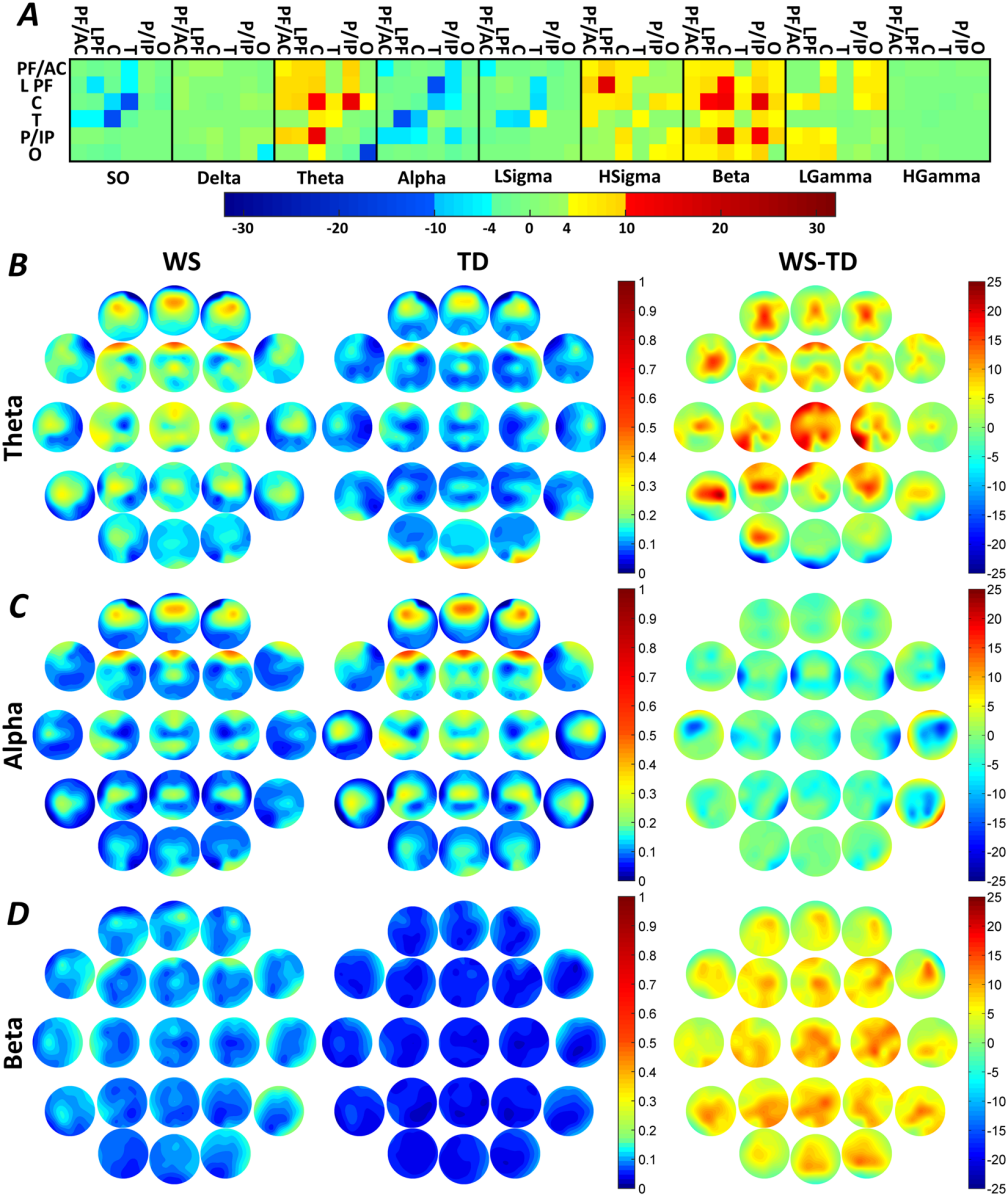
Region-specific and band-limited NREM sleep EEG connectivity differences. Decreased WPLI was evidenced in the slow oscillation range for the C-T inter-regional pairing, as well as in the intra-regional O theta and LPF-T alpha synchronizations. Several Williams syndrome-specific increases in WPLIs were found in the theta, high sigma and beta ranges. Frequency range codes: SO = Slow oscillation, LSigma = Low sigma, HSigma = High sigma, LGamma = Low gamma, HGamma = High gamma. Color codes: Red = Williams syndrome > typically developing (B-H corrected), Yellow-orange = Williams syndrome > typically developing (uncorrected), Green = Williams syndrome ≈ typically developing, Light blue = Williams syndrome < typically developing (uncorrected), Blue = Williams syndrome < typically developing (B-H corrected). B-D. Theta, alpha and beta WPLI maps highlighting the patterns of absolute group means (left: Williams syndrome; middle: typically developing) and Williams syndrome-typically developing differences (right). Positions of the maps represent the seed derivation of the synchronization analyses (according to the 10–20 system), while the color patterns are the representations of the variable strengths in the synchronization (WPLI) between the respective region and the seed derivation.

Williams syndrome subjects were characterized by decreased inter-regional WPLI quantifying the slow oscillation (0.5–1 Hz)-specific connectivity between the C and the temporal (T) regions. Likewise, decreases in NREM-sleep-dependent LPF-T connectivity of Williams syndrome subjects was evidenced for the alpha band. In addition, occipital (O) intra-regional WPLI decreases in the theta band were characteristic of the Williams syndrome group (Fig. 3 and Supplementary Tables S7, S8, S9 and S10).

#### WPLI increases of Williams syndrome subjects

Increases in C, intra-regional and C - P/IP inter-regional theta WPLI values, LPF, intra-regional high sigma values were characteristic features of the Williams syndrome group. Several intra- and inter-regional Williams syndrome-specific WPLI increases were found in the beta frequency band as follows: intra-regional C and P/IP, as well as inter-regional LPF-C and C-P/IP WPLIs were found in Williams syndrome (Fig. 3 and Supplementary Tables S7, S8, S9 and S10).

### REM sleep

#### Spatially unspecific broadband connectivity

Higher overall connectivity in Williams syndrome (F = 21.80; d.f. = 1, 38; p < 0.0001), higher broadband-1 vs broadband-2 connectivity (F = 42.72; d.f. = 1, 38; p < 0.0001), as well as an unequal broadband-1 vs broadband-2 difference between the groups (F = 6.96; d.f. = 1, 38; p = 0.0120) were revealed by a 2-way ANOVA [Group × Band (broadband-1 vs broadband-2)]. Post-hoc Fisher LSD indicated a significantly increased REM sleep EEG broadband-1 and broadband-2 WPLI mean in Williams syndrome as compared to the typically developing group (p < 0.0001 and p = 0.0339, respectively) (see Supplementary Fig. S2).

### Broadband intra- and interhemispheric connectivity

**Figure 4:**
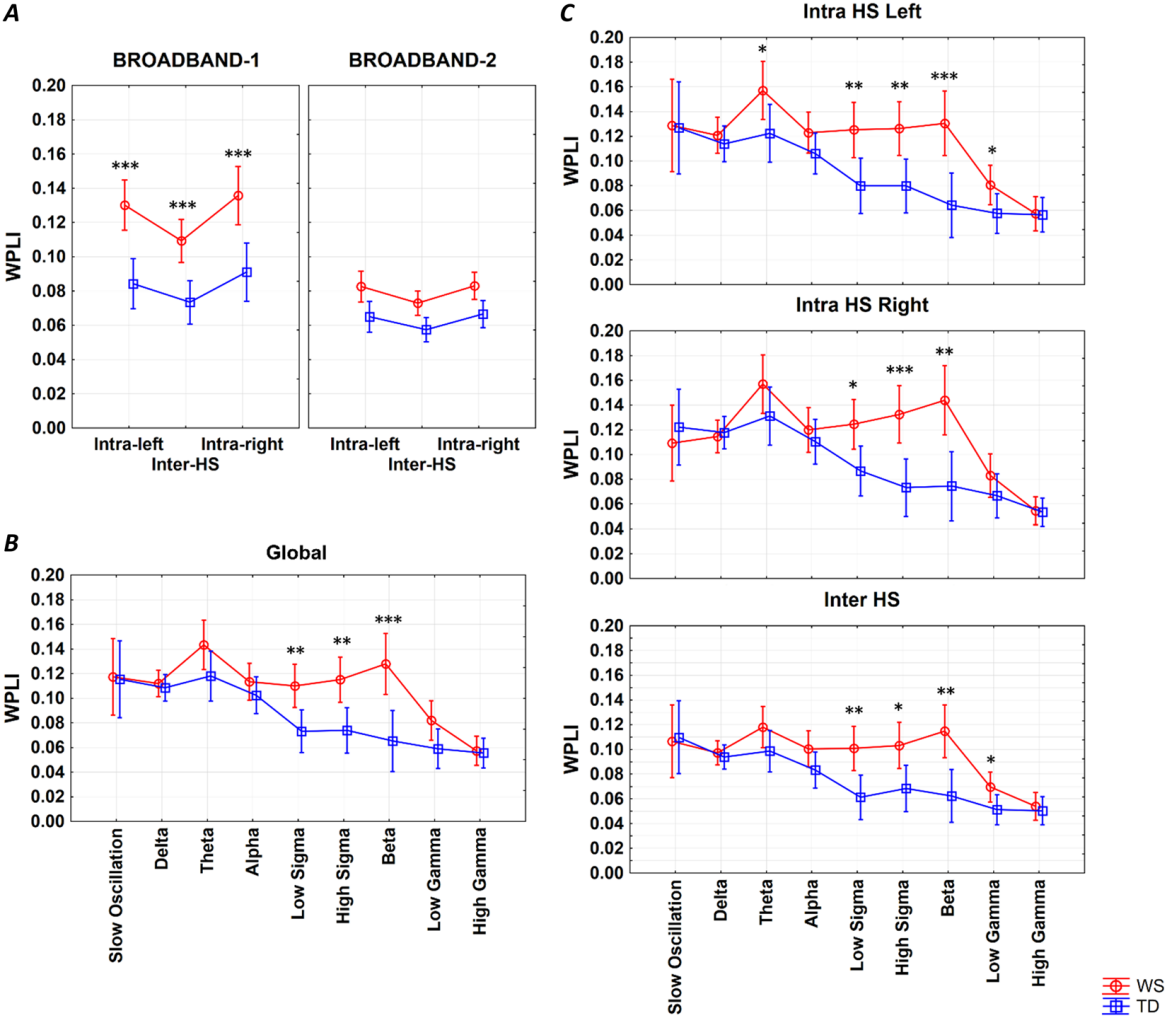
REM sleep EEG WPLI means. (**A**) Intra- and interhemispheric REM sleep EEG broadband-1 and broadband-2 WPLI means of Williams syndrome and typically developing subjects (means and 95% confidence intervals). Intra-left – left intrahemispheric, Inter-HS – interhemispheric, Intra-right – right intrahemispheric. *p < 0.05, **p < 0.01, ***p < 0.001, according to post-hoc Fisher LSD tests following a 3-way ANOVA with repeated measures(Group × Intrahemispheric/Interhemispheric × Band). (**B**) Band-limited global WPLI means of Williams syndrome and typically developing subjects’ REM sleep EEG recordings (means and 95% confidence intervals). *p < 0.05, **p < 0.01, ***p < 0.001, according to post-hoc Fisher LSD tests following a 2-way ANOVA with repeated measures (Group × Band). (**C**) Band-limited intra- and interhemispheric WPLI means of Williams syndrome and typically developing subjects’ REM sleep EEG recordings (means and 95% confidence intervals). *p < 0.05, **p < 0.01, ***p < 0.001, according to one-way ANOVAs.

The overall enhanced broadband connectivity of Williams syndrome subjects (main effect: F = 22.05; d.f. = 1, 38; p < 0.0001) resulted from Williams syndrome vs. typically developing group differences in broadband-1 WPLI (F = 7.58; d.f. = 1, 38; p = 0.0089). Both intrahemispheric (left and right) and interhemispheric broadband-1 connectivity was significantly higher in Williams syndrome as compared to typically developing subjects (results supported by post-hoc tests, see Fig. 4A and Supplementary Table S11). Increased intrahemispheric as compared to interhemispheric connectivity (F = 25.46; d.f. = 2, 76; p < 0.0001), increased broadband-1 as compared to broadband-2 WPLI (F = 49.30; d.f. = 1, 38; p < 0.0001), as well as unequal Intra/inter hemispheric connection differences in the two bands (F = 13.24; d.f. = 2, 76; p < 0.0001) were seen in both groups (Williams syndrome, typically developing).

### Band-limited, spatially unspecific connectivity

The enhanced overall connectivity of Williams syndrome subjects (F = 19.57; d.f. = 1, 38; p < 0.0001) was unevenly distributed among the different frequency bands (Group × Band: F = 2.50; d.f. = 8, 304; p = 0.0118) as follows. Enhanced WPLI values for low (11–12.75 Hz) and high sigma, as well as beta activities were found in Williams syndrome subjects (post-hoc tests, see Fig. 4B and Supplementary Table S12). It has to be mentioned, that different frequency bands were characterized by different levels of connectivity during REM sleep in both groups (main effect of band: F = 11.92; d.f. = 8, 304; p < 0.0001).

### Band-limited intra- and interhemispheric connectivity

Williams syndrome as compared to typically developing subjects were characterized by significantly higher low sigma, high sigma and beta connectivity in both left and right intrahemispheric as well as interhemispheric electrode pairings. In addition, left intrahemispheric theta and low gamma, as well as interhemispheric low gamma WPLI means were significantly increased in the Williams syndrome as compared to the typically developing group (Fig. 4C and Supplementary Table S13).

### Region-specific broadband connectivity

Increased intra-area broadband-1 WPLI of Williams syndrome subjects in all, but one region was revealed. The exception was the O region. In addition, all inter-area broadband-1 WPLI means were shown to be significantly higher in Williams syndrome as compared to typically developing subjects.

**Figure 5:**
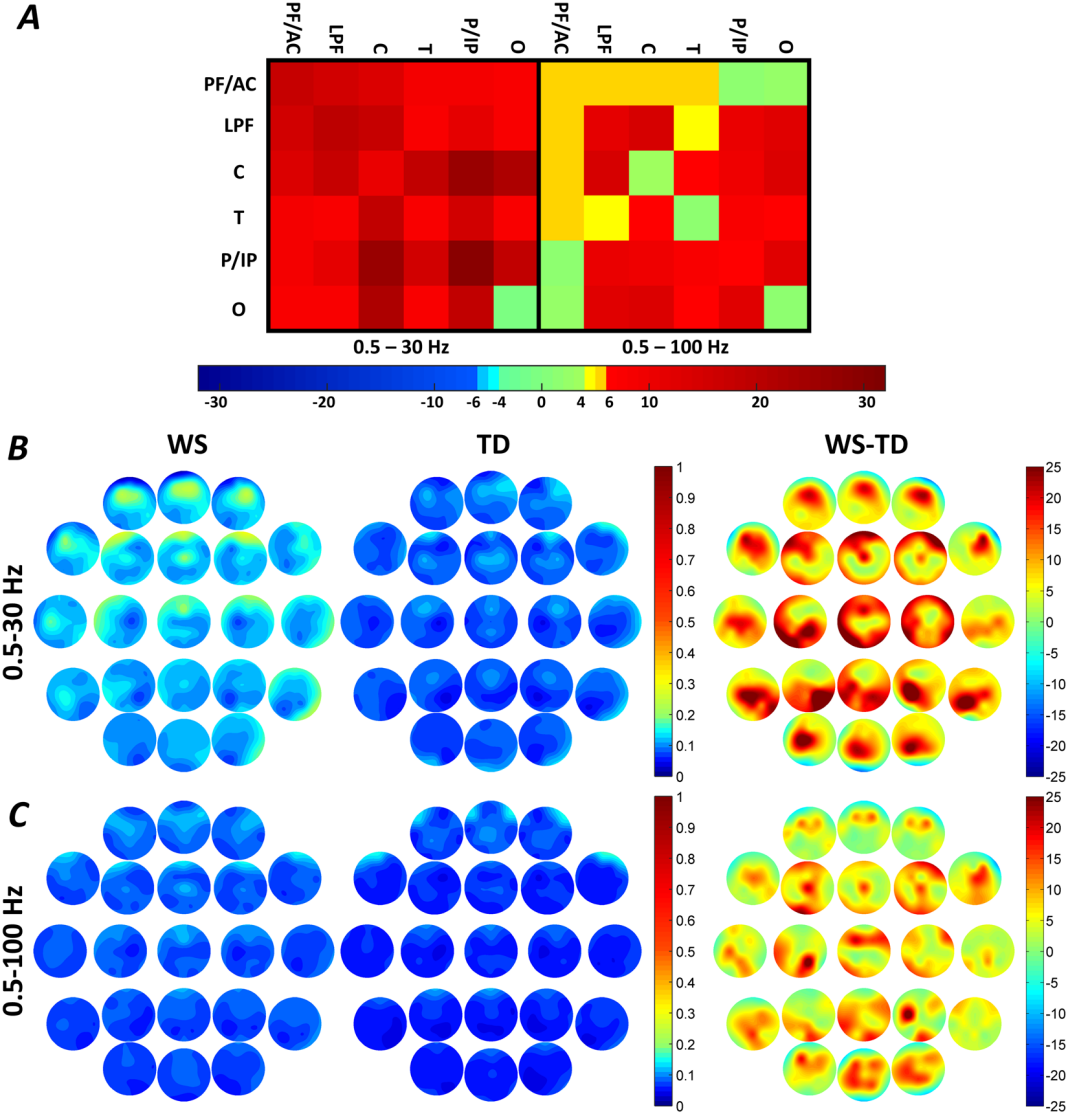
Region-specific REM sleep EEG broadband-1 (0.5–30 Hz) and broadband-2 (0.5–100 Hz) WPLI differences. Broadband-1 WPLIs are significantly higher in Williams syndrome as compared to the typically developing group in several intra- and inter-regional pairings. Color codes: Red = Williams syndrome > typically developing (B-H corrected), Yellow-orange = Williams syndrome > typically developing (uncorrected), Green = Williams syndrome ≈ typically developing, Light blue = Williams syndrome < typically developing (uncorrected), Blue = Williams syndrome < typically developing (B-H corrected). B-C. Broadband-1 and broadband-2 WPLI maps highlighting the patterns of absolute group means (left: Williams syndrome; middle: typically developing) and Williams syndrome-typically developing differences (right). Positions of the maps represent the seed derivation of the synchronization analyses (according to the 10–20 system), while the color patterns are the representations of the variable strengths in the synchronization (WPLI) between the respective region and the seed derivation.

With respect to broadband-2 WPLI means, significant increases in intra-area LPF and P/IP connectivities of Williams syndrome subjects were evidenced. Likewise, increased LPF-C, LPF-P/IP, LPF-O, T-C, T-P/IP and T-O, C-T, C-O inter-regional broadband 2 WPLI means were found in Williams syndrome subjects (Fig. 5 and Supplementary Tables S14, S15 and S16).

### Region specific, band-limited connectivity

#### WPLI decreases of Williams syndrome subjects

**Figure 6:**
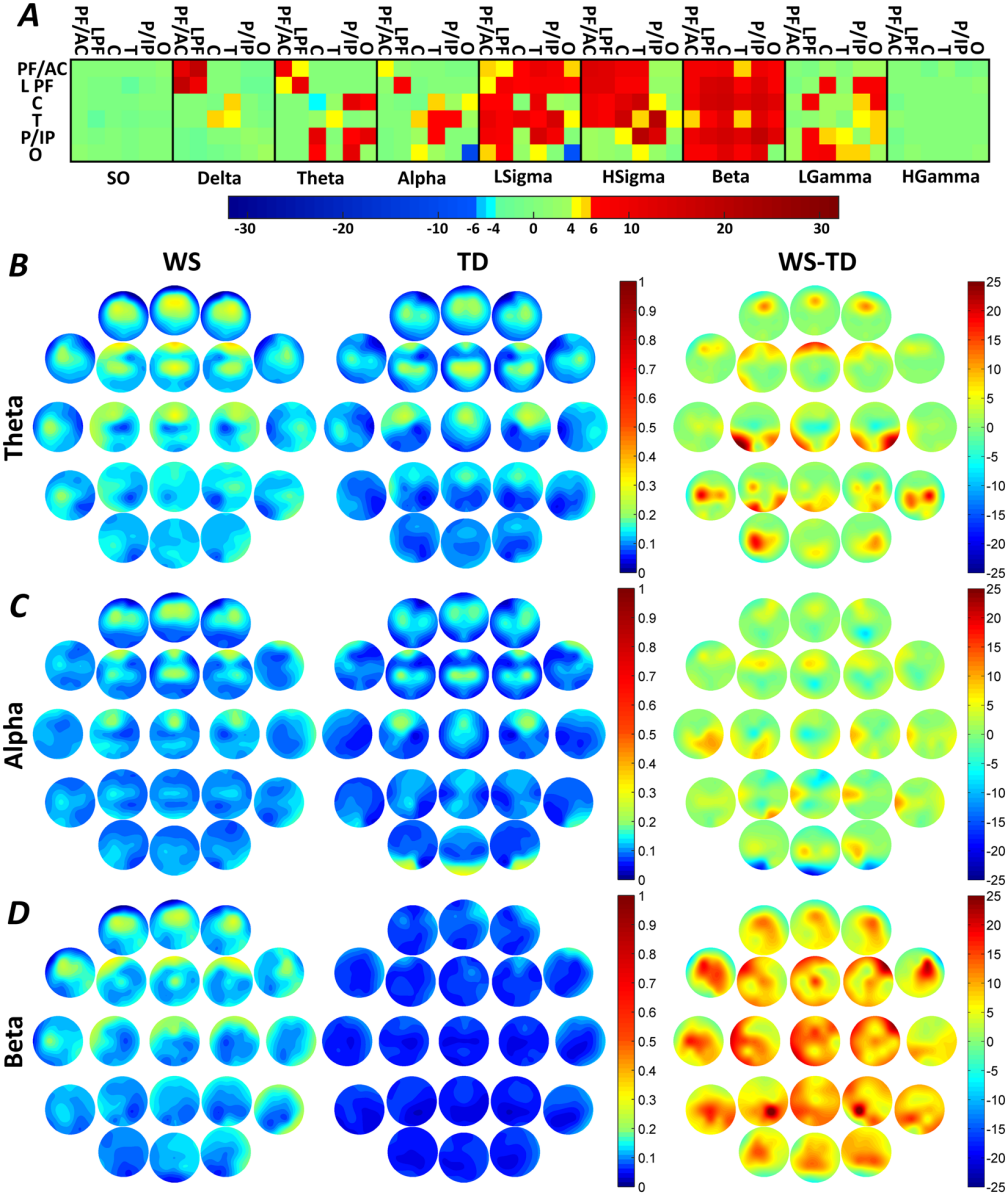
Region-specific and band-limited REM sleep EEG connectivity differences. Frequency range codes: SO = Slow oscillation, LSigma = Low sigma, HSigma = High sigma, LGamma = Low gamma, HGamma = High gamma. Color codes: Red = Williams syndrome > typically developing (B-H corrected), Yellow-orange = Williams syndrome > typically developing (uncorrected), Green = Williams syndrome ≈ typically developing, Light blue = Williams syndrome < typically developing (uncorrected), Blue = Williams syndrome < typically developing (B-H corrected). B-D. Theta, alpha and beta WPLI maps highlighting the patterns of absolute group means (left: Williams syndrome; middle: typically developing) and Williams syndrome-typically developing differences (right). Positions of the maps represents the seed derivation of the synchronization analyses (according to the 10–20 system), while the color patterns are the representations of the variable strengths in the synchronization (WPLI) between the respective region and the seed derivation.

Decrease in O intra-regional WPLI of Williams syndrome subjects was found. The latter effect of decreased WPLI in Williams syndrome subjects was further strengthened by the findings on the WPLI means of the neighboring frequency band, namely, the low sigma range (Fig. 6 and Supplementary Tables S17, S18, S19 and S20).

#### WPLI increases of Williams syndrome subjects

Williams syndrome subjects are characterized by increases in intra-area prefrontal-anterior cingular (PF/AC) and LPF, as well as inter-regional PF/AC-LPF delta WPLI means. The analysis of REM sleep EEG theta WPLI means revealed a significantly increased PF/AC, LPF and P/IP intra-regional connectivity increase in the Williams syndrome group. Likewise, increases in C-P/IP, C-O, as well as P/IP-O inter-regional theta WPLI means were significantly higher in Williams syndrome than in typically developing group (Fig. 6 and Supplementary Tables S17, S18, S19 and S20).Increases in LPF, T, and P/IP intra-regional as well as PF/AC-C, PF/AC-T, PF/AC-P/IP, LPF-T, LPF-C, LPF-P/IP, LPF-O, C-T, and T-P/IP inter-regional REM sleep EEG alpha WPLI means were found in Williams syndrome subjects in spite of a parallel decrease in intra-regional O decrease as reported above.

The high sigma WPLI values indicated a uniform overconnectivity, supported by the significantly increased values of the Williams syndrome group in several regions (PF/AC, LPF, C and P/IP), as well as inter-regional relationships (PF/AC-LPF, PF/AC-C, PF/AC-T, LPF-C, LPF-T, T-C, T-P/IP). Uniform Williams syndrome-specific overconnectivity was indicated by the results of the analyses of REM sleep EEG beta WPLI means.

### Atypical developmental trajectories of sleep-related neural connectivity in Williams syndrome

**Figure 7:**
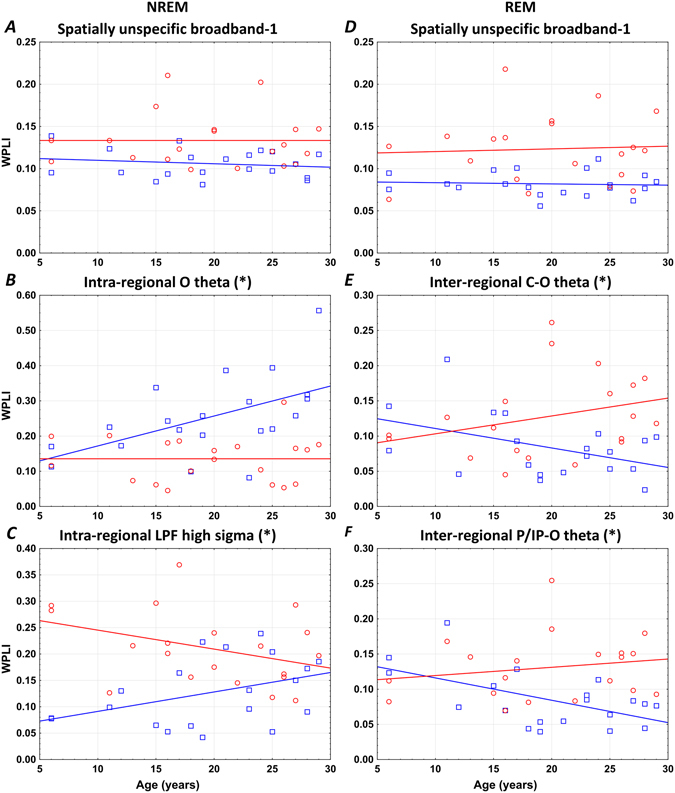
Developmental trajectories of neural connectivity. (*) Denotes significant age × group effects based on homogeneity of slopes analysis indicating altered developmental patterns of sleep-related neural connectivity in Williams syndrome. Left column: NREM sleep, right column: REM sleep. Color codes: Red = Williams syndrome, blue = typically developing. (**A** and **D**) are examples of the 131 age-independent Williams syndrome vs. typically developing group differences in sleep EEG WPLI values. (**B** and **C**) Show the lack of an age-dependent increase in NREM sleep EEG connectivity (intra-area O theta and intra-area LPF high sigma), while (**E** and **F**) reflect the lack of any age-related decrease in REM sleep neural connectivity (inter-area C-O and P/IP-O theta) in Williams syndrome subjects.

In order to test if the above significant Williams syndrome vs typically developing differences in neural connectivity emerge from an altered developmental pattern as revealed for several objective sleep EEG variables^19^, we run a series of homogeneity of slopes analyses with age, group (Williams syndrome, typically developing) and age × group as predictors and EEG synchronization as dependent variables. Out of the 135 significant B-H-corrected findings presented above, only 4 were characterized by a significant age × group effect, indicating altered developmental patterns of sleep-related neural connectivity in Williams syndrome. Two of them were related to the lack of an age-dependent increase in NREM sleep EEG connectivity in Williams syndrome subjects (intra-area O theta and intra-area LPF high sigma), while two were reflecting the lack of any age-related decrease in REM sleep neural connectivity of our atypically developing subjects (inter-area C-O and P/IP-O theta). The remaining 131 variables were characterized by age-independent Williams syndrome vs. typically developing differences as presented above (Fig. 7).

## Discussion

Our new findings indicate a sleep-state independent and broad band increase in sleep EEG synchronization of subjects with Williams syndrome. Reported global interhemispheric and between network overconnectivity of subjects with Williams syndrome is a similar fMRI finding^20, 21^. In addition, the increased number of synapses and dendritic spines in a Williams syndrome model based on human induced pluripotent stem cells^22^ could be the anatomical-microstructural basis of our finding. This assumption is supported by the reported correlations of anatomical and functional connectivity measures in human brain structures^26^ and neural models^27^.

It seems that our data support a general overconnectivity of the Williams syndrome cortex, while more specific aspects are also worth to mention. An important methodological aspect is that EEG recordings, which are much more feasible in subjects with developmental disabilities are suitable for unravelling the peculiarities of network synchronization in Williams syndrome, hitherto established exclusively with fMRI recordings. Another relevant aspect to be emphasized is that the (between network) overconnectivity reported in the two recent fMRI investigations of resting Williams syndrome subjects^20, 21^ can be generalized to NREM and REM sleep as well. This latter aspect of a state-independent neural overconnectivity in Williams syndrome subjects indicates a potential structural basis of the observed sleep alterations, which could be further characterized by neuroimaging methods unravelling the properties of white matter tracts.

In addition to the strong support for a broadband overconnectivity of neural networks in Williams syndrome subjects, specific DMN hubs and networks are underconnected in this developmental disability^20, 21^. Indeed, we have found sleep state-, frequency-, and region-specific decreases in EEG synchronization in our current study. Globally decreased NREM sleep EEG alpha connectivity with an apparent center of gravity in the LPF-T inter-regional interrelationship is evidenced. We found NREM sleep EEG alpha power decreases in Williams syndrome subjects^8, 10, 12, 19^. Reports on the close relationship between resting state alpha global phase synchrony and DMN activity^29^, as well as alpha power and presumed DMN activity^30^ have also been published. Thus, it is reasonable to assume that decreased NREM sleep EEG alpha synchronization reflects the decreased connectivity between DMN hubs as reported in previous fMRI studies. NREM sleep EEG alpha activity is part of the ongoing fluctuation of arousal level during sleep^31^, while its topography and cortical sources closely correspond to the posterior hubs of the DMN^32^. Other sources of Williams syndrome-related neural underconnectivity are related to the NREM sleep slow oscillation, as well as to intra-regional O theta and alpha/low sigma activities during NREM and REM sleep, respectively. The O region and the related visual functions are known to be affected by the atypical developmental processes inherent to Williams syndrome^23–25^. Here we provide sleep-related neurophysiological evidence for this altered functional organization in Williams syndrome. The coexistence of functional over- and underconnectivity of neural structures has been shown to characterize other developmental disorders as well, among which autism spectrum disorder is the most striking example^15^. Findings on sleep EEG connectivity in autism spectrum disorder are mixed, suggesting under- and over-connectivity in different settings, measures and studies^18, 33, 34^. However, a reduced right fronto-cortical connectivity (including broadband effects) was more consistently reported during both NREM and REM sleep of subjects with autism spectrum disorder^18, 33^. In contrast, our current study on Williams syndrome participants revealed an increased connectivity during sleep in this region. In other words, our contrasting findings in Williams syndrome and autism support the assumption that the two neurodevelopmental phenomena are situated at the opposite ends of the same phenotypic axis^35^.

Down syndrome is another case of genetically determined atypical development characterized by altered neural microstructure. Subjects with Down syndrome were shown to express lower levels of coherence (connectivity) in the wake EEG alpha band oscillations (which is similar to the decreased alpha connectivity in our Williams syndrome participants), whereas other frequencies are characterized by more or less overconnectivity in a state and age-dependent manner^36^. Although no sleep recordings were analyzed in the latter study, the findings indicate a connectivity profile revealing similarities with the Williams syndrome population.

Evidence suggests the atypical developmental trajectories of several phenotypic traits in Williams syndrome. Among these traits sleep architecture is one prominent example^19^, which is highly relevant from our current perspective. Our tests of the parallelism/divergence of the age-trends in sleep EEG synchronization provided mixed results. We did not intend to test the development of sleep-dependent neural synchronization in typically developing subjects. Instead we focused on the significant deviation of the age-effects in sleep EEG synchronization of Williams syndrome as compared to typically developing subjects, by testing the age × group interactions in our statistical models. The majority of the functional connectivity differences (131 out of 135) between the Williams syndrome and typically developing groups were not influenced by age (Fig. 7A,D). The remaining 4 significant age × group effects are worth mention, however. Two of them are related to intra-regional connectivity of NREM sleep, while two to inter-regional connectivity of REM sleep. In addition, three of the significant age × group effects were related to the O region. First, we found a lack of an age-dependent increase in NREM sleep EEG intra-regional O theta synchronization of Williams syndrome subjects, in contrast to the steady increase of this measure in the typically developing group (Fig. 7B). This finding might indicate a persistent disruption of the development of visual functions in Williams syndrome subjects. Our second finding indicate a persistent (and premature), age-independent overconnectivity in LPF high sigma activity of Williams syndrome subjects (Fig. 7C). This pattern contrasts the gradual, age-dependent increase of this index in typically developing individuals. Both of the REM sleep-related atypical age-trends (Fig. 7E,F) were related to the age-independence of EEG inter-regional O theta synchronization in Williams syndrome subjects, as compared to the significant age-dependent decrease in typically developing subjects. These latter findings might reflect a compensatory maintenance of inter-regional O connectivity (Fig. 7E,F) as a reaction to the lack of developmental increase in intra-regional O connectivity.

To summarize, we revealed a striking overall connectivity as measured by WPLI in sleep EEG of Williams syndrome subjects. This overconnectivity might indicate the need for considering Williams syndrome as a neurodevelopmental disorder, characterized by synaptic underpruning. Sleep deficits of subjects with Williams syndrome^5–10, 19^ might contribute to this undepruning. In addition, specific regional decreases in sleep EEG connectivity are found in O-O relationships as well as overall alpha frequency phase synchronization. The latter is consistent with the defective primary visual/visuospatial functions (including cortical anatomical alterations) and decreased DMN synchronization of Williams syndrome subjects.

## Methods

### Ethics statement

The research protocol was approved by the Social Sciences Ethical Review Board of the Budapest University of Technology and Economics and was conducted according to the approved guidelines. Adult participants or the parents of the underage participants signed informed consent for the participation in the study according to the Declaration of Helsinki.

### Subjects and genetic investigations

Williams syndrome participants (N = 20, 7 males and 13 females, age range 6–29 years, mean age ± standard deviation: 19.6 ± 7.07 years) were contacted through the Hungarian Williams Syndrome Association (parents were mediating in the case of underage subjects). All Williams syndrome subjects (including adults) were living with their parents. Typically developing controls (N = 20, 6 males and 14 females, age range 6–29 years, mean age ± standard deviation: 19.6 ± 7.01 years) were selected by personal contacts of the authors and matched by age and sex to the Williams syndrome participants (see Supplementary Table S21). A twin pair discordant for Williams syndrome and sex^12^ was considered as a pair case control.

The clinical diagnosis of Williams syndrome was established prior to this study by fluorescent *in situ* hybridization (FISH) test demonstrating the hemideletion of the elastin gene. To confirm the clinical diagnosis and specify the size of the hemideleted region, we carried out multiplex ligation-dependent probe amplification (MLPA) using the SALSA MLPA KIT P029- A1 (MRC-Holland, Amsterdam, The Netherlands). Briefly, pairs of locus-specific oligonucleotide probes are hybridized to the target DNA, followed by a ligation and amplification step. Amplified fragments are separated and analyzed using capillary electrophoresis. At the resolution of this genotyping method, all Williams syndrome subjects were carriers of a typical deletion spanning at least 1.038 Mb between *FKBP6* (7:72742167-72772634) and *CLIP2* (7:73703805-73820273)^19^.

Exclusion criteria for typically developing participants were medical diagnoses of sleep problems or psychiatric, neurological or other medical disorders. As Williams syndrome is a rare disease, our strategy was to include as many Williams syndrome subjects as possible, and document any individual specificity. Participants were free of drugs except for 1 Williams syndrome patient who was on stable medication with clonidine (150 mg/day), enalapril (5 mg/day), acetylsalicylic acid (500 mg/day), betaxololi hydrochloridum (20 mg/day), and amlodipine (15 mg/day). Those patients not on medication were not withdrawn from any pharmacological treatment prior to the study.

### Experimental design

Subjects’ sleep was recorded at their homes by using ambulatory home polysomnography. Sleep recordings on two consecutive weekend nights were performed according to the subjects’ sleeping habits. We used a portable 32 channel SD LTM Headbox together with a BRAIN QUICK System PLUS software (Micromed, Italy) for polysomnographic data recording. We recorded EEG according to the 10–20 system^37^ at 21 recording sites (Fp1, Fp2, Fpz, F3, F4, F7, F8, Fz, C3, C4, Cz, P3, P4, Pz, T3, T4, T5, T6, O1, O2, Oz) referred to the mathematically linked mastoids. Bipolar EOG, ECG and submental as well as tibialis EMG were also recorded. We have not recorded respiratory variables because there was no indication for any significant breathing difficulty during sleep in the study by Arens *et al*.^5^, and in a more recent study by Mason *et al*.^7^. Moreover, this would have caused further inconveniences for the participants because of the recording instruments. However, one participant, whose sleep was one of the most fragmented in our study, underwent a full-night polysomnography in a clinical sleep laboratory before our examination. The results of clinical respiratory monitoring did not show sleep apnea or hypopnea in this participant. EEG and polygraphic data were high-pass filtered at 0.15 Hz and low-pass filtered at 1500 Hz (both 40 dB/decade). Data were collected with an analogue to digital conversion rate of 4096 Hz/channel (synchronous, 16 bit). A further 40 db/decade anti-aliasing digital filter was applied by digital signal processing (firmware) which low pass filtered the data at 124 Hz. Subsequently, the digitized and filtered EEG was downsampled and stored at a sampling rate of 1024 Hz. Sleep recordings were visually scored in 20 second epochs and 4 second epochs containing artifactual sleep EEG (movement, sweating or technical artifacts) based on the visual inspection of the records were manually removed before further analyses.

### Statistical analysis

The WPLI of two signals is defined as the phase leads or lags weighted by the magnitude of the imaginary part of the complex cross spectrum of the series. Mean WPLIs^28^ of electrode pairs (all possible combinations based on 4 second long epochs) from all-night NREM and REM sleep EEG records (21 mathematically linked mastoid-referred derivations of the 10-20 system sampled at 1024 Hz/channel) of our Williams syndrome and typically developing subjects were calculated for standard frequency bands as follows: slow oscillation (0.5–1 Hz), as well as delta (1.25–4.5 Hz), theta (4.75–7.25 Hz), alpha (7.5–10.75 Hz), low sigma (11–12.75 Hz), high sigma (13–15 Hz), beta (15.25–30 Hz), low gamma (30.25–50 Hz) and high gamma (50.25–100 Hz) activities. In addition, broadband-1 (0.5–30 Hz) and broadband-2 (0.5–100 Hz) WPLI values were also calculated.

**Figure 8:**
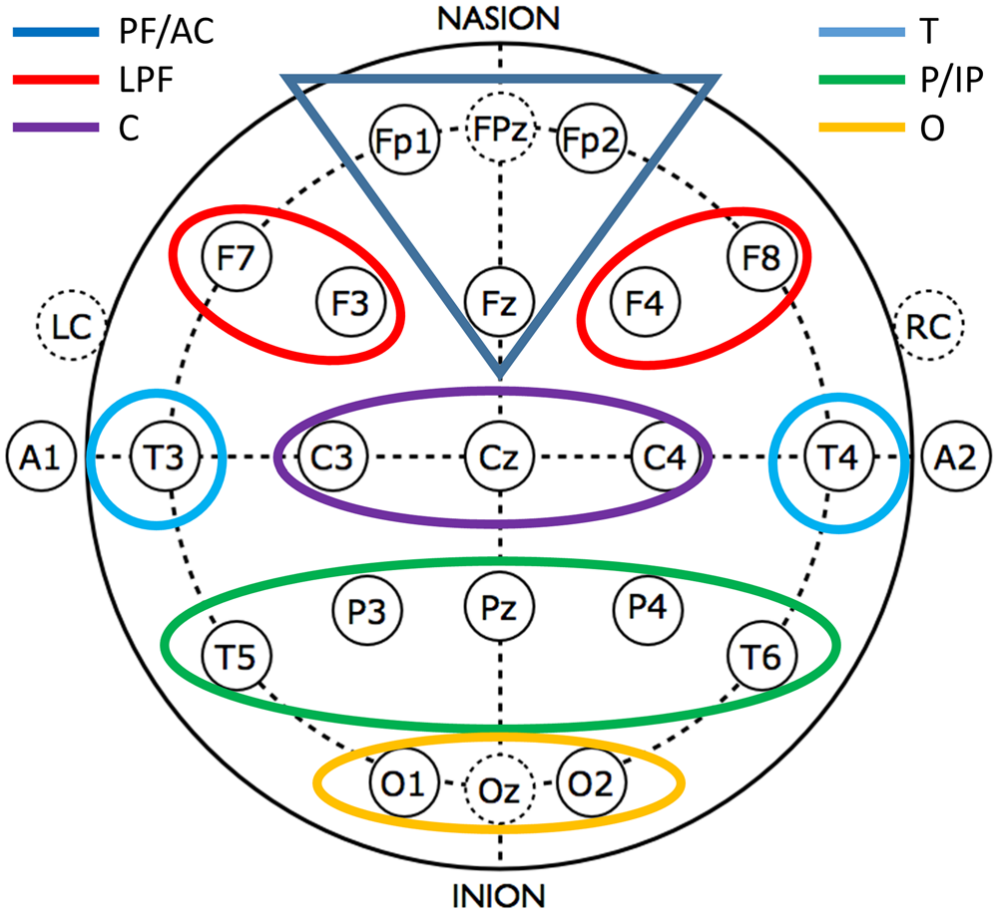
Areas defined for the region-specific analyses of WPLI.

All possible pairings of the 21 EEG derivations results in an inflation of Type – I error. In order to reduce the number of statistical tests, we followed a top-down approach by using consecutive tests progressing from global broadband to local and frequency-specific effects. At each of these steps, we applied either a 2 or a 3-way ANOVA (Group × Band or Group × Band × Inter/Intrahemispheric connections, depending on the number of variables) with repeated measures integrated with Fisher Least Square Differences (LSD) post-hoc analyses (steps 1–3, see below), or a series of one-way ANOVA-s completed with the Benjamini-Hochberg (B-H) method^38^ for controlling the false discovery rate (steps 4–6). The steps were the following (for both NREM and REM sleep):

1. Analyzing mean WPLI of all derivation pairs (spatial unspecificity) at the broadband scales.
2. Analyzing mean intra- and interhemispheric connectivity at the broadband scales.
3. Analyzing the spatially unspecific (all derivation pairs included) means of frequency-specific WPLI values in the above defined frequency bands.
4. Analyzing intra- and interhemispheric connectivity means of frequency-specific WPLI values in the above defined frequency bands.
5. Analyzing region-specific, broadband WPLI means.
6. Analyzing frequency band- and region-specific WPLI means. Region-specific values are defined as intra-region and inter-region connectivity by using the following pre-defined areas (Fig. 8): Prefrontal-anterior cingular (PF/AC: Fp1, Fp2, Fz), Lateral prefrontal (LPF: F3, F4, F7, F8), Central (C: C3, C4, Cz), Temporal (T: T3, T4), Parietal-inferior parietal (P/IP: P3, P4, Pz, T5, T6), and Occipital (O: O1, O2, Oz). WPLI values characterizing intra- and inter-regional connectivity were defined as means of the respective derivation-pair WPLIs.

In order to test if atypical age-dependent changes contribute to the group differences in EEG synchronization, the B-H-corrected, significant Williams syndrome vs typically developing group differences in sleep EEG WPLI values were subjected to a homogeneity of slopes analysis as follows: age, group (Williams syndrome, typically developing) and age × group were predictors, while the EEG connectivity values were dependent variables. Only the age × group effects are highlighted in this report.

## Electronic supplementary material

Supplementary info

## References

[1] Boivin MJ, Kakooza AM, Warf BC, Davidson LL, Grigorenko EL (2015). Reducing neurodevelopmental disorders and disability through research and interventions. Nature.

[2] Meyer-Lindenberg A, Mervis CB, Faith Berman K (2006). Neural mechanisms in Williams syndrome: a unique window to genetic influences on cognition and behaviour. Nat. Rev. Neurosci..

[3] Järvinen-Pasley, A. *et al*. Defining the social phenotype in Williams syndrome: A model for linking gene, the brain, and behavior. *Dev. Psychopathol*. **20** (2008). 10.1017/S0954579408000011 PMC2892602 18211726

[4] Angriman M, Caravale B, Novelli L, Ferri R, Bruni O (2015). Sleep in Children with Neurodevelopmental Disabilities. Neuropediatrics.

[5] Arens R (1998). Periodic limb movement in sleep in children with Williams syndrome. J. Pediatr..

[6] Goldman SE, Malow BA, Newman KD, Roof E, Dykens EM (2009). Sleep patterns and daytime sleepiness in adolescents and young adults with Williams syndrome. J. Intellect. Disabil. Res..

[7] Mason TBA (2011). Sleep in children with Williams Syndrome. Sleep Med..

[8] Gombos F, Bódizs R, Kovács I (2011). Atypical sleep architecture and altered EEG spectra in Williams syndrome. J. Intellect. Disabil. Res..

[9] Annaz D, Hill CM, Ashworth A, Holley S, Karmiloff-Smith A (2011). Characterisation of sleep problems in children with Williams syndrome. Res. Dev. Disabil..

[10] Bódizs R, Gombos F, Kovács I (2012). Sleep EEG fingerprints reveal accelerated thalamocortical oscillatory dynamics in Williams syndrome. Res. Dev. Disabil..

[11] Ashworth A, Hill CM, Karmiloff-Smith A, Dimitriou D (2013). Cross syndrome comparison of sleep problems in children with Down syndrome and Williams syndrome. Res. Dev. Disabil..

[12] Bódizs R (2014). Sleep-EEG in dizygotic twins discordant for Williams syndrome. Ideggyogy. Sz..

[13] Kurth S, Achermann P, Rusterholz T, LeBourgeois M (2013). Development of Brain EEG Connectivity across Early Childhood: Does Sleep Play a Role?. Brain Sci..

[14] Tarokh L, Carskadon MA, Achermann P (2014). Early Adolescent Cognitive Gains Are Marked by Increased Sleep EEG Coherence. PLoS One.

[15] Hahamy A, Behrmann M, Malach R (2015). The idiosyncratic brain: distortion of spontaneous connectivity patterns in autism spectrum disorder. Nat. Neurosci..

[16] Dinstein I (2011). Disrupted Neural Synchronization in Toddlers with Autism. Neuron.

[17] Lemieux L, Daunizeau J, Walker MC (2011). Concepts of Connectivity and Human Epileptic Activity. Front. Syst. Neurosci..

[18] Lázár AS (2010). Reduced fronto-cortical brain connectivity during NREM sleep in Asperger syndrome: an EEG spectral and phase coherence study. Clin. Neurophysiol..

[19] Bódizs R (2014). Aging and sleep in Williams syndrome: Accelerated sleep deterioration and decelerated slow wave sleep decrement. Res. Dev. Disabil..

[20] Vega JN, Hohman TJ, Pryweller JR, Dykens EM, Thornton-Wells TA (2015). Resting-State Functional Connectivity in Individuals with Down Syndrome and Williams Syndrome Compared with Typically Developing Controls. Brain Connect..

[21] Sampaio A (2016). Altered functional connectivity of the default mode network in Williams syndrome: a multimodal approach. Dev. Sci..

[22] Chailangkarn T (2016). A human neurodevelopmental model for Williams syndrome. Nature.

[23] Galaburda AM, Holinger DP, Bellugi U, Sherman GF (2002). Williams syndrome: neuronal size and neuronal-packing density in primary visual cortex. Arch. Neurol..

[24] Reiss AL (2000). IV. Neuroanatomy of Williams syndrome: a high-resolution MRI study. J. Cogn. Neurosci..

[25] Gervan P, Gombos F, Kovacs I (2012). Perceptual Learning in Williams Syndrome: Looking Beyond Averages. PLoS One.

[26] Ford JH, Kensinger EA (2014). The relation between structural and functional connectivity depends on age and on task goals. Front. Hum. Neurosci..

[27] Stam CJ (2016). The relation between structural and functional connectivity patterns in complex brain networks. Int. J. Psychophysiol..

[28] Vinck M, Oostenveld R, van Wingerden M, Battaglia F, Pennartz CMA (2011). An improved index of phase-synchronization for electrophysiological data in the presence of volume-conduction, noise and sample-size bias. Neuroimage.

[29] Jann K (2009). BOLD correlates of EEG alpha phase-locking and the fMRI default mode network. Neuroimage.

[30] Knyazev GG, Slobodskoj-Plusnin JY, Bocharov AV, Pylkova LV (2011). The default mode network and EEG alpha oscillations: An independent component analysis. Brain Res..

[31] McKinney SM, Dang-Vu TT, Buxton OM, Solet JM, Ellenbogen JM (2011). Covert Waking Brain Activity Reveals Instantaneous Sleep Depth. PLoS One.

[32] Ferri R, Bruni O, Miano S, Terzano MG (2005). Topographic mapping of the spectral components of the cyclic alternating pattern (CAP). Sleep Med..

[33] Léveillé C (2010). Enhanced connectivity between visual cortex and other regions of the brain in autism: a REM sleep EEG coherence study. Autism Res..

[34] Buckley AW (2015). State-Dependent Differences in Functional Connectivity in Young Children With Autism Spectrum Disorder. EBioMedicine.

[35] Fishman I, Yam A, Bellugi U, Lincoln A, Mills D (2011). Contrasting patterns of language-associated brain activity in autism and Williams syndrome. Soc. Cogn. Affect. Neurosci..

[36] Schmid RG, Tirsch WS, Rappelsberger P, Weinmann H-M, Pöppl SJ (1992). Comparative coherence studies in healthy volunteers and Down’s syndrome patients from childhood to adult age. Electroencephalogr. Clin. Neurophysiol..

[37] Jasper H (1958). Report of the committee on methods of clinical examination in electroencephalography. Electroencephalogr. Clin. Neurophysiol..

[38] Benjamini Y, Hochberg Y (1995). Controlling the False Discovery Rate: A Practical and Powerful Approach to Multiple Controlling the False Discovery Rate: a Practical and Powerful Approach to Multiple Testing. J. R. Stat. Soc..

