# Supplementary material for: Increased overall cortical connectivity with syndrome specific local decreases suggested by atypical sleep-EEG synchronization in Williams syndrome
Source: Sci Rep. 2017 Jul 21;7:6157. doi: 10.1038/s41598-017-06280-2 (PMC5522417; doi:10.1038/s41598-017-06280-2)
Supplement: Supplementary file 1 — Supplementary info [file 41598_2017_6280_MOESM1_ESM.pdf]

Supplementary information

Increased overall cortical connectivity with syndrome specific local decreases suggested by atypical sleep-EEG synchronization in Williams syndrome

Ferenc Gombos[1,†], Róbert Bódizs[1,2,†], Ilona Kovács[1,*]

[1]*Department of General Psychology, Institute of Psychology, Pázmány Péter Catholic University, 1088 Budapest, Hungary;* [2]*Institute of Behavioural Sciences, Semmelweis University, 1089 Budapest, Hungary*

***Correspondence:**

Prof. Dr. Ilona Kovács, Department of General Psychology, Institute of Psychology, Pázmány Péter Catholic University, 1088 Budapest, Mikszáth square 1., Tel.: +(36 1) 327-5927. E-mail: kovacs.ilona@btk.ppke.hu

[†] These authors contributed equally to this work

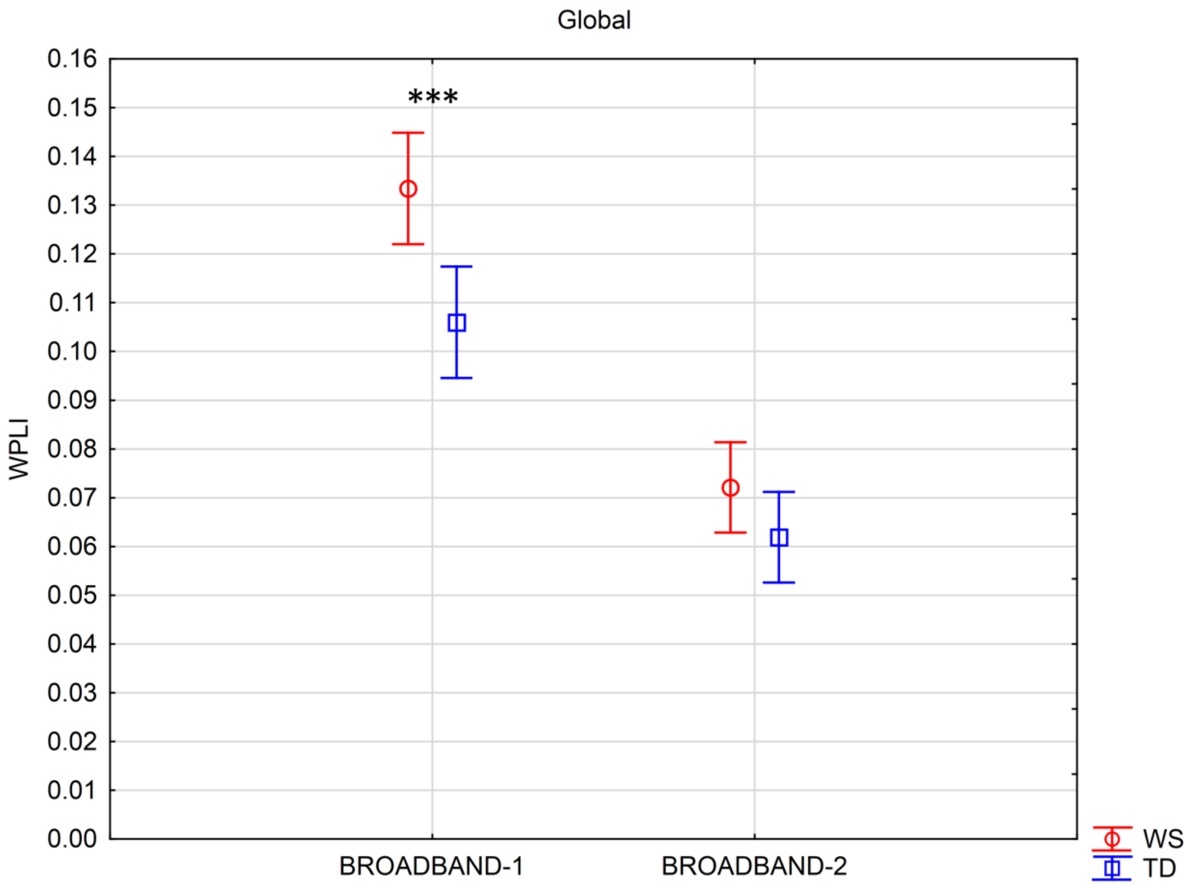

Supplementary Figure S1. Global NREM sleep WPLI means in broadband-1 and broadband-2 frequency ranges of Williams syndrome and typically developing subjects (means and 95% confidence intervals). * p < .05, ** p < .01, according to post-hoc Fisher LSD tests.

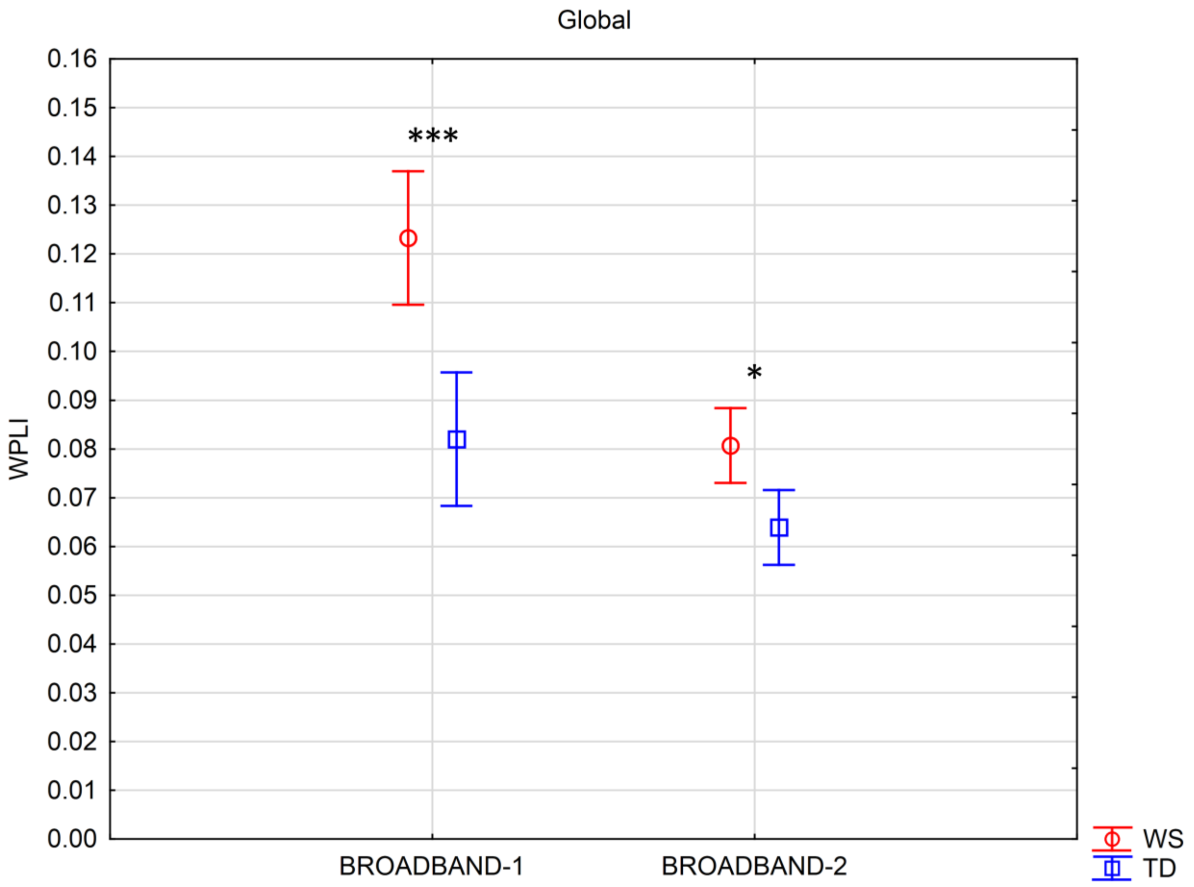

Supplementary Figure S2. Global REM sleep WPLI means in broadband-1 and broadband-2 frequency ranges of Williams syndrome and typically developing subjects (means and 95% confidence intervals). * p < .05, ** p < .01, according to post-hoc Fisher LSD tests.

Supplementary Table S1. Probabilities for Fisher LSD Post Hoc Tests following a 3-way ANOVA with repeated measures (Group × Intrahemispheric/Interhemispheric × Band) conducted on intra- and interhemispheric NREM sleep EEG broadband-1 and broadband-2 WPLI means of Williams syndrome and typically developing subjects. Significant differences are highlighted in red.

NREM	Intra-left	Intra-right	Inter-HS
Broadband-1	0.0017	0.0077	0.0047
Broadband-2	0.2268	0.4057	0.3256

Supplementary Table S2. Probabilities for Fisher LSD Post Hoc Tests following a 2-way ANOVA with repeated measures (Group × Band) conducted on band-limited NREM sleep EEG global WPLI means of Williams syndrome and typically developing subjects. Significant differences are highlighted in red.

NREM	Fisher LSD p value
Slow oscillation	0.133622
Delta	0.447060
Theta	0.004117
Alpha	0.016596
Low sigma	0.069662
High sigma	0.000001
Beta	0.001681
Low gamma	0.160108
High gamma	0.889726

Supplementary Table S3. Probabilities for Fisher LSD Post Hoc Tests following a one-way ANOVA with repeated measures conducted on band-limited NREM sleep EEG intra- and interhemispheric WPLI means of Williams syndrome and typically developing subjects. Significant differences are highlighted in red.

NREM	Intra-left	Intra-right	Inter-HS
Slow oscillation	0.176663	0.060214	0.250404
Delta	0.069033	0.207716	0.319045
Theta	0.000000	0.000107	0.000115
Alpha	0.000049	0.000000	0.014964
Low sigma	0.006385	0.000017	0.136612
High sigma	0.000000	0.000000	0.000000
Beta	0.000001	0.000000	0.000008
Low gamma	0.018291	0.131700	0.108400
High gamma	0.794152	0.685882	0.792612

Supplementary Table S4. Signed F-values and probabilities of region-specific NREM sleep EEG Broadband-1 (0.5-30 Hz) and broadband-2 (0.5-100 Hz) WPLI differences between Williams syndrome and typically developing subjects (intra- and inter-regional pairings). Color codes: Red = WS > TD (B-H corrected), Yellow = WS > TD (uncorrected), White = WS ≈ typically developing, Light blue = WS < TD (uncorrected), Blue = WS < TD (B-H corrected).

NREM		PF/AC		LPF		C		T		P/IP		O	
		Sign. F	p	Sign. F	p	Sign. F	p	Sign. F	p	Sign. F	p	Sign. F	p
Broadband-1	PF/AC	4.236	0.046	8.329	0.006	9.651	0.004	3.386	0.074	8.710	0.005	5.216	0.028
	LPF	8.329	0.006	10.817	0.002	11.155	0.002	1.510	0.227	10.349	0.003	5.772	0.021
	C	9.651	0.004	11.155	0.002	7.719	0.008	1.804	0.187	10.876	0.002	9.746	0.003
	T	3.386	0.074	1.510	0.227	1.804	0.187	3.676	0.063	6.439	0.015	4.587	0.039
	P/IP	8.710	0.005	10.349	0.003	10.876	0.002	6.439	0.015	10.447	0.003	8.174	0.007
	O	5.216	0.028	5.772	0.021	9.746	0.003	4.587	0.039	8.174	0.007	-0.226	0.637
Broadband-2	PF/AC	0.728	0.399	2.986	0.092	5.173	0.029	0.318	0.576	3.816	0.058	4.158	0.048
	LPF	2.986	0.092	1.080	0.305	5.178	0.029	0.130	0.720	4.129	0.049	3.862	0.057
	C	5.173	0.029	5.178	0.029	0.675	0.416	-0.010	0.921	2.553	0.118	4.046	0.051
	T	0.318	0.576	0.130	0.720	-0.010	0.921	0.001	0.982	0.157	0.694	0.527	0.472
	P/IP	3.816	0.058	4.129	0.049	2.553	0.118	0.157	0.694	0.482	0.492	1.181	0.284
	O	4.158	0.048	3.862	0.057	4.046	0.051	0.527	0.472	1.181	0.284	-0.013	0.911

Supplementary Table S5. Group means, signed F-values and probabilities of region-specific NREM sleep EEG Broadband-1 (0.5-30 Hz) WPLI differences of Williams syndrome and typically developing subjects in all the possible electrode pairings. Color codes: Red = WS > TD (B-H corrected), Yellow = WS > TD (uncorrected), White = WS ≈ typically developing, Light blue = WS < TD (uncorrected), Blue = WS < TD (B-H corrected).

Broadband-1	Mean WS	Mean TD	Signed F	p
Fp2-Fp2				
Fp2-F8	0.1730	0.1553	-0.3390	0.5638
Fp2-T4	0.2501	0.1895	-3.7856	0.0591
Fp2-T6	0.1747	0.1832	0.0813	0.7771
Fp2-O2	0.1165	0.1315	0.3319	0.5679
Fp2-Fp1	0.0831	0.0901	0.1714	0.6812
Fp2-F7	0.1164	0.1193	0.0140	0.9064
Fp2-T3	0.1767	0.1700	-0.0454	0.8324
Fp2-T5	0.1593	0.1849	0.5920	0.4464
Fp2-O1	0.1366	0.1261	-0.2026	0.6552
Fp2-F4	0.1501	0.1256	-0.6557	0.4231
Fp2-C4	0.1281	0.1240	-0.0239	0.8780
Fp2-P4	0.1250	0.1592	1.4030	0.2436
Fp2-F3	0.1464	0.1164	-1.4472	0.2364
Fp2-C3	0.1148	0.1233	0.1201	0.7308
Fp2-P3	0.1290	0.1560	0.8919	0.3509
Fp2-Fpz1	0.1111	0.1101	-0.0022	0.9632
Fp2-Fz	0.1723	0.1247	-2.9051	0.0965
Fp2-Cz	0.1425	0.0968	-3.7454	0.0604
Fp2-Pz	0.1181	0.1379	0.7500	0.3919
Fp2-Oz	0.1552	0.1097	-3.1475	0.0841
F8-Fp2	0.1730	0.1553	-0.3390	0.5638
F8-F8				
F8-T4	0.2583	0.2085	-0.8889	0.3517
F8-T6	0.1889	0.1893	0.0001	0.9927
F8-O2	0.1562	0.1306	-0.5575	0.4599
F8-Fp1	0.1477	0.1204	-1.1181	0.2970
F8-F7	0.1459	0.1012	-2.0134	0.1641
F8-T3	0.1687	0.1483	-0.3302	0.5689
F8-T5	0.1989	0.1569	-0.9597	0.3335
F8-O1	0.1628	0.1130	-2.9997	0.0914
F8-F4	0.2510	0.1134	-15.3964	0.0004
F8-C4	0.1663	0.1184	-1.3584	0.2511
F8-P4	0.1357	0.1766	2.4241	0.1278
F8-F3	0.1915	0.1002	-7.9102	0.0077
F8-C3	0.1460	0.1140	-0.9161	0.3446
F8-P3	0.1271	0.1588	0.9532	0.3351
F8-Fpz1	0.1718	0.1026	-7.3571	0.0100
F8-Fz	0.1956	0.0981	-13.4252	0.0008
F8-Cz	0.1603	0.0865	-13.7054	0.0007
F8-Pz	0.1137	0.1484	1.7680	0.1916
F8-Oz	0.1824	0.0991	-7.3896	0.0098
T4-Fp2	0.2501	0.1895	-3.7856	0.0591
T4-F8	0.2583	0.2085	-0.8889	0.3517
T4-T4				
T4-T6	0.1197	0.1466	1.1910	0.2820
T4-O2	0.1167	0.1305	0.3693	0.5470
T4-Fp1	0.2203	0.1707	-2.3670	0.1322
T4-F7	0.2040	0.1581	-1.3823	0.2470
T4-T3	0.1716	0.1124	-3.8085	0.0584

Broadband-1	Mean WS	Mean TD	Signed F	p
T4-T5	0.1385	0.1069	-0.9640	0.3324
T4-O1	0.1366	0.1076	-1.6660	0.2046
T4-F4	0.3298	0.1961	-14.6687	0.0005
T4-C4	0.2471	0.1455	-12.2071	0.0012
T4-P4	0.1302	0.1193	-0.2339	0.6315
T4-F3	0.2649	0.1761	-7.5305	0.0092
T4-C3	0.2059	0.1378	-6.7259	0.0134
T4-P3	0.1306	0.0960	-2.1856	0.1475
T4-Fpz1	0.2382	0.1730	-3.4788	0.0699
T4-Fz	0.2903	0.1856	-8.8430	0.0051
T4-Cz	0.2597	0.1308	-23.5717	0.0000
T4-Pz	0.1418	0.0966	-3.7210	0.0612
T4-Oz	0.1407	0.1087	-1.6301	0.2094
T6-Fp2	0.1747	0.1832	0.0813	0.7771
T6-F8	0.1889	0.1893	0.0001	0.9927
T6-T4	0.1197	0.1466	1.1910	0.2820
T6-T6				
T6-O2	0.1321	0.1271	-0.0276	0.8689
T6-Fp1	0.1603	0.1701	0.1021	0.7511
T6-F7	0.1860	0.1846	-0.0012	0.9731
T6-T3	0.1691	0.1629	-0.0481	0.8276
T6-T5	0.1352	0.1213	-0.1817	0.6724
T6-O1	0.1055	0.1094	0.0342	0.8543
T6-F4	0.2101	0.1953	-0.1697	0.6827
T6-C4	0.2252	0.1906	-1.0326	0.3160
T6-P4	0.1931	0.1717	-0.4029	0.5294
T6-F3	0.1960	0.1759	-0.3172	0.5766
T6-C3	0.2045	0.1749	-0.7702	0.3857
T6-P3	0.1647	0.1512	-0.2357	0.6301
T6-Fpz1	0.1606	0.1566	-0.0163	0.8992
T6-Fz	0.2006	0.1689	-0.7664	0.3868
T6-Cz	0.2466	0.1578	-7.2076	0.0107
T6-Pz	0.1921	0.1574	-1.2194	0.2764
T6-Oz	0.1591	0.1193	-1.2917	0.2629
O2-Fp2	0.1165	0.1315	0.3319	0.5679
O2-F8	0.1562	0.1306	-0.5575	0.4599
O2-T4	0.1167	0.1305	0.3693	0.5470
O2-T6	0.1321	0.1271	-0.0276	0.8689
O2-O2				
O2-Fp1	0.1187	0.1158	-0.0131	0.9093
O2-F7	0.1399	0.1231	-0.3050	0.5840
O2-T3	0.1377	0.1338	-0.0203	0.8873
O2-T5	0.1203	0.1220	0.0054	0.9418
O2-O1	0.1167	0.1144	-0.0081	0.9286
O2-F4	0.1325	0.1269	-0.0588	0.8098
O2-C4	0.1305	0.1248	-0.0493	0.8254
O2-P4	0.1492	0.1782	0.9239	0.3425
O2-F3	0.1396	0.1139	-1.0258	0.3176
O2-C3	0.1436	0.1169	-1.1223	0.2961
O2-P3	0.1525	0.1511	-0.0026	0.9597
O2-Fpz1	0.1274	0.1243	-0.0120	0.9134
O2-Fz	0.1347	0.1226	-0.2433	0.6246
O2-Cz	0.1592	0.1207	-1.7565	0.1930
O2-Pz	0.1778	0.1841	0.0337	0.8554
O2-Oz	0.1285	0.1466	0.2936	0.5911
Fp1-Fp2	0.0831	0.0901	0.1714	0.6812

Broadband-1	Mean WS	Mean TD	Signed F	p
Fp1-F8	0.1477	0.1204	-1.1181	0.2970
Fp1-T4	0.2203	0.1707	-2.3670	0.1322
Fp1-T6	0.1603	0.1701	0.1021	0.7511
Fp1-O2	0.1187	0.1158	-0.0131	0.9093
Fp1-Fp1				
Fp1-F7	0.1622	0.1537	-0.0858	0.7711
Fp1-T3	0.2426	0.2075	-0.7952	0.3781
Fp1-T5	0.1869	0.2013	0.1384	0.7119
Fp1-O1	0.1320	0.1215	-0.1735	0.6794
Fp1-F4	0.1113	0.0937	-0.8239	0.3698
Fp1-C4	0.1146	0.1098	-0.0432	0.8364
Fp1-P4	0.1231	0.1638	1.7570	0.1929
Fp1-F3	0.1593	0.1405	-0.3878	0.5372
Fp1-C3	0.1269	0.1166	-0.1564	0.6947
Fp1-P3	0.1387	0.1671	0.7064	0.4059
Fp1-Fpz1	0.1304	0.1342	0.0169	0.8971
Fp1-Fz	0.1509	0.1397	-0.1626	0.6891
Fp1-Cz	0.1269	0.0961	-1.7205	0.1975
Fp1-Pz	0.1173	0.1388	0.6967	0.4091
Fp1-Oz	0.1488	0.1023	-3.3746	0.0740
F7-Fp2	0.1164	0.1193	0.0140	0.9064
F7-F8	0.1459	0.1012	-2.0134	0.1641
F7-T4	0.2040	0.1581	-1.3823	0.2470
F7-T6	0.1860	0.1846	-0.0012	0.9731
F7-O2	0.1399	0.1231	-0.3050	0.5840
F7-Fp1	0.1622	0.1537	-0.0858	0.7711
F7-F7				
F7-T3	0.2381	0.2158	-0.2424	0.6253
F7-T5	0.2148	0.2079	-0.0186	0.8922
F7-O1	0.1458	0.1249	-0.4548	0.5042
F7-F4	0.1295	0.1094	-0.7732	0.3848
F7-C4	0.1418	0.1228	-0.3238	0.5727
F7-P4	0.1339	0.1709	1.5139	0.2261
F7-F3	0.2016	0.1572	-1.3002	0.2613
F7-C3	0.1427	0.1354	-0.0396	0.8433
F7-P3	0.1428	0.2022	2.1179	0.1538
F7-Fpz1	0.1378	0.1084	-1.1395	0.2925
F7-Fz	0.1879	0.1160	-7.0533	0.0115
F7-Cz	0.1575	0.1019	-6.8442	0.0127
F7-Pz	0.1251	0.1650	1.3432	0.2537
F7-Oz	0.1599	0.0898	-4.8038	0.0346
T3-Fp2	0.1767	0.1700	-0.0454	0.8324
T3-F8	0.1687	0.1483	-0.3302	0.5689
T3-T4	0.1716	0.1124	-3.8085	0.0584
T3-T6	0.1691	0.1629	-0.0481	0.8276
T3-O2	0.1377	0.1338	-0.0203	0.8873
T3-Fp1	0.2426	0.2075	-0.7952	0.3781
T3-F7	0.2381	0.2158	-0.2424	0.6253
T3-T3				
T3-T5	0.1996	0.1698	-0.4562	0.5035
T3-O1	0.1459	0.1473	0.0026	0.9599
T3-F4	0.1966	0.1709	-0.9663	0.3318
T3-C4	0.1653	0.1495	-0.3796	0.5415
T3-P4	0.1045	0.1438	2.6055	0.1148
T3-F3	0.3110	0.2164	-7.3292	0.0101
T3-C3	0.2201	0.1801	-1.5348	0.2230

Broadband-1	Mean WS	Mean TD	Signed F	p
T3-P3	0.1199	0.1645	2.1902	0.1471
T3-Fpz1	0.2096	0.1549	-2.4211	0.1280
T3-Fz	0.2830	0.1794	-10.2081	0.0028
T3-Cz	0.2415	0.1495	-12.7459	0.0010
T3-Pz	0.1277	0.1453	0.3613	0.5514
T3-Oz	0.1679	0.1260	-1.5738	0.2173
T5-Fp2	0.1593	0.1849	0.5920	0.4464
T5-F8	0.1989	0.1569	-0.9597	0.3335
T5-T4	0.1385	0.1069	-0.9640	0.3324
T5-T6	0.1352	0.1213	-0.1817	0.6724
T5-O2	0.1203	0.1220	0.0054	0.9418
T5-Fp1	0.1869	0.2013	0.1384	0.7119
T5-F7	0.2148	0.2079	-0.0186	0.8922
T5-T3	0.1996	0.1698	-0.4562	0.5035
T5-T5				
T5-O1	0.1542	0.1605	0.0451	0.8329
T5-F4	0.1732	0.1841	0.0962	0.7582
T5-C4	0.1799	0.1884	0.0755	0.7850
T5-P4	0.1298	0.1640	1.6067	0.2127
T5-F3	0.2396	0.2029	-0.7837	0.3816
T5-C3	0.2559	0.2268	-0.5469	0.4641
T5-P3	0.2139	0.2124	-0.0016	0.9679
T5-Fpz1	0.1680	0.1666	-0.0016	0.9684
T5-Fz	0.2260	0.1927	-0.7031	0.4070
T5-Cz	0.2603	0.1787	-5.3507	0.0262
T5-Pz	0.2044	0.1843	-0.3553	0.5546
T5-Oz	0.1669	0.1331	-0.6636	0.4204
O1-Fp2	0.1366	0.1261	-0.2026	0.6552
O1-F8	0.1628	0.1130	-2.9997	0.0914
O1-T4	0.1366	0.1076	-1.6660	0.2046
O1-T6	0.1055	0.1094	0.0342	0.8543
O1-O2	0.1167	0.1144	-0.0081	0.9286
O1-Fp1	0.1320	0.1215	-0.1735	0.6794
O1-F7	0.1458	0.1249	-0.4548	0.5042
O1-T3	0.1459	0.1473	0.0026	0.9599
O1-T5	0.1542	0.1605	0.0451	0.8329
O1-O1				
O1-F4	0.1456	0.1316	-0.3272	0.5707
O1-C4	0.1481	0.1302	-0.4501	0.5064
O1-P4	0.1362	0.1517	0.3021	0.5858
O1-F3	0.1581	0.1208	-1.6437	0.2076
O1-C3	0.1653	0.1398	-0.7381	0.3957
O1-P3	0.1829	0.1855	0.0066	0.9358
O1-Fpz1	0.1394	0.0994	-3.0683	0.0879
O1-Fz	0.1539	0.1062	-4.2287	0.0467
O1-Cz	0.1855	0.1123	-6.6174	0.0141
O1-Pz	0.2034	0.1930	-0.0837	0.7739
O1-Oz	0.1406	0.0927	-2.3875	0.1306
F4-Fp2	0.1501	0.1256	-0.6557	0.4231
F4-F8	0.2510	0.1134	-15.3964	0.0004
F4-T4	0.3298	0.1961	-14.6687	0.0005
F4-T6	0.2101	0.1953	-0.1697	0.6827
F4-O2	0.1325	0.1269	-0.0588	0.8098
F4-Fp1	0.1113	0.0937	-0.8239	0.3698
F4-F7	0.1295	0.1094	-0.7732	0.3848
F4-T3	0.1966	0.1709	-0.9663	0.3318

Broadband-1	Mean WS	Mean TD	Signed F	p
F4-T5	0.1732	0.1841	0.0962	0.7582
F4-O1	0.1456	0.1316	-0.3272	0.5707
F4-F4				
F4-C4	0.1792	0.1552	-0.5421	0.4661
F4-P4	0.1751	0.2110	1.1337	0.2937
F4-F3	0.1083	0.1055	-0.0095	0.9227
F4-C3	0.1246	0.1187	-0.0410	0.8407
F4-P3	0.1420	0.1822	1.4370	0.2381
F4-Fpz1	0.1467	0.1193	-0.8856	0.3526
F4-Fz	0.1496	0.1523	0.0071	0.9333
F4-Cz	0.1450	0.0913	-7.8314	0.0080
F4-Pz	0.1189	0.1726	4.0584	0.0511
F4-Oz	0.1554	0.1164	-2.2810	0.1392
C4-Fp2	0.1281	0.1240	-0.0239	0.8780
C4-F8	0.1663	0.1184	-1.3584	0.2511
C4-T4	0.2471	0.1455	-12.2071	0.0012
C4-T6	0.2252	0.1906	-1.0326	0.3160
C4-O2	0.1305	0.1248	-0.0493	0.8254
C4-Fp1	0.1146	0.1098	-0.0432	0.8364
C4-F7	0.1418	0.1228	-0.3238	0.5727
C4-T3	0.1653	0.1495	-0.3796	0.5415
C4-T5	0.1799	0.1884	0.0755	0.7850
C4-O1	0.1481	0.1302	-0.4501	0.5064
C4-F4	0.1792	0.1552	-0.5421	0.4661
C4-C4				
C4-P4	0.1952	0.2117	0.2141	0.6462
C4-F3	0.1663	0.1355	-0.7008	0.4077
C4-C3	0.0949	0.0872	-0.1067	0.7457
C4-P3	0.1405	0.1677	0.6828	0.4138
C4-Fpz1	0.1296	0.1144	-0.3746	0.5441
C4-Fz	0.1758	0.1426	-1.4498	0.2360
C4-Cz	0.2242	0.1275	-10.4498	0.0025
C4-Pz	0.1225	0.1596	1.3213	0.2575
C4-Oz	0.1444	0.0993	-2.8556	0.0992
P4-Fp2	0.1250	0.1592	1.4030	0.2436
P4-F8	0.1357	0.1766	2.4241	0.1278
P4-T4	0.1302	0.1193	-0.2339	0.6315
P4-T6	0.1931	0.1717	-0.4029	0.5294
P4-O2	0.1492	0.1782	0.9239	0.3425
P4-Fp1	0.1231	0.1638	1.7570	0.1929
P4-F7	0.1339	0.1709	1.5139	0.2261
P4-T3	0.1045	0.1438	2.6055	0.1148
P4-T5	0.1298	0.1640	1.6067	0.2127
P4-O1	0.1362	0.1517	0.3021	0.5858
P4-F4	0.1751	0.2110	1.1337	0.2937
P4-C4	0.1952	0.2117	0.2141	0.6462
P4-P4				
P4-F3	0.1716	0.1797	0.0605	0.8071
P4-C3	0.1819	0.1930	0.1130	0.7386
P4-P3	0.1074	0.1233	0.3800	0.5413
P4-Fpz1	0.1216	0.1471	0.7786	0.3831
P4-Fz	0.1726	0.1719	-0.0006	0.9800
P4-Cz	0.2796	0.1783	-7.6188	0.0088
P4-Pz	0.1878	0.1348	-3.8048	0.0585
P4-Oz	0.1543	0.1472	-0.0529	0.8192
F3-Fp2	0.1464	0.1164	-1.4472	0.2364

Broadband-1	Mean WS	Mean TD	Signed F	p
F3-F8	0.1915	0.1002	-7.9102	0.0077
F3-T4	0.2649	0.1761	-7.5305	0.0092
F3-T6	0.1960	0.1759	-0.3172	0.5766
F3-O2	0.1396	0.1139	-1.0258	0.3176
F3-Fp1	0.1593	0.1405	-0.3878	0.5372
F3-F7	0.2016	0.1572	-1.3002	0.2613
F3-T3	0.3110	0.2164	-7.3292	0.0101
F3-T5	0.2396	0.2029	-0.7837	0.3816
F3-O1	0.1581	0.1208	-1.6437	0.2076
F3-F4	0.1083	0.1055	-0.0095	0.9227
F3-C4	0.1663	0.1355	-0.7008	0.4077
F3-P4	0.1716	0.1797	0.0605	0.8071
F3-F3				
F3-C3	0.1855	0.1588	-0.3280	0.5702
F3-P3	0.1750	0.1854	0.0672	0.7968
F3-Fpz1	0.1713	0.1276	-2.2480	0.1421
F3-Fz	0.1371	0.1512	0.1524	0.6984
F3-Cz	0.1348	0.0852	-5.2795	0.0272
F3-Pz	0.1322	0.1510	0.3771	0.5428
F3-Oz	0.1692	0.1036	-4.1089	0.0497
C3-Fp2	0.1148	0.1233	0.1201	0.7308
C3-F8	0.1460	0.1140	-0.9161	0.3446
C3-T4	0.2059	0.1378	-6.7259	0.0134
C3-T6	0.2045	0.1749	-0.7702	0.3857
C3-O2	0.1436	0.1169	-1.1223	0.2961
C3-Fp1	0.1269	0.1166	-0.1564	0.6947
C3-F7	0.1427	0.1354	-0.0396	0.8433
C3-T3	0.2201	0.1801	-1.5348	0.2230
C3-T5	0.2559	0.2268	-0.5469	0.4641
C3-O1	0.1653	0.1398	-0.7381	0.3957
C3-F4	0.1246	0.1187	-0.0410	0.8407
C3-C4	0.0949	0.0872	-0.1067	0.7457
C3-P4	0.1819	0.1930	0.1130	0.7386
C3-F3	0.1855	0.1588	-0.3280	0.5702
C3-C3				
C3-P3	0.2114	0.1890	-0.3181	0.5761
C3-Fpz1	0.1342	0.1084	-0.9229	0.3428
C3-Fz	0.1738	0.1348	-1.3690	0.2493
C3-Cz	0.2151	0.1146	-8.2051	0.0068
C3-Pz	0.1385	0.1711	0.9940	0.3251
C3-Oz	0.1553	0.1118	-2.3650	0.1324
P3-Fp2	0.1290	0.1560	0.8919	0.3509
P3-F8	0.1271	0.1588	0.9532	0.3351
P3-T4	0.1306	0.0960	-2.1856	0.1475
P3-T6	0.1647	0.1512	-0.2357	0.6301
P3-O2	0.1525	0.1511	-0.0026	0.9597
P3-Fp1	0.1387	0.1671	0.7064	0.4059
P3-F7	0.1428	0.2022	2.1179	0.1538
P3-T3	0.1199	0.1645	2.1902	0.1471
P3-T5	0.2139	0.2124	-0.0016	0.9679
P3-O1	0.1829	0.1855	0.0066	0.9358
P3-F4	0.1420	0.1822	1.4370	0.2381
P3-C4	0.1405	0.1677	0.6828	0.4138
P3-P4	0.1074	0.1233	0.3800	0.5413
P3-F3	0.1750	0.1854	0.0672	0.7968
P3-C3	0.2114	0.1890	-0.3181	0.5761

Broadband-1	Mean WS	Mean TD	Signed F	p
P3-P3				
P3-Fpz1	0.1327	0.1268	-0.0450	0.8332
P3-Fz	0.1742	0.1571	-0.2869	0.5954
P3-Cz	0.2704	0.1675	-7.6281	0.0088
P3-Pz	0.1662	0.1459	-0.4349	0.5136
P3-Oz	0.1811	0.1537	-0.5637	0.4574
Fpz1-Fp2	0.1111	0.1101	-0.0022	0.9632
Fpz1-F8	0.1718	0.1026	-7.3571	0.0100
Fpz1-T4	0.2382	0.1730	-3.4788	0.0699
Fpz1-T6	0.1606	0.1566	-0.0163	0.8992
Fpz1-O2	0.1274	0.1243	-0.0120	0.9134
Fpz1-Fp1	0.1304	0.1342	0.0169	0.8971
Fpz1-F7	0.1378	0.1084	-1.1395	0.2925
Fpz1-T3	0.2096	0.1549	-2.4211	0.1280
Fpz1-T5	0.1680	0.1666	-0.0016	0.9684
Fpz1-O1	0.1394	0.0994	-3.0683	0.0879
Fpz1-F4	0.1467	0.1193	-0.8856	0.3526
Fpz1-C4	0.1296	0.1144	-0.3746	0.5441
Fpz1-P4	0.1216	0.1471	0.7786	0.3831
Fpz1-F3	0.1713	0.1276	-2.2480	0.1421
Fpz1-C3	0.1342	0.1084	-0.9229	0.3428
Fpz1-P3	0.1327	0.1268	-0.0450	0.8332
Fpz1-Fpz1				
Fpz1-Fz	0.1788	0.1277	-2.9341	0.0949
Fpz1-Cz	0.1381	0.0986	-2.4703	0.1243
Fpz1-Pz	0.1192	0.1238	0.0362	0.8501
Fpz1-Oz	0.1722	0.1071	-5.3543	0.0262
Fz-Fp2	0.1723	0.1247	-2.9051	0.0965
Fz-F8	0.1956	0.0981	-13.4252	0.0008
Fz-T4	0.2903	0.1856	-8.8430	0.0051
Fz-T6	0.2006	0.1689	-0.7664	0.3868
Fz-O2	0.1347	0.1226	-0.2433	0.6246
Fz-Fp1	0.1509	0.1397	-0.1626	0.6891
Fz-F7	0.1879	0.1160	-7.0533	0.0115
Fz-T3	0.2830	0.1794	-10.2081	0.0028
Fz-T5	0.2260	0.1927	-0.7031	0.4070
Fz-O1	0.1539	0.1062	-4.2287	0.0467
Fz-F4	0.1496	0.1523	0.0071	0.9333
Fz-C4	0.1758	0.1426	-1.4498	0.2360
Fz-P4	0.1726	0.1719	-0.0006	0.9800
Fz-F3	0.1371	0.1512	0.1524	0.6984
Fz-C3	0.1738	0.1348	-1.3690	0.2493
Fz-P3	0.1742	0.1571	-0.2869	0.5954
Fz-Fpz1	0.1788	0.1277	-2.9341	0.0949
Fz-Fz				
Fz-Cz	0.1478	0.1115	-2.7131	0.1078
Fz-Pz	0.1233	0.1370	0.2888	0.5941
Fz-Oz	0.1624	0.0923	-6.9821	0.0119
Cz-Fp2	0.1425	0.0968	-3.7454	0.0604
Cz-F8	0.1603	0.0865	-13.7054	0.0007
Cz-T4	0.2597	0.1308	-23.5717	0.0000
Cz-T6	0.2466	0.1578	-7.2076	0.0107
Cz-O2	0.1592	0.1207	-1.7565	0.1930
Cz-Fp1	0.1269	0.0961	-1.7205	0.1975
Cz-F7	0.1575	0.1019	-6.8442	0.0127
Cz-T3	0.2415	0.1495	-12.7459	0.0010

Broadband-1	Mean WS	Mean TD	Signed F	p
Cz-T5	0.2603	0.1787	-5.3507	0.0262
Cz-O1	0.1855	0.1123	-6.6174	0.0141
Cz-F4	0.1450	0.0913	-7.8314	0.0080
Cz-C4	0.2242	0.1275	-10.4498	0.0025
Cz-P4	0.2796	0.1783	-7.6188	0.0088
Cz-F3	0.1348	0.0852	-5.2795	0.0272
Cz-C3	0.2151	0.1146	-8.2051	0.0068
Cz-P3	0.2704	0.1675	-7.6281	0.0088
Cz-Fpz1	0.1381	0.0986	-2.4703	0.1243
Cz-Fz	0.1478	0.1115	-2.7131	0.1078
Cz-Cz				
Cz-Pz	0.2336	0.1518	-6.6671	0.0138
Cz-Oz	0.1768	0.0956	-7.7221	0.0084
Pz-Fp2	0.1181	0.1379	0.7500	0.3919
Pz-F8	0.1137	0.1484	1.7680	0.1916
Pz-T4	0.1418	0.0966	-3.7210	0.0612
Pz-T6	0.1921	0.1574	-1.2194	0.2764
Pz-O2	0.1778	0.1841	0.0337	0.8554
Pz-Fp1	0.1173	0.1388	0.6967	0.4091
Pz-F7	0.1251	0.1650	1.3432	0.2537
Pz-T3	0.1277	0.1453	0.3613	0.5514
Pz-T5	0.2044	0.1843	-0.3553	0.5546
Pz-O1	0.2034	0.1930	-0.0837	0.7739
Pz-F4	0.1189	0.1726	4.0584	0.0511
Pz-C4	0.1225	0.1596	1.3213	0.2575
Pz-P4	0.1878	0.1348	-3.8048	0.0585
Pz-F3	0.1322	0.1510	0.3771	0.5428
Pz-C3	0.1385	0.1711	0.9940	0.3251
Pz-P3	0.1662	0.1459	-0.4349	0.5136
Pz-Fpz1	0.1192	0.1238	0.0362	0.8501
Pz-Fz	0.1233	0.1370	0.2888	0.5941
Pz-Cz	0.2336	0.1518	-6.6671	0.0138
Pz-Pz				
Pz-Oz	0.2040	0.1875	-0.1676	0.6845
Oz-Fp2	0.1552	0.1097	-3.1475	0.0841
Oz-F8	0.1824	0.0991	-7.3896	0.0098
Oz-T4	0.1407	0.1087	-1.6301	0.2094
Oz-T6	0.1591	0.1193	-1.2917	0.2629
Oz-O2	0.1285	0.1466	0.2936	0.5911
Oz-Fp1	0.1488	0.1023	-3.3746	0.0740
Oz-F7	0.1599	0.0898	-4.8038	0.0346
Oz-T3	0.1679	0.1260	-1.5738	0.2173
Oz-T5	0.1669	0.1331	-0.6636	0.4204
Oz-O1	0.1406	0.0927	-2.3875	0.1306
Oz-F4	0.1554	0.1164	-2.2810	0.1392
Oz-C4	0.1444	0.0993	-2.8556	0.0992
Oz-P4	0.1543	0.1472	-0.0529	0.8192
Oz-F3	0.1692	0.1036	-4.1089	0.0497
Oz-C3	0.1553	0.1118	-2.3650	0.1324
Oz-P3	0.1811	0.1537	-0.5637	0.4574
Oz-Fpz1	0.1722	0.1071	-5.3543	0.0262
Oz-Fz	0.1624	0.0923	-6.9821	0.0119
Oz-Cz	0.1768	0.0956	-7.7221	0.0084
Oz-Pz	0.2040	0.1875	-0.1676	0.6845
Oz-Oz				

Supplementary Table S6. Group means, signed F-values and probabilities of region-specific NREM sleep EEG Broadband-2 (0.5-30 Hz) WPLI differences of Williams syndrome and typically developing subjects in all the possible electrode pairings. Color codes: Red = WS > TD (B-H corrected), Yellow = WS > TD (uncorrected), White = WS ≈ typically developing, Light blue = WS < TD (uncorrected), Blue = WS < TD (B-H corrected).

Broadband-2	Mean WS	Mean TD	Signed F	p
Fp2-Fp2				
Fp2-F8	0.0851	0.0885	0.2344	0.6310
Fp2-T4	0.0658	0.0683	0.1690	0.6833
Fp2-T6	0.0602	0.0788	5.4618	0.0248
Fp2-O2	0.0551	0.0680	3.0725	0.0877
Fp2-Fp1	0.0400	0.0492	1.3568	0.2514
Fp2-F7	0.0566	0.0600	0.2704	0.6061
Fp2-T3	0.0665	0.0739	0.3915	0.5353
Fp2-T5	0.0583	0.0676	2.1465	0.1511
Fp2-O1	0.0512	0.0616	3.9918	0.0529
Fp2-F4	0.1052	0.1242	6.1255	0.0179
Fp2-C4	0.0726	0.0939	8.0164	0.0074
Fp2-P4	0.0568	0.0704	3.1200	0.0854
Fp2-F3	0.0699	0.0826	3.8819	0.0561
Fp2-C3	0.0665	0.0805	3.4077	0.0727
Fp2-P3	0.0566	0.0636	0.8623	0.3590
Fp2-Fpz1	0.0853	0.0751	-1.1694	0.2863
Fp2-Fz	0.0877	0.0957	0.4144	0.5236
Fp2-Cz	0.0769	0.0891	1.7472	0.1941
Fp2-Pz	0.0537	0.0712	5.0613	0.0303
Fp2-Oz	0.0545	0.0654	3.1995	0.0816
F8-Fp2	0.0851	0.0885	0.2344	0.6310
F8-F8				
F8-T4	0.0741	0.0662	-0.8078	0.3744
F8-T6	0.0664	0.0771	2.2002	0.1462
F8-O2	0.0538	0.0671	4.2756	0.0455
F8-Fp1	0.0580	0.0721	4.1532	0.0486
F8-F7	0.0588	0.0598	0.0050	0.9439
F8-T3	0.0568	0.0699	2.3514	0.1335
F8-T5	0.0548	0.0635	2.1307	0.1526
F8-O1	0.0480	0.0579	2.9533	0.0938
F8-F4	0.0678	0.0837	2.5566	0.1181
F8-C4	0.0623	0.0831	6.5826	0.0144
F8-P4	0.0534	0.0695	5.5841	0.0233
F8-F3	0.0463	0.0592	5.1691	0.0287
F8-C3	0.0483	0.0633	5.6507	0.0226
F8-P3	0.0454	0.0557	3.7024	0.0618
F8-Fpz1	0.0818	0.0910	1.4314	0.2390
F8-Fz	0.0540	0.0607	1.2233	0.2757
F8-Cz	0.0528	0.0734	8.4733	0.0060
F8-Pz	0.0435	0.0597	8.8955	0.0050
F8-Oz	0.0545	0.0647	2.6480	0.1119
T4-Fp2	0.0658	0.0683	0.1690	0.6833
T4-F8	0.0741	0.0662	-0.8078	0.3744
T4-T4				
T4-T6	0.0723	0.0712	-0.0082	0.9281
T4-O2	0.0608	0.0646	0.2095	0.6497
T4-Fp1	0.0679	0.0748	0.3429	0.5616
T4-F7	0.0677	0.0658	-0.0255	0.8740
T4-T3	0.0642	0.0646	0.0005	0.9817

Broadband-2	Mean WS	Mean TD	Signed F	p
T4-T5	0.0525	0.0556	0.1898	0.6655
T4-O1	0.0518	0.0498	-0.1055	0.7471
T4-F4	0.0732	0.0650	-1.2411	0.2722
T4-C4	0.0952	0.0755	-2.7307	0.1067
T4-P4	0.0709	0.0622	-0.4442	0.5091
T4-F3	0.0599	0.0600	0.0002	0.9889
T4-C3	0.0580	0.0603	0.0537	0.8179
T4-P3	0.0474	0.0483	0.0145	0.9047
T4-Fpz1	0.0754	0.0783	0.0910	0.7646
T4-Fz	0.0639	0.0611	-0.1576	0.6936
T4-Cz	0.0600	0.0652	0.6955	0.4095
T4-Pz	0.0485	0.0585	1.6762	0.2032
T4-Oz	0.0619	0.0607	-0.0176	0.8951
T6-Fp2	0.0602	0.0788	5.4618	0.0248
T6-F8	0.0664	0.0771	2.2002	0.1462
T6-T4	0.0723	0.0712	-0.0082	0.9281
T6-T6				
T6-O2	0.0687	0.0705	0.0273	0.8697
T6-Fp1	0.0694	0.0774	0.6331	0.4312
T6-F7	0.0627	0.0803	4.6283	0.0379
T6-T3	0.0677	0.0626	-0.0983	0.7556
T6-T5	0.0498	0.0566	0.4643	0.4998
T6-O1	0.0508	0.0528	0.0539	0.8177
T6-F4	0.0748	0.0791	0.3137	0.5787
T6-C4	0.0731	0.0796	1.0039	0.3227
T6-P4	0.0719	0.0623	-0.5600	0.4589
T6-F3	0.0734	0.0744	0.0149	0.9034
T6-C3	0.0640	0.0661	0.0614	0.8056
T6-P3	0.0555	0.0546	-0.0066	0.9356
T6-Fpz1	0.0732	0.0836	1.2881	0.2635
T6-Fz	0.0789	0.0821	0.0698	0.7931
T6-Cz	0.0800	0.0816	0.0150	0.9033
T6-Pz	0.0585	0.0644	0.3520	0.5565
T6-Oz	0.0641	0.0670	0.0899	0.7660
O2-Fp2	0.0551	0.0680	3.0725	0.0877
O2-F8	0.0538	0.0671	4.2756	0.0455
O2-T4	0.0608	0.0646	0.2095	0.6497
O2-T6	0.0687	0.0705	0.0273	0.8697
O2-O2				
O2-Fp1	0.0528	0.0711	6.3096	0.0164
O2-F7	0.0547	0.0638	1.4122	0.2421
O2-T3	0.0464	0.0615	5.1289	0.0293
O2-T5	0.0453	0.0516	1.2403	0.2724
O2-O1	0.0467	0.0443	-0.0920	0.7633
O2-F4	0.0504	0.0643	6.6614	0.0138
O2-C4	0.0546	0.0664	2.6558	0.1114
O2-P4	0.0535	0.0623	1.7835	0.1897
O2-F3	0.0505	0.0658	6.6705	0.0138
O2-C3	0.0453	0.0624	11.7078	0.0015
O2-P3	0.0441	0.0531	3.2618	0.0788
O2-Fpz1	0.0566	0.0744	5.6593	0.0225
O2-Fz	0.0546	0.0676	3.2086	0.0812
O2-Cz	0.0573	0.0667	0.8900	0.3514
O2-Pz	0.0505	0.0587	2.0886	0.1566
O2-Oz	0.0590	0.0563	-0.1805	0.6733
Fp1-Fp2	0.0400	0.0492	1.3568	0.2514

Broadband-2	Mean WS	Mean TD	Signed F	p
Fp1-F8	0.0580	0.0721	4.1532	0.0486
Fp1-T4	0.0679	0.0748	0.3429	0.5616
Fp1-T6	0.0694	0.0774	0.6331	0.4312
Fp1-O2	0.0528	0.0711	6.3096	0.0164
Fp1-Fp1				
Fp1-F7	0.0785	0.0898	1.2328	0.2738
Fp1-T3	0.0593	0.0696	2.6464	0.1120
Fp1-T5	0.0554	0.0715	3.6773	0.0627
Fp1-O1	0.0554	0.0662	1.0816	0.3049
Fp1-F4	0.0767	0.0984	6.1328	0.0178
Fp1-C4	0.0699	0.0877	3.8260	0.0578
Fp1-P4	0.0524	0.0731	9.1385	0.0045
Fp1-F3	0.1025	0.1216	4.8471	0.0338
Fp1-C3	0.0719	0.0903	6.7142	0.0135
Fp1-P3	0.0575	0.0705	2.2614	0.1409
Fp1-Fpz1	0.0731	0.0799	0.3212	0.5742
Fp1-Fz	0.0897	0.1060	1.3817	0.2471
Fp1-Cz	0.0813	0.0961	1.1323	0.2940
Fp1-Pz	0.0557	0.0755	4.3646	0.0434
Fp1-Oz	0.0509	0.0670	4.8940	0.0330
F7-Fp2	0.0566	0.0600	0.2704	0.6061
F7-F8	0.0588	0.0598	0.0050	0.9439
F7-T4	0.0677	0.0658	-0.0255	0.8740
F7-T6	0.0627	0.0803	4.6283	0.0379
F7-O2	0.0547	0.0638	1.4122	0.2421
F7-Fp1	0.0785	0.0898	1.2328	0.2738
F7-F7				
F7-T3	0.0656	0.0688	0.0926	0.7626
F7-T5	0.0658	0.0672	0.0201	0.8880
F7-O1	0.0583	0.0616	0.0861	0.7708
F7-F4	0.0593	0.0676	0.4061	0.5278
F7-C4	0.0598	0.0737	0.9752	0.3296
F7-P4	0.0591	0.0689	0.5923	0.4463
F7-F3	0.0838	0.0933	0.3107	0.5805
F7-C3	0.0755	0.0855	0.4370	0.5126
F7-P3	0.0605	0.0641	0.0764	0.7837
F7-Fpz1	0.0837	0.0790	-0.1048	0.7480
F7-Fz	0.0655	0.0690	0.0842	0.7733
F7-Cz	0.0716	0.0779	0.1618	0.6898
F7-Pz	0.0584	0.0687	0.5215	0.4746
F7-Oz	0.0583	0.0635	0.2043	0.6539
T3-Fp2	0.0665	0.0739	0.3915	0.5353
T3-F8	0.0568	0.0699	2.3514	0.1335
T3-T4	0.0642	0.0646	0.0005	0.9817
T3-T6	0.0677	0.0626	-0.0983	0.7556
T3-O2	0.0464	0.0615	5.1289	0.0293
T3-Fp1	0.0593	0.0696	2.6464	0.1120
T3-F7	0.0656	0.0688	0.0926	0.7626
T3-T3				
T3-T5	0.0622	0.0737	1.1265	0.2952
T3-O1	0.0624	0.0710	0.2784	0.6008
T3-F4	0.0581	0.0721	2.5244	0.1204
T3-C4	0.0647	0.0711	0.2559	0.6159
T3-P4	0.0542	0.0589	0.1816	0.6724
T3-F3	0.0707	0.0792	1.1643	0.2874
T3-C3	0.0907	0.0905	-0.0005	0.9818

Broadband-2	Mean WS	Mean TD	Signed F	p
T3-P3	0.0615	0.0709	0.7847	0.3813
T3-Fpz1	0.0708	0.0795	0.6353	0.4304
T3-Fz	0.0719	0.0768	0.0976	0.7564
T3-Cz	0.0820	0.0822	0.0001	0.9928
T3-Pz	0.0565	0.0683	0.9747	0.3297
T3-Oz	0.0544	0.0660	1.2498	0.2706
T5-Fp2	0.0583	0.0676	2.1465	0.1511
T5-F8	0.0548	0.0635	2.1307	0.1526
T5-T4	0.0525	0.0556	0.1898	0.6655
T5-T6	0.0498	0.0566	0.4643	0.4998
T5-O2	0.0453	0.0516	1.2403	0.2724
T5-Fp1	0.0554	0.0715	3.6773	0.0627
T5-F7	0.0658	0.0672	0.0201	0.8880
T5-T3	0.0622	0.0737	1.1265	0.2952
T5-T5				
T5-O1	0.0580	0.0618	0.2209	0.6411
T5-F4	0.0519	0.0649	6.8419	0.0127
T5-C4	0.0549	0.0719	4.1930	0.0475
T5-P4	0.0496	0.0565	0.8513	0.3620
T5-F3	0.0638	0.0807	5.8390	0.0206
T5-C3	0.0738	0.0954	6.2564	0.0168
T5-P3	0.0696	0.0772	1.7265	0.1967
T5-Fpz1	0.0612	0.0701	1.7895	0.1889
T5-Fz	0.0607	0.0686	1.2650	0.2678
T5-Cz	0.0700	0.0825	1.4876	0.2301
T5-Pz	0.0580	0.0695	3.0254	0.0901
T5-Oz	0.0557	0.0632	1.4012	0.2439
O1-Fp2	0.0512	0.0616	3.9918	0.0529
O1-F8	0.0480	0.0579	2.9533	0.0938
O1-T4	0.0518	0.0498	-0.1055	0.7471
O1-T6	0.0508	0.0528	0.0539	0.8177
O1-O2	0.0467	0.0443	-0.0920	0.7633
O1-Fp1	0.0554	0.0662	1.0816	0.3049
O1-F7	0.0583	0.0616	0.0861	0.7708
O1-T3	0.0624	0.0710	0.2784	0.6008
O1-T5	0.0580	0.0618	0.2209	0.6411
O1-O1				
O1-F4	0.0538	0.0669	4.9857	0.0315
O1-C4	0.0504	0.0654	6.4520	0.0153
O1-P4	0.0527	0.0521	-0.0033	0.9543
O1-F3	0.0558	0.0718	4.6093	0.0382
O1-C3	0.0571	0.0769	4.2976	0.0450
O1-P3	0.0569	0.0607	0.1320	0.7184
O1-Fpz1	0.0562	0.0653	1.5186	0.2254
O1-Fz	0.0589	0.0715	1.8280	0.1844
O1-Cz	0.0609	0.0748	2.5411	0.1192
O1-Pz	0.0532	0.0637	1.9637	0.1692
O1-Oz	0.0582	0.0609	0.0784	0.7810
F4-Fp2	0.1052	0.1242	6.1255	0.0179
F4-F8	0.0678	0.0837	2.5566	0.1181
F4-T4	0.0732	0.0650	-1.2411	0.2722
F4-T6	0.0748	0.0791	0.3137	0.5787
F4-O2	0.0504	0.0643	6.6614	0.0138
F4-Fp1	0.0767	0.0984	6.1328	0.0178
F4-F7	0.0593	0.0676	0.4061	0.5278
F4-T3	0.0581	0.0721	2.5244	0.1204

Broadband-2	Mean WS	Mean TD	Signed F	p
F4-T5	0.0519	0.0649	6.8419	0.0127
F4-O1	0.0538	0.0669	4.9857	0.0315
F4-F4				
F4-C4	0.0552	0.0757	10.6993	0.0023
F4-P4	0.0602	0.0792	8.2460	0.0066
F4-F3	0.0397	0.0491	3.0167	0.0905
F4-C3	0.0466	0.0625	6.8265	0.0128
F4-P3	0.0490	0.0609	5.4006	0.0256
F4-Fpz1	0.1038	0.1256	5.9315	0.0197
F4-Fz	0.0555	0.0674	2.3126	0.1366
F4-Cz	0.0537	0.0779	11.2689	0.0018
F4-Pz	0.0471	0.0661	11.2193	0.0018
F4-Oz	0.0571	0.0708	4.2242	0.0468
C4-Fp2	0.0726	0.0939	8.0164	0.0074
C4-F8	0.0623	0.0831	6.5826	0.0144
C4-T4	0.0952	0.0755	-2.7307	0.1067
C4-T6	0.0731	0.0796	1.0039	0.3227
C4-O2	0.0546	0.0664	2.6558	0.1114
C4-Fp1	0.0699	0.0877	3.8260	0.0578
C4-F7	0.0598	0.0737	0.9752	0.3296
C4-T3	0.0647	0.0711	0.2559	0.6159
C4-T5	0.0549	0.0719	4.1930	0.0475
C4-O1	0.0504	0.0654	6.4520	0.0153
C4-F4	0.0552	0.0757	10.6993	0.0023
C4-C4				
C4-P4	0.0722	0.0826	1.2161	0.2771
C4-F3	0.0507	0.0642	4.8837	0.0332
C4-C3	0.0349	0.0447	2.6068	0.1147
C4-P3	0.0517	0.0630	1.8194	0.1854
C4-Fpz1	0.0777	0.0978	6.2968	0.0165
C4-Fz	0.0635	0.0886	5.1090	0.0296
C4-Cz	0.0511	0.0569	0.3668	0.5484
C4-Pz	0.0564	0.0728	3.4017	0.0729
C4-Oz	0.0563	0.0699	3.0582	0.0884
P4-Fp2	0.0568	0.0704	3.1200	0.0854
P4-F8	0.0534	0.0695	5.5841	0.0233
P4-T4	0.0709	0.0622	-0.4442	0.5091
P4-T6	0.0719	0.0623	-0.5600	0.4589
P4-O2	0.0535	0.0623	1.7835	0.1897
P4-Fp1	0.0524	0.0731	9.1385	0.0045
P4-F7	0.0591	0.0689	0.5923	0.4463
P4-T3	0.0542	0.0589	0.1816	0.6724
P4-T5	0.0496	0.0565	0.8513	0.3620
P4-O1	0.0527	0.0521	-0.0033	0.9543
P4-F4	0.0602	0.0792	8.2460	0.0066
P4-C4	0.0722	0.0826	1.2161	0.2771
P4-P4				
P4-F3	0.0542	0.0709	8.8595	0.0051
P4-C3	0.0495	0.0660	12.6050	0.0010
P4-P3	0.0405	0.0431	0.0937	0.7612
P4-Fpz1	0.0574	0.0770	8.2233	0.0067
P4-Fz	0.0677	0.0809	1.0503	0.3119
P4-Cz	0.0804	0.0878	0.1949	0.6613
P4-Pz	0.0465	0.0569	0.8538	0.3613
P4-Oz	0.0506	0.0660	4.9127	0.0327
F3-Fp2	0.0699	0.0826	3.8819	0.0561

Broadband-2	Mean WS	Mean TD	Signed F	p
F3-F8	0.0463	0.0592	5.1691	0.0287
F3-T4	0.0599	0.0600	0.0002	0.9889
F3-T6	0.0734	0.0744	0.0149	0.9034
F3-O2	0.0505	0.0658	6.6705	0.0138
F3-Fp1	0.1025	0.1216	4.8471	0.0338
F3-F7	0.0838	0.0933	0.3107	0.5805
F3-T3	0.0707	0.0792	1.1643	0.2874
F3-T5	0.0638	0.0807	5.8390	0.0206
F3-O1	0.0558	0.0718	4.6093	0.0382
F3-F4	0.0397	0.0491	3.0167	0.0905
F3-C4	0.0507	0.0642	4.8837	0.0332
F3-P4	0.0542	0.0709	8.8595	0.0051
F3-F3				
F3-C3	0.0568	0.0785	9.3996	0.0040
F3-P3	0.0586	0.0715	4.6793	0.0369
F3-Fpz1	0.0979	0.1132	3.6976	0.0620
F3-Fz	0.0605	0.0678	0.4687	0.4977
F3-Cz	0.0659	0.0808	2.0105	0.1644
F3-Pz	0.0542	0.0720	7.0477	0.0115
F3-Oz	0.0549	0.0709	5.1621	0.0288
C3-Fp2	0.0665	0.0805	3.4077	0.0727
C3-F8	0.0483	0.0633	5.6507	0.0226
C3-T4	0.0580	0.0603	0.0537	0.8179
C3-T6	0.0640	0.0661	0.0614	0.8056
C3-O2	0.0453	0.0624	11.7078	0.0015
C3-Fp1	0.0719	0.0903	6.7142	0.0135
C3-F7	0.0755	0.0855	0.4370	0.5126
C3-T3	0.0907	0.0905	-0.0005	0.9818
C3-T5	0.0738	0.0954	6.2564	0.0168
C3-O1	0.0571	0.0769	4.2976	0.0450
C3-F4	0.0466	0.0625	6.8265	0.0128
C3-C4	0.0349	0.0447	2.6068	0.1147
C3-P4	0.0495	0.0660	12.6050	0.0010
C3-F3	0.0568	0.0785	9.3996	0.0040
C3-C3				
C3-P3	0.0702	0.0892	5.2820	0.0271
C3-Fpz1	0.0746	0.0934	6.3269	0.0162
C3-Fz	0.0654	0.0862	2.8714	0.0983
C3-Cz	0.0536	0.0605	0.2878	0.5948
C3-Pz	0.0594	0.0774	3.9938	0.0529
C3-Oz	0.0512	0.0707	7.6581	0.0087
P3-Fp2	0.0566	0.0636	0.8623	0.3590
P3-F8	0.0454	0.0557	3.7024	0.0618
P3-T4	0.0474	0.0483	0.0145	0.9047
P3-T6	0.0555	0.0546	-0.0066	0.9356
P3-O2	0.0441	0.0531	3.2618	0.0788
P3-Fp1	0.0575	0.0705	2.2614	0.1409
P3-F7	0.0605	0.0641	0.0764	0.7837
P3-T3	0.0615	0.0709	0.7847	0.3813
P3-T5	0.0696	0.0772	1.7265	0.1967
P3-O1	0.0569	0.0607	0.1320	0.7184
P3-F4	0.0490	0.0609	5.4006	0.0256
P3-C4	0.0517	0.0630	1.8194	0.1854
P3-P4	0.0405	0.0431	0.0937	0.7612
P3-F3	0.0586	0.0715	4.6793	0.0369
P3-C3	0.0702	0.0892	5.2820	0.0271

Broadband-2	Mean WS	Mean TD	Signed F	p
P3-P3				
P3-Fpz1	0.0615	0.0707	1.2739	0.2661
P3-Fz	0.0588	0.0673	1.0973	0.3015
P3-Cz	0.0753	0.0840	0.5262	0.4727
P3-Pz	0.0415	0.0563	5.1343	0.0292
P3-Oz	0.0488	0.0631	3.0436	0.0891
Fpz1-Fp2	0.0853	0.0751	-1.1694	0.2863
Fpz1-F8	0.0818	0.0910	1.4314	0.2390
Fpz1-T4	0.0754	0.0783	0.0910	0.7646
Fpz1-T6	0.0732	0.0836	1.2881	0.2635
Fpz1-O2	0.0566	0.0744	5.6593	0.0225
Fpz1-Fp1	0.0731	0.0799	0.3212	0.5742
Fpz1-F7	0.0837	0.0790	-0.1048	0.7480
Fpz1-T3	0.0708	0.0795	0.6353	0.4304
Fpz1-T5	0.0612	0.0701	1.7895	0.1889
Fpz1-O1	0.0562	0.0653	1.5186	0.2254
Fpz1-F4	0.1038	0.1256	5.9315	0.0197
Fpz1-C4	0.0777	0.0978	6.2968	0.0165
Fpz1-P4	0.0574	0.0770	8.2233	0.0067
Fpz1-F3	0.0979	0.1132	3.6976	0.0620
Fpz1-C3	0.0746	0.0934	6.3269	0.0162
Fpz1-P3	0.0615	0.0707	1.2739	0.2661
Fpz1-Fpz1				
Fpz1-Fz	0.1079	0.1269	2.4192	0.1281
Fpz1-Cz	0.0866	0.1006	1.3129	0.2590
Fpz1-Pz	0.0588	0.0790	5.2713	0.0273
Fpz1-Oz	0.0537	0.0696	5.8563	0.0204
Fz-Fp2	0.0877	0.0957	0.4144	0.5236
Fz-F8	0.0540	0.0607	1.2233	0.2757
Fz-T4	0.0639	0.0611	-0.1576	0.6936
Fz-T6	0.0789	0.0821	0.0698	0.7931
Fz-O2	0.0546	0.0676	3.2086	0.0812
Fz-Fp1	0.0897	0.1060	1.3817	0.2471
Fz-F7	0.0655	0.0690	0.0842	0.7733
Fz-T3	0.0719	0.0768	0.0976	0.7564
Fz-T5	0.0607	0.0686	1.2650	0.2678
Fz-O1	0.0589	0.0715	1.8280	0.1844
Fz-F4	0.0555	0.0674	2.3126	0.1366
Fz-C4	0.0635	0.0886	5.1090	0.0296
Fz-P4	0.0677	0.0809	1.0503	0.3119
Fz-F3	0.0605	0.0678	0.4687	0.4977
Fz-C3	0.0654	0.0862	2.8714	0.0983
Fz-P3	0.0588	0.0673	1.0973	0.3015
Fz-Fpz1	0.1079	0.1269	2.4192	0.1281
Fz-Fz				
Fz-Cz	0.0749	0.1098	8.6559	0.0055
Fz-Pz	0.0509	0.0713	10.3031	0.0027
Fz-Oz	0.0623	0.0753	1.6668	0.2045
Cz-Fp2	0.0769	0.0891	1.7472	0.1941
Cz-F8	0.0528	0.0734	8.4733	0.0060
Cz-T4	0.0600	0.0652	0.6955	0.4095
Cz-T6	0.0800	0.0816	0.0150	0.9033
Cz-O2	0.0573	0.0667	0.8900	0.3514
Cz-Fp1	0.0813	0.0961	1.1323	0.2940
Cz-F7	0.0716	0.0779	0.1618	0.6898
Cz-T3	0.0820	0.0822	0.0001	0.9928

Broadband-2	Mean WS	Mean TD	Signed F	p
Cz-T5	0.0700	0.0825	1.4876	0.2301
Cz-O1	0.0609	0.0748	2.5411	0.1192
Cz-F4	0.0537	0.0779	11.2689	0.0018
Cz-C4	0.0511	0.0569	0.3668	0.5484
Cz-P4	0.0804	0.0878	0.1949	0.6613
Cz-F3	0.0659	0.0808	2.0105	0.1644
Cz-C3	0.0536	0.0605	0.2878	0.5948
Cz-P3	0.0753	0.0840	0.5262	0.4727
Cz-Fpz1	0.0866	0.1006	1.3129	0.2590
Cz-Fz	0.0749	0.1098	8.6559	0.0055
Cz-Cz				
Cz-Pz	0.0712	0.0933	5.8177	0.0208
Cz-Oz	0.0662	0.0746	0.4748	0.4950
Pz-Fp2	0.0537	0.0712	5.0613	0.0303
Pz-F8	0.0435	0.0597	8.8955	0.0050
Pz-T4	0.0485	0.0585	1.6762	0.2032
Pz-T6	0.0585	0.0644	0.3520	0.5565
Pz-O2	0.0505	0.0587	2.0886	0.1566
Pz-Fp1	0.0557	0.0755	4.3646	0.0434
Pz-F7	0.0584	0.0687	0.5215	0.4746
Pz-T3	0.0565	0.0683	0.9747	0.3297
Pz-T5	0.0580	0.0695	3.0254	0.0901
Pz-O1	0.0532	0.0637	1.9637	0.1692
Pz-F4	0.0471	0.0661	11.2193	0.0018
Pz-C4	0.0564	0.0728	3.4017	0.0729
Pz-P4	0.0465	0.0569	0.8538	0.3613
Pz-F3	0.0542	0.0720	7.0477	0.0115
Pz-C3	0.0594	0.0774	3.9938	0.0529
Pz-P3	0.0415	0.0563	5.1343	0.0292
Pz-Fpz1	0.0588	0.0790	5.2713	0.0273
Pz-Fz	0.0509	0.0713	10.3031	0.0027
Pz-Cz	0.0712	0.0933	5.8177	0.0208
Pz-Pz				
Pz-Oz	0.0529	0.0668	2.3803	0.1312
Oz-Fp2	0.0545	0.0654	3.1995	0.0816
Oz-F8	0.0545	0.0647	2.6480	0.1119
Oz-T4	0.0619	0.0607	-0.0176	0.8951
Oz-T6	0.0641	0.0670	0.0899	0.7660
Oz-O2	0.0590	0.0563	-0.1805	0.6733
Oz-Fp1	0.0509	0.0670	4.8940	0.0330
Oz-F7	0.0583	0.0635	0.2043	0.6539
Oz-T3	0.0544	0.0660	1.2498	0.2706
Oz-T5	0.0557	0.0632	1.4012	0.2439
Oz-O1	0.0582	0.0609	0.0784	0.7810
Oz-F4	0.0571	0.0708	4.2242	0.0468
Oz-C4	0.0563	0.0699	3.0582	0.0884
Oz-P4	0.0506	0.0660	4.9127	0.0327
Oz-F3	0.0549	0.0709	5.1621	0.0288
Oz-C3	0.0512	0.0707	7.6581	0.0087
Oz-P3	0.0488	0.0631	3.0436	0.0891
Oz-Fpz1	0.0537	0.0696	5.8563	0.0204
Oz-Fz	0.0623	0.0753	1.6668	0.2045
Oz-Cz	0.0662	0.0746	0.4748	0.4950
Oz-Pz	0.0529	0.0668	2.3803	0.1312
Oz-Oz				

Supplementary Table S7. Signed F-values and probabilities of region-specific and band-limited NREM sleep EEG WPLI differences between Williams syndrome and typically developing subjects (intra- and inter-regional pairings). Color codes: Red = WS > TD (B-H corrected), Yellow = WS > TD (uncorrected), White = WS ≈ typically developing, Light blue = WS < TD (uncorrected), Blue = WS < TD (B-H corrected).

NREM		PF/AC		LPF		C		T		P/IP		O	
		Sign. F	p	Sign. F	p	Sign. F	p	Sign. F	p	Sign. F	p	Sign. F	p
Slow oscillation	PF/AC	-0.957	0.334	-2.647	0.112	-1.390	0.246	-4.225	0.047	0.134	0.717	-1.684	0.202
	LPF	-2.647	0.112	-6.038	0.019	-2.056	0.160	-4.336	0.044	0.263	0.611	-2.417	0.128
	C	-1.390	0.246	-2.056	0.160	-7.158	0.011	-12.939	0.001	-1.117	0.297	-3.004	0.091
	T	-4.225	0.047	-4.336	0.044	-12.939	0.001	-3.808	0.058	-0.028	0.867	-0.512	0.479
	P/IP	0.134	0.717	0.263	0.611	-1.117	0.297	-0.028	0.867	-0.352	0.556	-0.047	0.829
	O	-1.684	0.202	-2.417	0.128	-3.004	0.091	-0.512	0.479	-0.047	0.829	-0.218	0.643
Delta	PF/AC	1.526	0.224	3.590	0.066	3.441	0.071	0.778	0.383	0.910	0.346	-0.739	0.395
	LPF	3.590	0.066	0.079	0.780	0.954	0.335	0.158	0.693	1.082	0.305	-0.673	0.417
	C	3.441	0.071	0.954	0.335	0.000	0.995	0.203	0.655	3.789	0.059	2.644	0.112
	T	0.778	0.383	0.158	0.693	0.203	0.655	0.642	0.428	0.684	0.414	0.112	0.739
	P/IP	0.910	0.346	1.082	0.305	3.789	0.059	0.684	0.414	3.402	0.073	3.477	0.070
	O	-0.739	0.395	-0.673	0.417	2.644	0.112	0.112	0.739	3.477	0.070	-5.372	0.026
Theta	PF/AC	5.679	0.022	8.127	0.007	8.870	0.005	2.150	0.151	9.349	0.004	1.948	0.171
	LPF	8.127	0.007	8.117	0.007	9.464	0.004	2.880	0.098	8.657	0.006	1.970	0.169
	C	8.870	0.005	9.464	0.004	10.274	0.003	6.408	0.016	13.852	0.001	4.600	0.038
	T	2.150	0.151	2.880	0.098	6.408	0.016	4.943	0.032	0.142	0.708	-0.354	0.555
	P/IP	9.349	0.004	8.657	0.006	13.852	0.001	0.142	0.708	1.556	0.220	-3.836	0.058
	O	1.948	0.171	1.970	0.169	4.600	0.038	-0.354	0.555	-3.836	0.058	-16.582	0.000
Alpha	PF/AC	-4.702	0.036	-2.143	0.151	-0.551	0.462	-1.029	0.317	-6.783	0.013	-1.466	0.233
	LPF	-2.143	0.151	-1.390	0.246	-1.599	0.214	-12.356	0.001	-6.634	0.014	-0.726	0.399
	C	-0.551	0.462	-1.599	0.214	-3.862	0.057	-9.568	0.004	-4.572	0.039	-1.109	0.299
	T	-1.029	0.317	-12.356	0.001	-9.568	0.004	2.150	0.151	-1.826	0.185	-0.040	0.843
	P/IP	-6.783	0.013	-6.634	0.014	-4.572	0.039	-1.826	0.185	-6.518	0.015	-3.234	0.080
	O	-1.466	0.233	-0.726	0.399	-1.109	0.299	-0.040	0.843	-3.234	0.080	-1.576	0.217
Low sigma	PF/AC	-4.551	0.039	-2.000	0.165	-1.728	0.197	-0.914	0.345	-2.694	0.109	-0.865	0.358
	LPF	-2.000	0.165	1.584	0.216	-0.622	0.435	-6.348	0.016	-1.531	0.224	-0.709	0.405
	C	-1.728	0.197	-0.622	0.435	-0.597	0.444	-7.997	0.007	-1.472	0.233	-0.792	0.379
	T	-0.914	0.345	-6.348	0.016	-7.997	0.007	5.238	0.028	0.184	0.670	0.400	0.531
	P/IP	-2.694	0.109	-1.531	0.224	-1.472	0.233	0.184	0.670	0.474	0.495	0.177	0.676
	O	-0.865	0.358	-0.709	0.405	-0.792	0.379	0.400	0.531	0.177	0.676	2.189	0.147
High sigma	PF/AC	5.214	0.028	9.527	0.004	6.568	0.014	6.734	0.013	2.945	0.094	3.516	0.068
	LPF	9.527	0.004	15.910	0.000	6.904	0.012	3.320	0.076	3.572	0.066	3.119	0.085
	C	6.568	0.014	6.904	0.012	7.003	0.012	1.582	0.216	7.781	0.008	5.985	0.019
	T	6.734	0.013	3.320	0.076	1.582	0.216	4.817	0.034	1.396	0.245	1.322	0.257
	P/IP	2.945	0.094	3.572	0.066	7.781	0.008	1.396	0.245	3.297	0.077	4.506	0.040
	O	3.516	0.068	3.119	0.085	5.985	0.019	1.322	0.257	4.506	0.040	5.857	0.020
Beta	PF/AC	5.986	0.019	6.330	0.016	6.888	0.012	2.819	0.101	7.799	0.008	5.893	0.020
	LPF	6.330	0.016	8.475	0.006	10.591	0.002	4.541	0.040	9.056	0.005	5.511	0.024
	C	6.888	0.012	10.591	0.002	12.725	0.001	6.198	0.017	14.358	0.001	8.992	0.005
	T	2.819	0.101	4.541	0.040	6.198	0.017	2.827	0.101	7.292	0.010	4.872	0.033
	P/IP	7.799	0.008	9.056	0.005	14.358	0.001	7.292	0.010	11.195	0.002	9.070	0.005
	O	5.893	0.020	5.511	0.024	8.992	0.005	4.872	0.033	9.070	0.005	0.911	0.346
Low gamma	PF/AC	0.461	0.501	0.389	0.537	4.542	0.040	0.142	0.708	4.426	0.042	6.512	0.015
	LPF	0.389	0.537	0.937	0.339	7.775	0.008	0.240	0.627	6.025	0.019	6.697	0.014
	C	4.542	0.040	7.775	0.008	1.083	0.305	0.023	0.882	4.983	0.032	7.624	0.009
	T	0.142	0.708	0.240	0.627	0.023	0.882	-0.676	0.416	0.598	0.444	1.346	0.253
	P/IP	4.426	0.042	6.025	0.019	4.983	0.032	0.598	0.444	0.536	0.469	3.338	0.076
	O	6.512	0.015	6.697	0.014	7.624	0.009	1.346	0.253	3.338	0.076	0.582	0.450
High	PF/AC	0.000	0.982	0.086	0.770	0.058	0.811	-0.047	0.830	0.004	0.947	0.003	0.955
	LPF	0.086	0.770	-0.268	0.607	-0.002	0.965	-0.325	0.572	-0.168	0.684	-0.029	0.867

C	0.058	0.811	-0.002	0.965	-0.002	0.969	-1.024	0.318	0.000	0.997	0.000	0.988
T	-0.047	0.830	-0.325	0.572	-1.024	0.318	-0.638	0.430	-0.915	0.345	-0.330	0.569
P/IP	0.004	0.947	-0.168	0.684	0.000	0.997	-0.915	0.345	-0.184	0.670	-0.061	0.806
O	0.003	0.955	-0.029	0.867	0.000	0.988	-0.330	0.569	-0.061	0.806	0.000	0.999

Supplementary Table S8. Group means, signed F-values and probabilities of region-specific NREM sleep EEG theta WPLI differences of Williams syndrome and typically developing subjects in all the possible electrode pairings. Color codes: Red = WS > TD (B-H corrected), Yellow = WS > TD (uncorrected), White = WS ≈ typically developing, Light blue = WS < TD (uncorrected), Blue = WS < TD (B-H corrected).

Theta	Mean WS	Mean TD	Signed F	p
Fp2-Fp2				
Fp2-F8	0.2533	0.2483	-0.0162	0.8993
Fp2-T4	0.1225	0.1438	1.0227	0.3183
Fp2-T6	0.1024	0.1072	0.0873	0.7693
Fp2-O2	0.0956	0.0898	-0.1629	0.6888
Fp2-Fp1	0.0613	0.0505	-0.8276	0.3687
Fp2-F7	0.1331	0.0996	-1.9661	0.1690
Fp2-T3	0.0828	0.0929	0.3840	0.5392
Fp2-T5	0.0893	0.0720	-1.1182	0.2970
Fp2-O1	0.0963	0.0762	-1.4022	0.2437
Fp2-F4	0.4671	0.3815	-3.1696	0.0830
Fp2-C4	0.2218	0.1994	-0.4120	0.5248
Fp2-P4	0.1146	0.0900	-2.0384	0.1615
Fp2-F3	0.2909	0.2398	-2.1078	0.1548
Fp2-C3	0.2052	0.1720	-1.2317	0.2741
Fp2-P3	0.1032	0.0768	-3.1321	0.0848
Fp2-Fpz1	0.2573	0.1939	-2.9100	0.0962
Fp2-Fz	0.3635	0.2779	-4.7638	0.0353
Fp2-Cz	0.2632	0.2130	-2.2388	0.1428
Fp2-Pz	0.1150	0.0772	-5.2966	0.0269
Fp2-Oz	0.1056	0.0900	-0.7839	0.3815
F8-Fp2	0.2533	0.2483	-0.0162	0.8993
F8-F8				
F8-T4	0.1908	0.0931	-11.2993	0.0018
F8-T6	0.1383	0.0977	-5.0175	0.0310
F8-O2	0.0918	0.0893	-0.0356	0.8514
F8-Fp1	0.1447	0.1530	0.0851	0.7722
F8-F7	0.0465	0.0665	2.6909	0.1092
F8-T3	0.0731	0.0745	0.0109	0.9173
F8-T5	0.0817	0.0775	-0.0645	0.8009
F8-O1	0.0875	0.0747	-0.7295	0.3984
F8-F4	0.2085	0.1491	-3.7620	0.0599
F8-C4	0.1328	0.1123	-0.7789	0.3830
F8-P4	0.1245	0.0755	-8.7854	0.0052
F8-F3	0.0933	0.0833	-0.3189	0.5756
F8-C3	0.1035	0.0783	-2.3498	0.1336
F8-P3	0.0925	0.0730	-1.8683	0.1797
F8-Fpz1	0.2612	0.2459	-0.1415	0.7088
F8-Fz	0.1464	0.0856	-5.0532	0.0305
F8-Cz	0.1551	0.1187	-2.1974	0.1465
F8-Pz	0.1127	0.0705	-7.1610	0.0109
F8-Oz	0.0979	0.0879	-0.2984	0.5881
T4-Fp2	0.1225	0.1438	1.0227	0.3183
T4-F8	0.1908	0.0931	-11.2993	0.0018
T4-T4				
T4-T6	0.0904	0.1011	0.2940	0.5908
T4-O2	0.0896	0.0916	0.0222	0.8823
T4-Fp1	0.1013	0.1298	2.5307	0.1199
T4-F7	0.0951	0.0831	-0.4778	0.4936
T4-T3	0.0517	0.0697	2.1501	0.1508

Theta	Mean WS	Mean TD	Signed F	p
T4-T5	0.0489	0.0579	0.9311	0.3407
T4-O1	0.0769	0.0590	-2.6281	0.1133
T4-F4	0.2802	0.1047	-16.2293	0.0003
T4-C4	0.2953	0.1429	-14.0345	0.0006
T4-P4	0.1155	0.0736	-5.1959	0.0283
T4-F3	0.1565	0.0876	-6.7502	0.0133
T4-C3	0.1735	0.0836	-10.9073	0.0021
T4-P3	0.0883	0.0493	-6.9813	0.0119
T4-Fpz1	0.1236	0.1610	2.7561	0.1051
T4-Fz	0.2064	0.0932	-10.3507	0.0026
T4-Cz	0.2284	0.1175	-9.6039	0.0036
T4-Pz	0.1023	0.0643	-5.3028	0.0269
T4-Oz	0.0849	0.0858	0.0039	0.9503
T6-Fp2	0.1024	0.1072	0.0873	0.7693
T6-F8	0.1383	0.0977	-5.0175	0.0310
T6-T4	0.0904	0.1011	0.2940	0.5908
T6-T6				
T6-O2	0.1136	0.1447	2.0940	0.1561
T6-Fp1	0.1018	0.1079	0.1151	0.7363
T6-F7	0.0980	0.0818	-1.0712	0.3072
T6-T3	0.0725	0.0725	0.0000	0.9998
T6-T5	0.0582	0.0491	-0.4565	0.5033
T6-O1	0.0963	0.0576	-5.2613	0.0274
T6-F4	0.2152	0.1009	-10.1247	0.0029
T6-C4	0.2917	0.1429	-12.1091	0.0013
T6-P4	0.2236	0.0983	-12.7477	0.0010
T6-F3	0.1405	0.0807	-6.1051	0.0181
T6-C3	0.1847	0.0984	-6.9693	0.0120
T6-P3	0.1415	0.0542	-12.1894	0.0012
T6-Fpz1	0.1055	0.1237	0.8291	0.3683
T6-Fz	0.1673	0.0870	-6.7488	0.0133
T6-Cz	0.2219	0.1245	-5.5860	0.0233
T6-Pz	0.1582	0.0976	-5.1442	0.0291
T6-Oz	0.1312	0.1135	-0.5232	0.4739
O2-Fp2	0.0956	0.0898	-0.1629	0.6888
O2-F8	0.0918	0.0893	-0.0356	0.8514
O2-T4	0.0896	0.0916	0.0222	0.8823
O2-T6	0.1136	0.1447	2.0940	0.1561
O2-O2				
O2-Fp1	0.1039	0.0887	-0.8673	0.3576
O2-F7	0.0886	0.0796	-0.4704	0.4970
O2-T3	0.0785	0.0765	-0.0352	0.8522
O2-T5	0.0837	0.0605	-2.6378	0.1126
O2-O1	0.1037	0.0556	-6.7175	0.0135
O2-F4	0.1197	0.0866	-1.6825	0.2024
O2-C4	0.1737	0.1038	-4.2761	0.0455
O2-P4	0.1608	0.0823	-6.4161	0.0156
O2-F3	0.1085	0.0798	-1.5055	0.2274
O2-C3	0.1482	0.1053	-1.8533	0.1814
O2-P3	0.1453	0.0874	-4.6483	0.0375
O2-Fpz1	0.1057	0.0975	-0.2389	0.6278
O2-Fz	0.1180	0.0827	-1.7919	0.1887
O2-Cz	0.1668	0.1137	-1.9734	0.1682
O2-Pz	0.1713	0.1220	-2.2389	0.1428
O2-Oz	0.2156	0.1944	-0.2677	0.6079
Fp1-Fp2	0.0613	0.0505	-0.8276	0.3687

Theta	Mean WS	Mean TD	Signed F	p
Fp1-F8	0.1447	0.1530	0.0851	0.7722
Fp1-T4	0.1013	0.1298	2.5307	0.1199
Fp1-T6	0.1018	0.1079	0.1151	0.7363
Fp1-O2	0.1039	0.0887	-0.8673	0.3576
Fp1-Fp1				
Fp1-F7	0.2503	0.2094	-1.0007	0.3235
Fp1-T3	0.1099	0.1275	0.7425	0.3943
Fp1-T5	0.1054	0.0890	-0.8216	0.3704
Fp1-O1	0.0828	0.0754	-0.2158	0.6449
Fp1-F4	0.3104	0.2625	-1.4049	0.2433
Fp1-C4	0.1836	0.1723	-0.1296	0.7209
Fp1-P4	0.1106	0.0882	-1.5184	0.2254
Fp1-F3	0.4497	0.3697	-2.9889	0.0920
Fp1-C3	0.2320	0.2072	-0.4575	0.5029
Fp1-P3	0.1049	0.0849	-1.4711	0.2327
Fp1-Fpz1	0.2373	0.1703	-3.5122	0.0686
Fp1-Fz	0.3711	0.2920	-3.6138	0.0649
Fp1-Cz	0.2580	0.2144	-1.5089	0.2269
Fp1-Pz	0.1121	0.0790	-3.6603	0.0633
Fp1-Oz	0.1022	0.0886	-0.5394	0.4672
F7-Fp2	0.1331	0.0996	-1.9661	0.1690
F7-F8	0.0465	0.0665	2.6909	0.1092
F7-T4	0.0951	0.0831	-0.4778	0.4936
F7-T6	0.0980	0.0818	-1.0712	0.3072
F7-O2	0.0886	0.0796	-0.4704	0.4970
F7-Fp1	0.2503	0.2094	-1.0007	0.3235
F7-F7				
F7-T3	0.1570	0.1021	-4.3987	0.0427
F7-T5	0.1393	0.0952	-2.6429	0.1123
F7-O1	0.0730	0.0743	0.0069	0.9342
F7-F4	0.1218	0.0950	-1.2612	0.2685
F7-C4	0.0977	0.0944	-0.0317	0.8597
F7-P4	0.0940	0.0662	-3.8577	0.0569
F7-F3	0.2113	0.1657	-2.2463	0.1422
F7-C3	0.1342	0.1359	0.0041	0.9494
F7-P3	0.1133	0.0725	-4.5538	0.0394
F7-Fpz1	0.2528	0.1989	-2.0214	0.1633
F7-Fz	0.1675	0.0901	-6.2653	0.0167
F7-Cz	0.1647	0.1300	-1.7677	0.1916
F7-Pz	0.0995	0.0689	-4.4746	0.0410
F7-Oz	0.0885	0.0832	-0.0865	0.7703
T3-Fp2	0.0828	0.0929	0.3840	0.5392
T3-F8	0.0731	0.0745	0.0109	0.9173
T3-T4	0.0517	0.0697	2.1501	0.1508
T3-T6	0.0725	0.0725	0.0000	0.9998
T3-O2	0.0785	0.0765	-0.0352	0.8522
T3-Fp1	0.1099	0.1275	0.7425	0.3943
T3-F7	0.1570	0.1021	-4.3987	0.0427
T3-T3				
T3-T5	0.0935	0.0849	-0.2568	0.6153
T3-O1	0.0647	0.0700	0.2617	0.6119
T3-F4	0.1493	0.0829	-6.1461	0.0177
T3-C4	0.1263	0.1043	-1.0261	0.3175
T3-P4	0.0588	0.0535	-0.2378	0.6286
T3-F3	0.2730	0.1345	-14.2128	0.0006
T3-C3	0.2887	0.1803	-9.5979	0.0037

Theta	Mean WS	Mean TD	Signed F	p
T3-P3	0.0732	0.0699	-0.0452	0.8327
T3-Fpz1	0.1204	0.1266	0.0718	0.7902
T3-Fz	0.2121	0.0868	-14.2368	0.0006
T3-Cz	0.2145	0.1402	-4.9091	0.0328
T3-Pz	0.0670	0.0612	-0.2122	0.6476
T3-Oz	0.0742	0.0761	0.0202	0.8878
T5-Fp2	0.0893	0.0720	-1.1182	0.2970
T5-F8	0.0817	0.0775	-0.0645	0.8009
T5-T4	0.0489	0.0579	0.9311	0.3407
T5-T6	0.0582	0.0491	-0.4565	0.5033
T5-O2	0.0837	0.0605	-2.6378	0.1126
T5-Fp1	0.1054	0.0890	-0.8216	0.3704
T5-F7	0.1393	0.0952	-2.6429	0.1123
T5-T3	0.0935	0.0849	-0.2568	0.6153
T5-T5				
T5-O1	0.1002	0.0841	-1.0694	0.3076
T5-F4	0.1424	0.1072	-1.5222	0.2249
T5-C4	0.1629	0.1447	-0.4605	0.5015
T5-P4	0.0962	0.0761	-1.8565	0.1811
T5-F3	0.2329	0.1508	-4.5780	0.0389
T5-C3	0.3186	0.2235	-5.2206	0.0280
T5-P3	0.2329	0.1607	-7.0509	0.0115
T5-Fpz1	0.0942	0.0842	-0.3236	0.5728
T5-Fz	0.1873	0.1077	-5.2713	0.0273
T5-Cz	0.2371	0.1839	-1.7987	0.1878
T5-Pz	0.1351	0.1298	-0.0522	0.8205
T5-Oz	0.1125	0.0825	-3.2098	0.0812
O1-Fp2	0.0963	0.0762	-1.4022	0.2437
O1-F8	0.0875	0.0747	-0.7295	0.3984
O1-T4	0.0769	0.0590	-2.6281	0.1133
O1-T6	0.0963	0.0576	-5.2613	0.0274
O1-O2	0.1037	0.0556	-6.7175	0.0135
O1-Fp1	0.0828	0.0754	-0.2158	0.6449
O1-F7	0.0730	0.0743	0.0069	0.9342
O1-T3	0.0647	0.0700	0.2617	0.6119
O1-T5	0.1002	0.0841	-1.0694	0.3076
O1-O1				
O1-F4	0.1072	0.0958	-0.2472	0.6219
O1-C4	0.1203	0.1137	-0.0562	0.8138
O1-P4	0.0926	0.0720	-1.1166	0.2973
O1-F3	0.1177	0.1011	-0.3503	0.5575
O1-C3	0.1799	0.1486	-0.7703	0.3856
O1-P3	0.1656	0.1213	-1.7964	0.1881
O1-Fpz1	0.0918	0.0806	-0.4478	0.5074
O1-Fz	0.1164	0.0910	-0.8667	0.3577
O1-Cz	0.1592	0.1415	-0.2234	0.6392
O1-Pz	0.1411	0.1396	-0.0027	0.9591
O1-Oz	0.1536	0.1397	-0.1969	0.6597
F4-Fp2	0.4671	0.3815	-3.1696	0.0830
F4-F8	0.2085	0.1491	-3.7620	0.0599
F4-T4	0.2802	0.1047	-16.2293	0.0003
F4-T6	0.2152	0.1009	-10.1247	0.0029
F4-O2	0.1197	0.0866	-1.6825	0.2024
F4-Fp1	0.3104	0.2625	-1.4049	0.2433
F4-F7	0.1218	0.0950	-1.2612	0.2685
F4-T3	0.1493	0.0829	-6.1461	0.0177

Theta	Mean WS	Mean TD	Signed F	p
F4-T5	0.1424	0.1072	-1.5222	0.2249
F4-O1	0.1072	0.0958	-0.2472	0.6219
F4-F4				
F4-C4	0.1460	0.1049	-2.7117	0.1079
F4-P4	0.2036	0.1396	-3.5220	0.0683
F4-F3	0.0720	0.0747	0.0228	0.8807
F4-C3	0.1055	0.0798	-3.7095	0.0616
F4-P3	0.1601	0.1174	-2.5209	0.1206
F4-Fpz1	0.4550	0.3881	-1.8309	0.1840
F4-Fz	0.1461	0.1415	-0.0200	0.8883
F4-Cz	0.1663	0.1470	-0.5223	0.4743
F4-Pz	0.1729	0.1167	-3.8016	0.0586
F4-Oz	0.1311	0.1077	-0.6950	0.4097
C4-Fp2	0.2218	0.1994	-0.4120	0.5248
C4-F8	0.1328	0.1123	-0.7789	0.3830
C4-T4	0.2953	0.1429	-14.0345	0.0006
C4-T6	0.2917	0.1429	-12.1091	0.0013
C4-O2	0.1737	0.1038	-4.2761	0.0455
C4-Fp1	0.1836	0.1723	-0.1296	0.7209
C4-F7	0.0977	0.0944	-0.0317	0.8597
C4-T3	0.1263	0.1043	-1.0261	0.3175
C4-T5	0.1629	0.1447	-0.4605	0.5015
C4-O1	0.1203	0.1137	-0.0562	0.8138
C4-F4	0.1460	0.1049	-2.7117	0.1079
C4-C4				
C4-P4	0.3176	0.2271	-4.8663	0.0335
C4-F3	0.0935	0.0837	-0.2654	0.6094
C4-C3	0.0549	0.0398	-1.8724	0.1792
C4-P3	0.1768	0.1539	-0.6498	0.4252
C4-Fpz1	0.2483	0.2208	-0.5627	0.4578
C4-Fz	0.1329	0.1441	0.1777	0.6757
C4-Cz	0.1452	0.0870	-3.3256	0.0761
C4-Pz	0.2434	0.1760	-4.2373	0.0464
C4-Oz	0.1600	0.1288	-0.8650	0.3582
P4-Fp2	0.1146	0.0900	-2.0384	0.1615
P4-F8	0.1245	0.0755	-8.7854	0.0052
P4-T4	0.1155	0.0736	-5.1959	0.0283
P4-T6	0.2236	0.0983	-12.7477	0.0010
P4-O2	0.1608	0.0823	-6.4161	0.0156
P4-Fp1	0.1106	0.0882	-1.5184	0.2254
P4-F7	0.0940	0.0662	-3.8577	0.0569
P4-T3	0.0588	0.0535	-0.2378	0.6286
P4-T5	0.0962	0.0761	-1.8565	0.1811
P4-O1	0.0926	0.0720	-1.1166	0.2973
P4-F4	0.2036	0.1396	-3.5220	0.0683
P4-C4	0.3176	0.2271	-4.8663	0.0335
P4-P4				
P4-F3	0.1527	0.1065	-2.8530	0.0994
P4-C3	0.2124	0.1580	-2.8634	0.0988
P4-P3	0.0686	0.0449	-2.2325	0.1434
P4-Fpz1	0.1326	0.1049	-1.6789	0.2029
P4-Fz	0.1817	0.1018	-5.9368	0.0196
P4-Cz	0.2807	0.2061	-2.8870	0.0975
P4-Pz	0.0898	0.0800	-0.3098	0.5811
P4-Oz	0.1167	0.0853	-1.5639	0.2187
F3-Fp2	0.2909	0.2398	-2.1078	0.1548

Theta	Mean WS	Mean TD	Signed F	p
F3-F8	0.0933	0.0833	-0.3189	0.5756
F3-T4	0.1565	0.0876	-6.7502	0.0133
F3-T6	0.1405	0.0807	-6.1051	0.0181
F3-O2	0.1085	0.0798	-1.5055	0.2274
F3-Fp1	0.4497	0.3697	-2.9889	0.0920
F3-F7	0.2113	0.1657	-2.2463	0.1422
F3-T3	0.2730	0.1345	-14.2128	0.0006
F3-T5	0.2329	0.1508	-4.5780	0.0389
F3-O1	0.1177	0.1011	-0.3503	0.5575
F3-F4	0.0720	0.0747	0.0228	0.8807
F3-C4	0.0935	0.0837	-0.2654	0.6094
F3-P4	0.1527	0.1065	-2.8530	0.0994
F3-F3				
F3-C3	0.1349	0.1311	-0.0245	0.8764
F3-P3	0.2027	0.1375	-4.1297	0.0492
F3-Fpz1	0.4401	0.3723	-1.9923	0.1662
F3-Fz	0.1731	0.1497	-0.4789	0.4931
F3-Cz	0.1872	0.1546	-1.2227	0.2758
F3-Pz	0.1683	0.1096	-4.4153	0.0423
F3-Oz	0.1289	0.1030	-0.8211	0.3706
C3-Fp2	0.2052	0.1720	-1.2317	0.2741
C3-F8	0.1035	0.0783	-2.3498	0.1336
C3-T4	0.1735	0.0836	-10.9073	0.0021
C3-T6	0.1847	0.0984	-6.9693	0.0120
C3-O2	0.1482	0.1053	-1.8533	0.1814
C3-Fp1	0.2320	0.2072	-0.4575	0.5029
C3-F7	0.1342	0.1359	0.0041	0.9494
C3-T3	0.2887	0.1803	-9.5979	0.0037
C3-T5	0.3186	0.2235	-5.2206	0.0280
C3-O1	0.1799	0.1486	-0.7703	0.3856
C3-F4	0.1055	0.0798	-3.7095	0.0616
C3-C4	0.0549	0.0398	-1.8724	0.1792
C3-P4	0.2124	0.1580	-2.8634	0.0988
C3-F3	0.1349	0.1311	-0.0245	0.8764
C3-C3				
C3-P3	0.3297	0.2475	-4.3093	0.0447
C3-Fpz1	0.2575	0.2254	-0.6946	0.4098
C3-Fz	0.1537	0.1586	0.0362	0.8501
C3-Cz	0.1241	0.0760	-3.0227	0.0902
C3-Pz	0.2706	0.1948	-5.6998	0.0220
C3-Oz	0.1698	0.1410	-0.6289	0.4327
P3-Fp2	0.1032	0.0768	-3.1321	0.0848
P3-F8	0.0925	0.0730	-1.8683	0.1797
P3-T4	0.0883	0.0493	-6.9813	0.0119
P3-T6	0.1415	0.0542	-12.1894	0.0012
P3-O2	0.1453	0.0874	-4.6483	0.0375
P3-Fp1	0.1049	0.0849	-1.4711	0.2327
P3-F7	0.1133	0.0725	-4.5538	0.0394
P3-T3	0.0732	0.0699	-0.0452	0.8327
P3-T5	0.2329	0.1607	-7.0509	0.0115
P3-O1	0.1656	0.1213	-1.7964	0.1881
P3-F4	0.1601	0.1174	-2.5209	0.1206
P3-C4	0.1768	0.1539	-0.6498	0.4252
P3-P4	0.0686	0.0449	-2.2325	0.1434
P3-F3	0.2027	0.1375	-4.1297	0.0492
P3-C3	0.3297	0.2475	-4.3093	0.0447

Theta	Mean WS	Mean TD	Signed F	p
P3-P3				
P3-Fpz1	0.1233	0.0917	-2.7413	0.1060
P3-Fz	0.1846	0.1059	-6.2073	0.0172
P3-Cz	0.2730	0.2161	-1.7664	0.1918
P3-Pz	0.0868	0.0878	0.0029	0.9570
P3-Oz	0.1433	0.1083	-1.4475	0.2364
Fpz1-Fp2	0.2573	0.1939	-2.9100	0.0962
Fpz1-F8	0.2612	0.2459	-0.1415	0.7088
Fpz1-T4	0.1236	0.1610	2.7561	0.1051
Fpz1-T6	0.1055	0.1237	0.8291	0.3683
Fpz1-O2	0.1057	0.0975	-0.2389	0.6278
Fpz1-Fp1	0.2373	0.1703	-3.5122	0.0686
Fpz1-F7	0.2528	0.1989	-2.0214	0.1633
Fpz1-T3	0.1204	0.1266	0.0718	0.7902
Fpz1-T5	0.0942	0.0842	-0.3236	0.5728
Fpz1-O1	0.0918	0.0806	-0.4478	0.5074
Fpz1-F4	0.4550	0.3881	-1.8309	0.1840
Fpz1-C4	0.2483	0.2208	-0.5627	0.4578
Fpz1-P4	0.1326	0.1049	-1.6789	0.2029
Fpz1-F3	0.4401	0.3723	-1.9923	0.1662
Fpz1-C3	0.2575	0.2254	-0.6946	0.4098
Fpz1-P3	0.1233	0.0917	-2.7413	0.1060
Fpz1-Fpz1				
Fpz1-Fz	0.4866	0.4040	-3.1119	0.0858
Fpz1-Cz	0.2950	0.2479	-1.5464	0.2213
Fpz1-Pz	0.1300	0.0905	-3.8322	0.0577
Fpz1-Oz	0.1058	0.0929	-0.5175	0.4763
Fz-Fp2	0.3635	0.2779	-4.7638	0.0353
Fz-F8	0.1464	0.0856	-5.0532	0.0305
Fz-T4	0.2064	0.0932	-10.3507	0.0026
Fz-T6	0.1673	0.0870	-6.7488	0.0133
Fz-O2	0.1180	0.0827	-1.7919	0.1887
Fz-Fp1	0.3711	0.2920	-3.6138	0.0649
Fz-F7	0.1675	0.0901	-6.2653	0.0167
Fz-T3	0.2121	0.0868	-14.2368	0.0006
Fz-T5	0.1873	0.1077	-5.2713	0.0273
Fz-O1	0.1164	0.0910	-0.8667	0.3577
Fz-F4	0.1461	0.1415	-0.0200	0.8883
Fz-C4	0.1329	0.1441	0.1777	0.6757
Fz-P4	0.1817	0.1018	-5.9368	0.0196
Fz-F3	0.1731	0.1497	-0.4789	0.4931
Fz-C3	0.1537	0.1586	0.0362	0.8501
Fz-P3	0.1846	0.1059	-6.2073	0.0172
Fz-Fpz1	0.4866	0.4040	-3.1119	0.0858
Fz-Fz				
Fz-Cz	0.2238	0.2356	0.1089	0.7432
Fz-Pz	0.1781	0.0959	-6.8190	0.0128
Fz-Oz	0.1369	0.0985	-1.6068	0.2127
Cz-Fp2	0.2632	0.2130	-2.2388	0.1428
Cz-F8	0.1551	0.1187	-2.1974	0.1465
Cz-T4	0.2284	0.1175	-9.6039	0.0036
Cz-T6	0.2219	0.1245	-5.5860	0.0233
Cz-O2	0.1668	0.1137	-1.9734	0.1682
Cz-Fp1	0.2580	0.2144	-1.5089	0.2269
Cz-F7	0.1647	0.1300	-1.7677	0.1916
Cz-T3	0.2145	0.1402	-4.9091	0.0328

Theta	Mean WS	Mean TD	Signed F	p
Cz-T5	0.2371	0.1839	-1.7987	0.1878
Cz-O1	0.1592	0.1415	-0.2234	0.6392
Cz-F4	0.1663	0.1470	-0.5223	0.4743
Cz-C4	0.1452	0.0870	-3.3256	0.0761
Cz-P4	0.2807	0.2061	-2.8870	0.0975
Cz-F3	0.1872	0.1546	-1.2227	0.2758
Cz-C3	0.1241	0.0760	-3.0227	0.0902
Cz-P3	0.2730	0.2161	-1.7664	0.1918
Cz-Fpz1	0.2950	0.2479	-1.5464	0.2213
Cz-Fz	0.2238	0.2356	0.1089	0.7432
Cz-Cz				
Cz-Pz	0.3211	0.2391	-3.4144	0.0724
Cz-Oz	0.1856	0.1482	-0.8373	0.3659
Pz-Fp2	0.1150	0.0772	-5.2966	0.0269
Pz-F8	0.1127	0.0705	-7.1610	0.0109
Pz-T4	0.1023	0.0643	-5.3028	0.0269
Pz-T6	0.1582	0.0976	-5.1442	0.0291
Pz-O2	0.1713	0.1220	-2.2389	0.1428
Pz-Fp1	0.1121	0.0790	-3.6603	0.0633
Pz-F7	0.0995	0.0689	-4.4746	0.0410
Pz-T3	0.0670	0.0612	-0.2122	0.6476
Pz-T5	0.1351	0.1298	-0.0522	0.8205
Pz-O1	0.1411	0.1396	-0.0027	0.9591
Pz-F4	0.1729	0.1167	-3.8016	0.0586
Pz-C4	0.2434	0.1760	-4.2373	0.0464
Pz-P4	0.0898	0.0800	-0.3098	0.5811
Pz-F3	0.1683	0.1096	-4.4153	0.0423
Pz-C3	0.2706	0.1948	-5.6998	0.0220
Pz-P3	0.0868	0.0878	0.0029	0.9570
Pz-Fpz1	0.1300	0.0905	-3.8322	0.0577
Pz-Fz	0.1781	0.0959	-6.8190	0.0128
Pz-Cz	0.3211	0.2391	-3.4144	0.0724
Pz-Pz				
Pz-Oz	0.1592	0.1479	-0.1088	0.7433
Oz-Fp2	0.1056	0.0900	-0.7839	0.3815
Oz-F8	0.0979	0.0879	-0.2984	0.5881
Oz-T4	0.0849	0.0858	0.0039	0.9503
Oz-T6	0.1312	0.1135	-0.5232	0.4739
Oz-O2	0.2156	0.1944	-0.2677	0.6079
Oz-Fp1	0.1022	0.0886	-0.5394	0.4672
Oz-F7	0.0885	0.0832	-0.0865	0.7703
Oz-T3	0.0742	0.0761	0.0202	0.8878
Oz-T5	0.1125	0.0825	-3.2098	0.0812
Oz-O1	0.1536	0.1397	-0.1969	0.6597
Oz-F4	0.1311	0.1077	-0.6950	0.4097
Oz-C4	0.1600	0.1288	-0.8650	0.3582
Oz-P4	0.1167	0.0853	-1.5639	0.2187
Oz-F3	0.1289	0.1030	-0.8211	0.3706
Oz-C3	0.1698	0.1410	-0.6289	0.4327
Oz-P3	0.1433	0.1083	-1.4475	0.2364
Oz-Fpz1	0.1058	0.0929	-0.5175	0.4763
Oz-Fz	0.1369	0.0985	-1.6068	0.2127
Oz-Cz	0.1856	0.1482	-0.8373	0.3659
Oz-Pz	0.1592	0.1479	-0.1088	0.7433
Oz-Oz				

Supplementary Table S9. Group means, signed F-values and probabilities of region-specific NREM sleep EEG alpha WPLI differences of Williams syndrome and typically developing subjects in all the possible electrode pairings. Color codes: Red = WS > TD (B-H corrected), Yellow = WS > TD (uncorrected), White = WS ≈ typically developing, Light blue = WS < TD (uncorrected), Blue = WS < TD (B-H corrected).

Alpha	Mean WS	Mean TD	Signed F	p
Fp2-Fp2				
Fp2-F8	0.4899	0.4855	-0.0092	0.9241
Fp2-T4	0.1994	0.2499	2.0267	0.1627
Fp2-T6	0.1733	0.1921	0.3475	0.5590
Fp2-O2	0.1926	0.2074	0.2105	0.6490
Fp2-Fp1	0.0832	0.1076	1.2395	0.2726
Fp2-F7	0.1966	0.1872	-0.1081	0.7441
Fp2-T3	0.1953	0.2008	0.0321	0.8587
Fp2-T5	0.1968	0.1737	-0.7407	0.3948
Fp2-O1	0.1979	0.2095	0.1353	0.7151
Fp2-F4	0.6747	0.6781	0.0083	0.9280
Fp2-C4	0.4294	0.4476	0.1372	0.7132
Fp2-P4	0.2521	0.2439	-0.0381	0.8463
Fp2-F3	0.3451	0.3692	0.4181	0.5218
Fp2-C3	0.3096	0.3294	0.2381	0.6284
Fp2-P3	0.2232	0.2363	0.1135	0.7381
Fp2-Fpz1	0.4015	0.3822	-0.2257	0.6375
Fp2-Fz	0.4772	0.4909	0.1246	0.7261
Fp2-Cz	0.3947	0.4231	0.4404	0.5109
Fp2-Pz	0.2378	0.2684	0.5767	0.4523
Fp2-Oz	0.2083	0.2392	0.9195	0.3437
F8-Fp2	0.4899	0.4855	-0.0092	0.9241
F8-F8				
F8-T4	0.1821	0.1407	-2.7053	0.1083
F8-T6	0.1656	0.1566	-0.1820	0.6721
F8-O2	0.1426	0.1645	1.1755	0.2851
F8-Fp1	0.2557	0.2816	0.5581	0.4596
F8-F7	0.0702	0.1087	4.3829	0.0430
F8-T3	0.0868	0.1257	3.5819	0.0660
F8-T5	0.1271	0.1425	0.4809	0.4922
F8-O1	0.1463	0.1550	0.1384	0.7120
F8-F4	0.2357	0.3117	2.0751	0.1579
F8-C4	0.2074	0.2643	1.4732	0.2323
F8-P4	0.1600	0.1702	0.1561	0.6950
F8-F3	0.0869	0.1193	3.8213	0.0580
F8-C3	0.1115	0.1586	3.8499	0.0571
F8-P3	0.1285	0.1613	1.9817	0.1673
F8-Fpz1	0.4493	0.4633	0.1012	0.7521
F8-Fz	0.1428	0.1539	0.2111	0.6485
F8-Cz	0.1667	0.2197	1.9234	0.1736
F8-Pz	0.1408	0.1823	2.5788	0.1166
F8-Oz	0.1541	0.1744	0.7083	0.4053
T4-Fp2	0.1994	0.2499	2.0267	0.1627
T4-F8	0.1821	0.1407	-2.7053	0.1083
T4-T4				
T4-T6	0.1343	0.1473	0.2869	0.5953
T4-O2	0.1454	0.1699	0.7566	0.3899
T4-Fp1	0.2165	0.2423	0.5654	0.4567
T4-F7	0.1217	0.1373	0.3750	0.5440
T4-T3	0.0583	0.1040	5.8447	0.0205

Alpha	Mean WS	Mean TD	Signed F	p
T4-T5	0.0747	0.1130	5.4049	0.0255
T4-O1	0.1118	0.1012	-0.3074	0.5825
T4-F4	0.2825	0.1809	-6.5742	0.0144
T4-C4	0.3502	0.2666	-4.6038	0.0384
T4-P4	0.1863	0.1957	0.0702	0.7925
T4-F3	0.1123	0.1233	0.3732	0.5449
T4-C3	0.1083	0.1130	0.0765	0.7836
T4-P3	0.0828	0.1087	1.7464	0.1942
T4-Fpz1	0.2781	0.2949	0.1652	0.6867
T4-Fz	0.1441	0.1365	-0.1653	0.6866
T4-Cz	0.2123	0.2119	-0.0002	0.9895
T4-Pz	0.1346	0.1657	1.3036	0.2607
T4-Oz	0.1486	0.1502	0.0033	0.9544
T6-Fp2	0.1733	0.1921	0.3475	0.5590
T6-F8	0.1656	0.1566	-0.1820	0.6721
T6-T4	0.1343	0.1473	0.2869	0.5953
T6-T6				
T6-O2	0.1826	0.2436	2.8966	0.0969
T6-Fp1	0.2251	0.2365	0.1080	0.7442
T6-F7	0.1650	0.1751	0.1416	0.7088
T6-T3	0.1046	0.1214	1.0115	0.3209
T6-T5	0.0677	0.1050	8.4053	0.0062
T6-O1	0.0951	0.1069	0.3913	0.5354
T6-F4	0.2414	0.2101	-0.7788	0.3831
T6-C4	0.3165	0.2918	-0.4731	0.4958
T6-P4	0.2795	0.2686	-0.1037	0.7492
T6-F3	0.1669	0.1492	-0.3998	0.5310
T6-C3	0.1360	0.1586	1.0069	0.3220
T6-P3	0.1059	0.1055	-0.0006	0.9806
T6-Fpz1	0.2321	0.2462	0.1224	0.7284
T6-Fz	0.1789	0.1530	-1.1344	0.2936
T6-Cz	0.2167	0.2567	1.4669	0.2333
T6-Pz	0.1898	0.2160	0.7594	0.3890
T6-Oz	0.1751	0.2077	0.8383	0.3656
O2-Fp2	0.1926	0.2074	0.2105	0.6490
O2-F8	0.1426	0.1645	1.1755	0.2851
O2-T4	0.1454	0.1699	0.7566	0.3899
O2-T6	0.1826	0.2436	2.8966	0.0969
O2-O2				
O2-Fp1	0.2150	0.2480	0.9982	0.3241
O2-F7	0.1636	0.1967	1.8325	0.1838
O2-T3	0.1306	0.1588	2.4010	0.1295
O2-T5	0.1232	0.1691	4.2655	0.0458
O2-O1	0.0894	0.1248	2.9730	0.0928
O2-F4	0.1675	0.1678	0.0001	0.9935
O2-C4	0.1730	0.2027	0.6980	0.4087
O2-P4	0.1625	0.1682	0.0454	0.8324
O2-F3	0.1849	0.1798	-0.0292	0.8652
O2-C3	0.1409	0.1691	0.9019	0.3483
O2-P3	0.1126	0.1455	2.4711	0.1242
O2-Fpz1	0.2216	0.2718	1.9723	0.1683
O2-Fz	0.1892	0.1735	-0.3413	0.5625
O2-Cz	0.1813	0.2273	1.5586	0.2195
O2-Pz	0.1673	0.1802	0.1509	0.6999
O2-Oz	0.1494	0.1816	0.8551	0.3609
Fp1-Fp2	0.0832	0.1076	1.2395	0.2726

Alpha	Mean WS	Mean TD	Signed F	p
Fp1-F8	0.2557	0.2816	0.5581	0.4596
Fp1-T4	0.2165	0.2423	0.5654	0.4567
Fp1-T6	0.2251	0.2365	0.1080	0.7442
Fp1-O2	0.2150	0.2480	0.9982	0.3241
Fp1-Fp1				
Fp1-F7	0.4552	0.4540	-0.0006	0.9805
Fp1-T3	0.2206	0.2394	0.2435	0.6246
Fp1-T5	0.1907	0.1750	-0.3369	0.5650
Fp1-O1	0.1795	0.1812	0.0031	0.9556
Fp1-F4	0.4142	0.4366	0.3652	0.5492
Fp1-C4	0.3358	0.3604	0.3845	0.5389
Fp1-P4	0.2514	0.2648	0.1320	0.7184
Fp1-F3	0.6586	0.6438	-0.1617	0.6898
Fp1-C3	0.4339	0.4453	0.0599	0.8080
Fp1-P3	0.2578	0.2496	-0.0400	0.8426
Fp1-Fpz1	0.3346	0.2935	-1.0572	0.3104
Fp1-Fz	0.5120	0.5116	-0.0001	0.9909
Fp1-Cz	0.4101	0.4331	0.3181	0.5761
Fp1-Pz	0.2569	0.2767	0.2531	0.6178
Fp1-Oz	0.2057	0.2417	1.3070	0.2601
F7-Fp2	0.1966	0.1872	-0.1081	0.7441
F7-F8	0.0702	0.1087	4.3829	0.0430
F7-T4	0.1217	0.1373	0.3750	0.5440
F7-T6	0.1650	0.1751	0.1416	0.7088
F7-O2	0.1636	0.1967	1.8325	0.1838
F7-Fp1	0.4552	0.4540	-0.0006	0.9805
F7-F7				
F7-T3	0.1567	0.1684	0.1662	0.6858
F7-T5	0.1679	0.2129	1.4817	0.2310
F7-O1	0.1356	0.1824	2.5544	0.1183
F7-F4	0.0927	0.1749	16.3532	0.0002
F7-C4	0.1491	0.1961	2.9356	0.0948
F7-P4	0.1512	0.1824	1.4908	0.2296
F7-F3	0.2483	0.3052	1.2597	0.2687
F7-C3	0.2475	0.2810	0.5164	0.4768
F7-P3	0.1796	0.1874	0.0631	0.8031
F7-Fpz1	0.4057	0.3897	-0.1516	0.6992
F7-Fz	0.1233	0.1778	5.0659	0.0303
F7-Cz	0.2172	0.2411	0.4239	0.5189
F7-Pz	0.1610	0.2037	2.2057	0.1458
F7-Oz	0.1510	0.1959	2.9528	0.0939
T3-Fp2	0.1953	0.2008	0.0321	0.8587
T3-F8	0.0868	0.1257	3.5819	0.0660
T3-T4	0.0583	0.1040	5.8447	0.0205
T3-T6	0.1046	0.1214	1.0115	0.3209
T3-O2	0.1306	0.1588	2.4010	0.1295
T3-Fp1	0.2206	0.2394	0.2435	0.6246
T3-F7	0.1567	0.1684	0.1662	0.6858
T3-T3				
T3-T5	0.1372	0.1886	2.9818	0.0923
T3-O1	0.1276	0.1737	2.9777	0.0925
T3-F4	0.0873	0.1289	6.8132	0.0129
T3-C4	0.1283	0.1474	1.3151	0.2586
T3-P4	0.1167	0.1137	-0.0221	0.8827
T3-F3	0.2439	0.2042	-1.1760	0.2850
T3-C3	0.3836	0.3001	-6.1521	0.0177

Alpha	Mean WS	Mean TD	Signed F	p
T3-P3	0.2074	0.1910	-0.2153	0.6453
T3-Fpz1	0.2745	0.2647	-0.0584	0.8104
T3-Fz	0.1289	0.1447	0.7430	0.3941
T3-Cz	0.2441	0.2480	0.0193	0.8902
T3-Pz	0.1576	0.1612	0.0145	0.9048
T3-Oz	0.1419	0.1787	2.3037	0.1373
T5-Fp2	0.1968	0.1737	-0.7407	0.3948
T5-F8	0.1271	0.1425	0.4809	0.4922
T5-T4	0.0747	0.1130	5.4049	0.0255
T5-T6	0.0677	0.1050	8.4053	0.0062
T5-O2	0.1232	0.1691	4.2655	0.0458
T5-Fp1	0.1907	0.1750	-0.3369	0.5650
T5-F7	0.1679	0.2129	1.4817	0.2310
T5-T3	0.1372	0.1886	2.9818	0.0923
T5-T5				
T5-O1	0.1649	0.2265	3.2932	0.0775
T5-F4	0.1383	0.1665	1.1380	0.2928
T5-C4	0.1659	0.2254	6.0701	0.0184
T5-P4	0.1506	0.1499	-0.0011	0.9735
T5-F3	0.2364	0.2842	1.1922	0.2818
T5-C3	0.3795	0.4003	0.3518	0.5566
T5-P3	0.3532	0.3824	0.6751	0.4164
T5-Fpz1	0.2200	0.1825	-1.3912	0.2455
T5-Fz	0.1862	0.1931	0.0490	0.8260
T5-Cz	0.2706	0.3484	5.0496	0.0305
T5-Pz	0.2496	0.2949	1.9642	0.1692
T5-Oz	0.1889	0.2399	2.6557	0.1114
O1-Fp2	0.1979	0.2095	0.1353	0.7151
O1-F8	0.1463	0.1550	0.1384	0.7120
O1-T4	0.1118	0.1012	-0.3074	0.5825
O1-T6	0.0951	0.1069	0.3913	0.5354
O1-O2	0.0894	0.1248	2.9730	0.0928
O1-Fp1	0.1795	0.1812	0.0031	0.9556
O1-F7	0.1356	0.1824	2.5544	0.1183
O1-T3	0.1276	0.1737	2.9777	0.0925
O1-T5	0.1649	0.2265	3.2932	0.0775
O1-O1				
O1-F4	0.1607	0.1653	0.0260	0.8726
O1-C4	0.1424	0.1937	3.5361	0.0677
O1-P4	0.1229	0.1256	0.0230	0.8804
O1-F3	0.1662	0.2154	2.1476	0.1510
O1-C3	0.2119	0.2760	3.0719	0.0877
O1-P3	0.2091	0.2230	0.1331	0.7173
O1-Fpz1	0.2008	0.2262	0.6033	0.4421
O1-Fz	0.1759	0.1692	-0.0575	0.8118
O1-Cz	0.2027	0.2839	5.3144	0.0267
O1-Pz	0.1938	0.2436	2.1131	0.1543
O1-Oz	0.1524	0.2378	7.9360	0.0076
F4-Fp2	0.6747	0.6781	0.0083	0.9280
F4-F8	0.2357	0.3117	2.0751	0.1579
F4-T4	0.2825	0.1809	-6.5742	0.0144
F4-T6	0.2414	0.2101	-0.7788	0.3831
F4-O2	0.1675	0.1678	0.0001	0.9935
F4-Fp1	0.4142	0.4366	0.3652	0.5492
F4-F7	0.0927	0.1749	16.3532	0.0002
F4-T3	0.0873	0.1289	6.8132	0.0129

Alpha	Mean WS	Mean TD	Signed F	p
F4-T5	0.1383	0.1665	1.1380	0.2928
F4-O1	0.1607	0.1653	0.0260	0.8726
F4-F4				
F4-C4	0.2429	0.2495	0.0211	0.8853
F4-P4	0.2135	0.2346	0.2714	0.6054
F4-F3	0.0897	0.1350	3.6763	0.0627
F4-C3	0.1386	0.1586	0.5072	0.4807
F4-P3	0.1590	0.1787	0.4024	0.5297
F4-Fpz1	0.6322	0.6477	0.1769	0.6765
F4-Fz	0.2944	0.3284	0.4546	0.5042
F4-Cz	0.2203	0.2195	-0.0004	0.9847
F4-Pz	0.1843	0.2009	0.2405	0.6266
F4-Oz	0.1708	0.1767	0.0375	0.8474
C4-Fp2	0.4294	0.4476	0.1372	0.7132
C4-F8	0.2074	0.2643	1.4732	0.2323
C4-T4	0.3502	0.2666	-4.6038	0.0384
C4-T6	0.3165	0.2918	-0.4731	0.4958
C4-O2	0.1730	0.2027	0.6980	0.4087
C4-Fp1	0.3358	0.3604	0.3845	0.5389
C4-F7	0.1491	0.1961	2.9356	0.0948
C4-T3	0.1283	0.1474	1.3151	0.2586
C4-T5	0.1659	0.2254	6.0701	0.0184
C4-O1	0.1424	0.1937	3.5361	0.0677
C4-F4	0.2429	0.2495	0.0211	0.8853
C4-C4				
C4-P4	0.3009	0.3485	1.1075	0.2993
C4-F3	0.1936	0.1880	-0.0292	0.8653
C4-C3	0.0549	0.0730	2.7405	0.1061
C4-P3	0.1532	0.2084	3.1870	0.0822
C4-Fpz1	0.4443	0.4643	0.2051	0.6532
C4-Fz	0.3107	0.3174	0.0240	0.8777
C4-Cz	0.1543	0.1696	0.3541	0.5553
C4-Pz	0.2145	0.2263	0.0875	0.7690
C4-Oz	0.1559	0.1914	1.3986	0.2443
P4-Fp2	0.2521	0.2439	-0.0381	0.8463
P4-F8	0.1600	0.1702	0.1561	0.6950
P4-T4	0.1863	0.1957	0.0702	0.7925
P4-T6	0.2795	0.2686	-0.1037	0.7492
P4-O2	0.1625	0.1682	0.0454	0.8324
P4-Fp1	0.2514	0.2648	0.1320	0.7184
P4-F7	0.1512	0.1824	1.4908	0.2296
P4-T3	0.1167	0.1137	-0.0221	0.8827
P4-T5	0.1506	0.1499	-0.0011	0.9735
P4-O1	0.1229	0.1256	0.0230	0.8804
P4-F4	0.2135	0.2346	0.2714	0.6054
P4-C4	0.3009	0.3485	1.1075	0.2993
P4-P4				
P4-F3	0.1832	0.1812	-0.0050	0.9442
P4-C3	0.1523	0.1838	1.0368	0.3150
P4-P3	0.0625	0.0902	3.2175	0.0808
P4-Fpz1	0.3052	0.3220	0.1678	0.6844
P4-Fz	0.2204	0.1848	-1.3865	0.2463
P4-Cz	0.2658	0.3431	3.2671	0.0786
P4-Pz	0.1186	0.1975	7.8993	0.0078
P4-Oz	0.1261	0.1655	2.4386	0.1267
F3-Fp2	0.3451	0.3692	0.4181	0.5218

Alpha	Mean WS	Mean TD	Signed F	p
F3-F8	0.0869	0.1193	3.8213	0.0580
F3-T4	0.1123	0.1233	0.3732	0.5449
F3-T6	0.1669	0.1492	-0.3998	0.5310
F3-O2	0.1849	0.1798	-0.0292	0.8652
F3-Fp1	0.6586	0.6438	-0.1617	0.6898
F3-F7	0.2483	0.3052	1.2597	0.2687
F3-T3	0.2439	0.2042	-1.1760	0.2850
F3-T5	0.2364	0.2842	1.1922	0.2818
F3-O1	0.1662	0.2154	2.1476	0.1510
F3-F4	0.0897	0.1350	3.6763	0.0627
F3-C4	0.1936	0.1880	-0.0292	0.8653
F3-P4	0.1832	0.1812	-0.0050	0.9442
F3-F3				
F3-C3	0.2535	0.2918	0.5994	0.4436
F3-P3	0.2221	0.2408	0.2272	0.6363
F3-Fpz1	0.6017	0.5955	-0.0281	0.8678
F3-Fz	0.2111	0.2889	3.4626	0.0705
F3-Cz	0.2928	0.2801	-0.0991	0.7547
F3-Pz	0.2017	0.2230	0.3930	0.5345
F3-Oz	0.1782	0.1958	0.2788	0.6006
C3-Fp2	0.3096	0.3294	0.2381	0.6284
C3-F8	0.1115	0.1586	3.8499	0.0571
C3-T4	0.1083	0.1130	0.0765	0.7836
C3-T6	0.1360	0.1586	1.0069	0.3220
C3-O2	0.1409	0.1691	0.9019	0.3483
C3-Fp1	0.4339	0.4453	0.0599	0.8080
C3-F7	0.2475	0.2810	0.5164	0.4768
C3-T3	0.3836	0.3001	-6.1521	0.0177
C3-T5	0.3795	0.4003	0.3518	0.5566
C3-O1	0.2119	0.2760	3.0719	0.0877
C3-F4	0.1386	0.1586	0.5072	0.4807
C3-C4	0.0549	0.0730	2.7405	0.1061
C3-P4	0.1523	0.1838	1.0368	0.3150
C3-F3	0.2535	0.2918	0.5994	0.4436
C3-C3				
C3-P3	0.3000	0.3664	2.0447	0.1609
C3-Fpz1	0.4290	0.4439	0.1103	0.7416
C3-Fz	0.2891	0.3151	0.3505	0.5574
C3-Cz	0.1376	0.1899	2.8153	0.1016
C3-Pz	0.2233	0.2247	0.0013	0.9714
C3-Oz	0.1647	0.1979	0.9493	0.3361
P3-Fp2	0.2232	0.2363	0.1135	0.7381
P3-F8	0.1285	0.1613	1.9817	0.1673
P3-T4	0.0828	0.1087	1.7464	0.1942
P3-T6	0.1059	0.1055	-0.0006	0.9806
P3-O2	0.1126	0.1455	2.4711	0.1242
P3-Fp1	0.2578	0.2496	-0.0400	0.8426
P3-F7	0.1796	0.1874	0.0631	0.8031
P3-T3	0.2074	0.1910	-0.2153	0.6453
P3-T5	0.3532	0.3824	0.6751	0.4164
P3-O1	0.2091	0.2230	0.1331	0.7173
P3-F4	0.1590	0.1787	0.4024	0.5297
P3-C4	0.1532	0.2084	3.1870	0.0822
P3-P4	0.0625	0.0902	3.2175	0.0808
P3-F3	0.2221	0.2408	0.2272	0.6363
P3-C3	0.3000	0.3664	2.0447	0.1609

Alpha	Mean WS	Mean TD	Signed F	p
P3-P3				
P3-Fpz1	0.2895	0.2938	0.0099	0.9213
P3-Fz	0.2119	0.1876	-0.6719	0.4175
P3-Cz	0.2759	0.3647	4.7279	0.0360
P3-Pz	0.1227	0.2255	13.5447	0.0007
P3-Oz	0.1374	0.1922	5.0370	0.0307
Fpz1-Fp2	0.4015	0.3822	-0.2257	0.6375
Fpz1-F8	0.4493	0.4633	0.1012	0.7521
Fpz1-T4	0.2781	0.2949	0.1652	0.6867
Fpz1-T6	0.2321	0.2462	0.1224	0.7284
Fpz1-O2	0.2216	0.2718	1.9723	0.1683
Fpz1-Fp1	0.3346	0.2935	-1.0572	0.3104
Fpz1-F7	0.4057	0.3897	-0.1516	0.6992
Fpz1-T3	0.2745	0.2647	-0.0584	0.8104
Fpz1-T5	0.2200	0.1825	-1.3912	0.2455
Fpz1-O1	0.2008	0.2262	0.6033	0.4421
Fpz1-F4	0.6322	0.6477	0.1769	0.6765
Fpz1-C4	0.4443	0.4643	0.2051	0.6532
Fpz1-P4	0.3052	0.3220	0.1678	0.6844
Fpz1-F3	0.6017	0.5955	-0.0281	0.8678
Fpz1-C3	0.4290	0.4439	0.1103	0.7416
Fpz1-P3	0.2895	0.2938	0.0099	0.9213
Fpz1-Fpz1				
Fpz1-Fz	0.6473	0.6553	0.0516	0.8216
Fpz1-Cz	0.4534	0.4816	0.4400	0.5111
Fpz1-Pz	0.2915	0.3303	0.9037	0.3478
Fpz1-Oz	0.2215	0.2800	3.0081	0.0910
Fz-Fp2	0.4772	0.4909	0.1246	0.7261
Fz-F8	0.1428	0.1539	0.2111	0.6485
Fz-T4	0.1441	0.1365	-0.1653	0.6866
Fz-T6	0.1789	0.1530	-1.1344	0.2936
Fz-O2	0.1892	0.1735	-0.3413	0.5625
Fz-Fp1	0.5120	0.5116	-0.0001	0.9909
Fz-F7	0.1233	0.1778	5.0659	0.0303
Fz-T3	0.1289	0.1447	0.7430	0.3941
Fz-T5	0.1862	0.1931	0.0490	0.8260
Fz-O1	0.1759	0.1692	-0.0575	0.8118
Fz-F4	0.2944	0.3284	0.4546	0.5042
Fz-C4	0.3107	0.3174	0.0240	0.8777
Fz-P4	0.2204	0.1848	-1.3865	0.2463
Fz-F3	0.2111	0.2889	3.4626	0.0705
Fz-C3	0.2891	0.3151	0.3505	0.5574
Fz-P3	0.2119	0.1876	-0.6719	0.4175
Fz-Fpz1	0.6473	0.6553	0.0516	0.8216
Fz-Fz				
Fz-Cz	0.4324	0.4304	-0.0018	0.9664
Fz-Pz	0.2257	0.2115	-0.1969	0.6598
Fz-Oz	0.1981	0.1897	-0.0743	0.7867
Cz-Fp2	0.3947	0.4231	0.4404	0.5109
Cz-F8	0.1667	0.2197	1.9234	0.1736
Cz-T4	0.2123	0.2119	-0.0002	0.9895
Cz-T6	0.2167	0.2567	1.4669	0.2333
Cz-O2	0.1813	0.2273	1.5586	0.2195
Cz-Fp1	0.4101	0.4331	0.3181	0.5761
Cz-F7	0.2172	0.2411	0.4239	0.5189
Cz-T3	0.2441	0.2480	0.0193	0.8902

Alpha	Mean WS	Mean TD	Signed F	p
Cz-T5	0.2706	0.3484	5.0496	0.0305
Cz-O1	0.2027	0.2839	5.3144	0.0267
Cz-F4	0.2203	0.2195	-0.0004	0.9847
Cz-C4	0.1543	0.1696	0.3541	0.5553
Cz-P4	0.2658	0.3431	3.2671	0.0786
Cz-F3	0.2928	0.2801	-0.0991	0.7547
Cz-C3	0.1376	0.1899	2.8153	0.1016
Cz-P3	0.2759	0.3647	4.7279	0.0360
Cz-Fpz1	0.4534	0.4816	0.4400	0.5111
Cz-Fz	0.4324	0.4304	-0.0018	0.9664
Cz-Cz				
Cz-Pz	0.3065	0.3511	0.7770	0.3836
Cz-Oz	0.1962	0.2435	1.5849	0.2157
Pz-Fp2	0.2378	0.2684	0.5767	0.4523
Pz-F8	0.1408	0.1823	2.5788	0.1166
Pz-T4	0.1346	0.1657	1.3036	0.2607
Pz-T6	0.1898	0.2160	0.7594	0.3890
Pz-O2	0.1673	0.1802	0.1509	0.6999
Pz-Fp1	0.2569	0.2767	0.2531	0.6178
Pz-F7	0.1610	0.2037	2.2057	0.1458
Pz-T3	0.1576	0.1612	0.0145	0.9048
Pz-T5	0.2496	0.2949	1.9642	0.1692
Pz-O1	0.1938	0.2436	2.1131	0.1543
Pz-F4	0.1843	0.2009	0.2405	0.6266
Pz-C4	0.2145	0.2263	0.0875	0.7690
Pz-P4	0.1186	0.1975	7.8993	0.0078
Pz-F3	0.2017	0.2230	0.3930	0.5345
Pz-C3	0.2233	0.2247	0.0013	0.9714
Pz-P3	0.1227	0.2255	13.5447	0.0007
Pz-Fpz1	0.2915	0.3303	0.9037	0.3478
Pz-Fz	0.2257	0.2115	-0.1969	0.6598
Pz-Cz	0.3065	0.3511	0.7770	0.3836
Pz-Pz				
Pz-Oz	0.1810	0.1921	0.1299	0.7205
Oz-Fp2	0.2083	0.2392	0.9195	0.3437
Oz-F8	0.1541	0.1744	0.7083	0.4053
Oz-T4	0.1486	0.1502	0.0033	0.9544
Oz-T6	0.1751	0.2077	0.8383	0.3656
Oz-O2	0.1494	0.1816	0.8551	0.3609
Oz-Fp1	0.2057	0.2417	1.3070	0.2601
Oz-F7	0.1510	0.1959	2.9528	0.0939
Oz-T3	0.1419	0.1787	2.3037	0.1373
Oz-T5	0.1889	0.2399	2.6557	0.1114
Oz-O1	0.1524	0.2378	7.9360	0.0076
Oz-F4	0.1708	0.1767	0.0375	0.8474
Oz-C4	0.1559	0.1914	1.3986	0.2443
Oz-P4	0.1261	0.1655	2.4386	0.1267
Oz-F3	0.1782	0.1958	0.2788	0.6006
Oz-C3	0.1647	0.1979	0.9493	0.3361
Oz-P3	0.1374	0.1922	5.0370	0.0307
Oz-Fpz1	0.2215	0.2800	3.0081	0.0910
Oz-Fz	0.1981	0.1897	-0.0743	0.7867
Oz-Cz	0.1962	0.2435	1.5849	0.2157
Oz-Pz	0.1810	0.1921	0.1299	0.7205
Oz-Oz				

Supplementary Table S10. Group means, signed F-values and probabilities of region-specific NREM sleep EEG beta WPLI differences of Williams syndrome and typically developing subjects in all the possible electrode pairings. Color codes: Red = WS > TD (B-H corrected), Yellow = WS > TD (uncorrected), White = WS ≈ typically developing, Light blue = WS < TD (uncorrected), Blue = WS < TD (B-H corrected).

Beta	Mean WS	Mean TD	Signed F	p
Fp2-Fp2				
Fp2-F8	0.0657	0.0500	-0.9794	0.3286
Fp2-T4	0.0503	0.0435	-0.5257	0.4729
Fp2-T6	0.0506	0.0690	2.0524	0.1601
Fp2-O2	0.0375	0.0612	3.2332	0.0801
Fp2-Fp1	0.0325	0.0366	0.2129	0.6471
Fp2-F7	0.0439	0.0406	-0.1102	0.7418
Fp2-T3	0.0511	0.0626	0.7149	0.4031
Fp2-T5	0.0387	0.0536	1.6727	0.2037
Fp2-O1	0.0320	0.0616	6.0058	0.0190
Fp2-F4	0.0623	0.0840	0.6817	0.4141
Fp2-C4	0.0448	0.0740	2.5000	0.1221
Fp2-P4	0.0428	0.0718	2.9890	0.0920
Fp2-F3	0.0469	0.0655	0.9345	0.3398
Fp2-C3	0.0430	0.0728	2.5606	0.1178
Fp2-P3	0.0343	0.0640	3.6490	0.0637
Fp2-Fpz1	0.0594	0.0449	-0.7132	0.4037
Fp2-Fz	0.0534	0.0679	0.4377	0.5122
Fp2-Cz	0.0453	0.0809	3.4106	0.0726
Fp2-Pz	0.0341	0.0725	5.5354	0.0239
Fp2-Oz	0.0306	0.0659	7.1720	0.0109
F8-Fp2	0.0657	0.0500	-0.9794	0.3286
F8-F8				
F8-T4	0.0550	0.0460	-0.8782	0.3546
F8-T6	0.0547	0.0665	0.7960	0.3779
F8-O2	0.0386	0.0581	2.2716	0.1400
F8-Fp1	0.0455	0.0495	0.1706	0.6819
F8-F7	0.0566	0.0435	-0.8848	0.3528
F8-T3	0.0506	0.0623	0.9271	0.3417
F8-T5	0.0414	0.0500	0.6741	0.4167
F8-O1	0.0325	0.0586	5.1707	0.0287
F8-F4	0.0345	0.0623	2.9709	0.0929
F8-C4	0.0441	0.0788	4.6877	0.0367
F8-P4	0.0464	0.0757	3.6424	0.0639
F8-F3	0.0345	0.0550	2.3934	0.1301
F8-C3	0.0368	0.0649	3.2546	0.0792
F8-P3	0.0330	0.0589	3.4927	0.0694
F8-Fpz1	0.0625	0.0537	-0.3822	0.5401
F8-Fz	0.0375	0.0550	1.4714	0.2326
F8-Cz	0.0334	0.0799	7.6656	0.0087
F8-Pz	0.0282	0.0685	7.3589	0.0100
F8-Oz	0.0371	0.0648	4.4487	0.0416
T4-Fp2	0.0503	0.0435	-0.5257	0.4729
T4-F8	0.0550	0.0460	-0.8782	0.3546
T4-T4				
T4-T6	0.0537	0.0490	-0.1351	0.7152
T4-O2	0.0415	0.0479	0.2769	0.6018
T4-Fp1	0.0524	0.0508	-0.0180	0.8940
T4-F7	0.0551	0.0478	-0.3581	0.5531
T4-T3	0.0566	0.0445	-0.6760	0.4161

Beta	Mean WS	Mean TD	Signed F	p
T4-T5	0.0380	0.0366	-0.0334	0.8560
T4-O1	0.0337	0.0440	1.1437	0.2916
T4-F4	0.0433	0.0437	0.0028	0.9583
T4-C4	0.0500	0.0436	-0.3146	0.5782
T4-P4	0.0484	0.0494	0.0038	0.9514
T4-F3	0.0460	0.0497	0.1913	0.6643
T4-C3	0.0402	0.0422	0.0397	0.8432
T4-P3	0.0309	0.0404	1.0902	0.3030
T4-Fpz1	0.0586	0.0535	-0.1957	0.6607
T4-Fz	0.0477	0.0491	0.0281	0.8678
T4-Cz	0.0349	0.0444	1.0665	0.3083
T4-Pz	0.0282	0.0495	3.7682	0.0597
T4-Oz	0.0385	0.0506	0.9640	0.3324
T6-Fp2	0.0506	0.0690	2.0524	0.1601
T6-F8	0.0547	0.0665	0.7960	0.3779
T6-T4	0.0537	0.0490	-0.1351	0.7152
T6-T6				
T6-O2	0.0547	0.0542	-0.0019	0.9653
T6-Fp1	0.0571	0.0705	0.8150	0.3723
T6-F7	0.0493	0.0718	3.7186	0.0613
T6-T3	0.0564	0.0480	-0.2307	0.6337
T6-T5	0.0408	0.0403	-0.0023	0.9619
T6-O1	0.0285	0.0394	1.6836	0.2023
T6-F4	0.0630	0.0796	2.0465	0.1607
T6-C4	0.0444	0.0513	1.0680	0.3079
T6-P4	0.0430	0.0371	-0.1692	0.6832
T6-F3	0.0638	0.0826	2.4182	0.1282
T6-C3	0.0513	0.0551	0.1271	0.7235
T6-P3	0.0374	0.0433	0.4593	0.5021
T6-Fpz1	0.0621	0.0755	0.8852	0.3527
T6-Fz	0.0706	0.0915	2.4871	0.1231
T6-Cz	0.0599	0.0722	1.5161	0.2258
T6-Pz	0.0376	0.0454	0.5725	0.4539
T6-Oz	0.0427	0.0480	0.2141	0.6462
O2-Fp2	0.0375	0.0612	3.2332	0.0801
O2-F8	0.0386	0.0581	2.2716	0.1400
O2-T4	0.0415	0.0479	0.2769	0.6018
O2-T6	0.0547	0.0542	-0.0019	0.9653
O2-O2				
O2-Fp1	0.0324	0.0623	5.2062	0.0282
O2-F7	0.0367	0.0563	2.3347	0.1348
O2-T3	0.0359	0.0492	1.9288	0.1730
O2-T5	0.0276	0.0324	1.0689	0.3077
O2-O1	0.0293	0.0318	0.1699	0.6825
O2-F4	0.0365	0.0693	5.3700	0.0260
O2-C4	0.0381	0.0581	2.3359	0.1347
O2-P4	0.0399	0.0570	3.4046	0.0728
O2-F3	0.0325	0.0726	8.7876	0.0052
O2-C3	0.0281	0.0570	8.6943	0.0054
O2-P3	0.0245	0.0406	7.2319	0.0106
O2-Fpz1	0.0356	0.0626	4.0705	0.0507
O2-Fz	0.0373	0.0742	6.3741	0.0159
O2-Cz	0.0364	0.0682	7.2061	0.0107
O2-Pz	0.0281	0.0440	4.7126	0.0363
O2-Oz	0.0295	0.0363	0.7318	0.3977
Fp1-Fp2	0.0325	0.0366	0.2129	0.6471

Beta	Mean WS	Mean TD	Signed F	p
Fp1-F8	0.0455	0.0495	0.1706	0.6819
Fp1-T4	0.0524	0.0508	-0.0180	0.8940
Fp1-T6	0.0571	0.0705	0.8150	0.3723
Fp1-O2	0.0324	0.0623	5.2062	0.0282
Fp1-Fp1				
Fp1-F7	0.0504	0.0609	0.4698	0.4972
Fp1-T3	0.0453	0.0565	1.0225	0.3183
Fp1-T5	0.0354	0.0551	2.4783	0.1237
Fp1-O1	0.0363	0.0635	3.6663	0.0631
Fp1-F4	0.0481	0.0731	2.0374	0.1616
Fp1-C4	0.0456	0.0753	3.0309	0.0898
Fp1-P4	0.0357	0.0743	6.0339	0.0187
Fp1-F3	0.0567	0.0915	2.0237	0.1630
Fp1-C3	0.0427	0.0811	3.6739	0.0628
Fp1-P3	0.0329	0.0685	4.5361	0.0397
Fp1-Fpz1	0.0482	0.0545	0.1232	0.7276
Fp1-Fz	0.0497	0.0751	1.5501	0.2207
Fp1-Cz	0.0498	0.0852	2.7454	0.1058
Fp1-Pz	0.0361	0.0766	5.3520	0.0262
Fp1-Oz	0.0307	0.0673	6.6775	0.0137
F7-Fp2	0.0439	0.0406	-0.1102	0.7418
F7-F8	0.0566	0.0435	-0.8848	0.3528
F7-T4	0.0551	0.0478	-0.3581	0.5531
F7-T6	0.0493	0.0718	3.7186	0.0613
F7-O2	0.0367	0.0563	2.3347	0.1348
F7-Fp1	0.0504	0.0609	0.4698	0.4972
F7-F7				
F7-T3	0.0485	0.0517	0.0642	0.8013
F7-T5	0.0420	0.0490	0.2843	0.5970
F7-O1	0.0418	0.0563	0.9463	0.3368
F7-F4	0.0491	0.0519	0.0354	0.8518
F7-C4	0.0438	0.0654	1.5093	0.2268
F7-P4	0.0446	0.0703	2.1687	0.1491
F7-F3	0.0496	0.0676	0.7228	0.4006
F7-C3	0.0449	0.0784	2.6324	0.1130
F7-P3	0.0387	0.0652	2.0586	0.1595
F7-Fpz1	0.0583	0.0521	-0.1240	0.7266
F7-Fz	0.0526	0.0487	-0.0534	0.8186
F7-Cz	0.0501	0.0695	0.8532	0.3615
F7-Pz	0.0401	0.0739	2.9235	0.0955
F7-Oz	0.0393	0.0627	2.1926	0.1469
T3-Fp2	0.0511	0.0626	0.7149	0.4031
T3-F8	0.0506	0.0623	0.9271	0.3417
T3-T4	0.0566	0.0445	-0.6760	0.4161
T3-T6	0.0564	0.0480	-0.2307	0.6337
T3-O2	0.0359	0.0492	1.9288	0.1730
T3-Fp1	0.0453	0.0565	1.0225	0.3183
T3-F7	0.0485	0.0517	0.0642	0.8013
T3-T3				
T3-T5	0.0439	0.0516	0.4221	0.5198
T3-O1	0.0462	0.0586	0.5985	0.4439
T3-F4	0.0502	0.0613	1.3186	0.2580
T3-C4	0.0517	0.0492	-0.0387	0.8450
T3-P4	0.0407	0.0494	0.5234	0.4738
T3-F3	0.0403	0.0573	4.2339	0.0465
T3-C3	0.0405	0.0553	1.9872	0.1668

Beta	Mean WS	Mean TD	Signed F	p
T3-P3	0.0400	0.0634	3.0912	0.0868
T3-Fpz1	0.0547	0.0667	0.6323	0.4314
T3-Fz	0.0579	0.0668	0.3379	0.5645
T3-Cz	0.0604	0.0517	-0.2891	0.5939
T3-Pz	0.0388	0.0628	2.7977	0.1026
T3-Oz	0.0363	0.0566	2.5226	0.1205
T5-Fp2	0.0387	0.0536	1.6727	0.2037
T5-F8	0.0414	0.0500	0.6741	0.4167
T5-T4	0.0380	0.0366	-0.0334	0.8560
T5-T6	0.0408	0.0403	-0.0023	0.9619
T5-O2	0.0276	0.0324	1.0689	0.3077
T5-Fp1	0.0354	0.0551	2.4783	0.1237
T5-F7	0.0420	0.0490	0.2843	0.5970
T5-T3	0.0439	0.0516	0.4221	0.5198
T5-T5				
T5-O1	0.0399	0.0451	0.4387	0.5117
T5-F4	0.0308	0.0484	3.5343	0.0678
T5-C4	0.0323	0.0404	1.1671	0.2868
T5-P4	0.0307	0.0343	0.2705	0.6060
T5-F3	0.0307	0.0563	5.9309	0.0197
T5-C3	0.0266	0.0453	4.4334	0.0419
T5-P3	0.0289	0.0357	0.8677	0.3575
T5-Fpz1	0.0392	0.0537	1.3862	0.2464
T5-Fz	0.0362	0.0507	1.5886	0.2152
T5-Cz	0.0343	0.0416	0.9384	0.3388
T5-Pz	0.0262	0.0365	2.6388	0.1125
T5-Oz	0.0326	0.0475	3.4907	0.0694
O1-Fp2	0.0320	0.0616	6.0058	0.0190
O1-F8	0.0325	0.0586	5.1707	0.0287
O1-T4	0.0337	0.0440	1.1437	0.2916
O1-T6	0.0285	0.0394	1.6836	0.2023
O1-O2	0.0293	0.0318	0.1699	0.6825
O1-Fp1	0.0363	0.0635	3.6663	0.0631
O1-F7	0.0418	0.0563	0.9463	0.3368
O1-T3	0.0462	0.0586	0.5985	0.4439
O1-T5	0.0399	0.0451	0.4387	0.5117
O1-O1				
O1-F4	0.0379	0.0791	8.8402	0.0051
O1-C4	0.0344	0.0616	5.4940	0.0244
O1-P4	0.0358	0.0449	0.7454	0.3934
O1-F3	0.0385	0.0792	8.1672	0.0069
O1-C3	0.0349	0.0627	4.8771	0.0333
O1-P3	0.0301	0.0414	1.8393	0.1830
O1-Fpz1	0.0359	0.0619	3.8175	0.0581
O1-Fz	0.0425	0.0853	7.7889	0.0082
O1-Cz	0.0391	0.0735	9.0399	0.0047
O1-Pz	0.0260	0.0410	2.9151	0.0959
O1-Oz	0.0353	0.0383	0.2383	0.6282
F4-Fp2	0.0623	0.0840	0.6817	0.4141
F4-F8	0.0345	0.0623	2.9709	0.0929
F4-T4	0.0433	0.0437	0.0028	0.9583
F4-T6	0.0630	0.0796	2.0465	0.1607
F4-O2	0.0365	0.0693	5.3700	0.0260
F4-Fp1	0.0481	0.0731	2.0374	0.1616
F4-F7	0.0491	0.0519	0.0354	0.8518
F4-T3	0.0502	0.0613	1.3186	0.2580

Beta	Mean WS	Mean TD	Signed F	p
F4-T5	0.0308	0.0484	3.5343	0.0678
F4-O1	0.0379	0.0791	8.8402	0.0051
F4-F4				
F4-C4	0.0428	0.0916	12.0411	0.0013
F4-P4	0.0526	0.0974	7.2082	0.0107
F4-F3	0.0305	0.0408	1.7803	0.1901
F4-C3	0.0339	0.0757	7.7250	0.0084
F4-P3	0.0310	0.0736	7.0513	0.0115
F4-Fpz1	0.0630	0.0890	0.8997	0.3488
F4-Fz	0.0496	0.0458	-0.0565	0.8134
F4-Cz	0.0316	0.0976	15.5609	0.0003
F4-Pz	0.0286	0.0898	11.8803	0.0014
F4-Oz	0.0391	0.0897	10.0115	0.0031
C4-Fp2	0.0448	0.0740	2.5000	0.1221
C4-F8	0.0441	0.0788	4.6877	0.0367
C4-T4	0.0500	0.0436	-0.3146	0.5782
C4-T6	0.0444	0.0513	1.0680	0.3079
C4-O2	0.0381	0.0581	2.3359	0.1347
C4-Fp1	0.0456	0.0753	3.0309	0.0898
C4-F7	0.0438	0.0654	1.5093	0.2268
C4-T3	0.0517	0.0492	-0.0387	0.8450
C4-T5	0.0323	0.0404	1.1671	0.2868
C4-O1	0.0344	0.0616	5.4940	0.0244
C4-F4	0.0428	0.0916	12.0411	0.0013
C4-C4				
C4-P4	0.0462	0.0586	0.6121	0.4389
C4-F3	0.0362	0.0776	11.1776	0.0019
C4-C3	0.0312	0.0391	0.8182	0.3714
C4-P3	0.0286	0.0460	2.3721	0.1318
C4-Fpz1	0.0487	0.0801	2.4183	0.1282
C4-Fz	0.0528	0.0996	6.6593	0.0139
C4-Cz	0.0358	0.0510	2.2105	0.1453
C4-Pz	0.0330	0.0667	4.3081	0.0447
C4-Oz	0.0376	0.0757	5.8123	0.0209
P4-Fp2	0.0428	0.0718	2.9890	0.0920
P4-F8	0.0464	0.0757	3.6424	0.0639
P4-T4	0.0484	0.0494	0.0038	0.9514
P4-T6	0.0430	0.0371	-0.1692	0.6832
P4-O2	0.0399	0.0570	3.4046	0.0728
P4-Fp1	0.0357	0.0743	6.0339	0.0187
P4-F7	0.0446	0.0703	2.1687	0.1491
P4-T3	0.0407	0.0494	0.5234	0.4738
P4-T5	0.0307	0.0343	0.2705	0.6060
P4-O1	0.0358	0.0449	0.7454	0.3934
P4-F4	0.0526	0.0974	7.2082	0.0107
P4-C4	0.0462	0.0586	0.6121	0.4389
P4-P4				
P4-F3	0.0453	0.0943	9.0344	0.0047
P4-C3	0.0339	0.0600	6.1069	0.0181
P4-P3	0.0260	0.0320	0.9830	0.3277
P4-Fpz1	0.0423	0.0755	3.5745	0.0663
P4-Fz	0.0609	0.1072	5.6512	0.0226
P4-Cz	0.0581	0.0801	1.6956	0.2007
P4-Pz	0.0338	0.0387	0.1482	0.7024
P4-Oz	0.0420	0.0701	3.6346	0.0642
F3-Fp2	0.0469	0.0655	0.9345	0.3398

Beta	Mean WS	Mean TD	Signed F	p
F3-F8	0.0345	0.0550	2.3934	0.1301
F3-T4	0.0460	0.0497	0.1913	0.6643
F3-T6	0.0638	0.0826	2.4182	0.1282
F3-O2	0.0325	0.0726	8.7876	0.0052
F3-Fp1	0.0567	0.0915	2.0237	0.1630
F3-F7	0.0496	0.0676	0.7228	0.4006
F3-T3	0.0403	0.0573	4.2339	0.0465
F3-T5	0.0307	0.0563	5.9309	0.0197
F3-O1	0.0385	0.0792	8.1672	0.0069
F3-F4	0.0305	0.0408	1.7803	0.1901
F3-C4	0.0362	0.0776	11.1776	0.0019
F3-P4	0.0453	0.0943	9.0344	0.0047
F3-F3				
F3-C3	0.0353	0.0936	9.9604	0.0031
F3-P3	0.0324	0.0840	8.2661	0.0066
F3-Fpz1	0.0600	0.0893	1.1238	0.2958
F3-Fz	0.0489	0.0541	0.0807	0.7779
F3-Cz	0.0382	0.0980	10.8429	0.0022
F3-Pz	0.0321	0.0990	12.5610	0.0011
F3-Oz	0.0358	0.0911	11.7671	0.0015
C3-Fp2	0.0430	0.0728	2.5606	0.1178
C3-F8	0.0368	0.0649	3.2546	0.0792
C3-T4	0.0402	0.0422	0.0397	0.8432
C3-T6	0.0513	0.0551	0.1271	0.7235
C3-O2	0.0281	0.0570	8.6943	0.0054
C3-Fp1	0.0427	0.0811	3.6739	0.0628
C3-F7	0.0449	0.0784	2.6324	0.1130
C3-T3	0.0405	0.0553	1.9872	0.1668
C3-T5	0.0266	0.0453	4.4334	0.0419
C3-O1	0.0349	0.0627	4.8771	0.0333
C3-F4	0.0339	0.0757	7.7250	0.0084
C3-C4	0.0312	0.0391	0.8182	0.3714
C3-P4	0.0339	0.0600	6.1069	0.0181
C3-F3	0.0353	0.0936	9.9604	0.0031
C3-C3				
C3-P3	0.0315	0.0659	6.3474	0.0161
C3-Fpz1	0.0475	0.0831	2.5683	0.1173
C3-Fz	0.0476	0.1034	6.2199	0.0171
C3-Cz	0.0405	0.0469	0.2312	0.6334
C3-Pz	0.0328	0.0778	7.5503	0.0091
C3-Oz	0.0346	0.0756	8.4137	0.0062
P3-Fp2	0.0343	0.0640	3.6490	0.0637
P3-F8	0.0330	0.0589	3.4927	0.0694
P3-T4	0.0309	0.0404	1.0902	0.3030
P3-T6	0.0374	0.0433	0.4593	0.5021
P3-O2	0.0245	0.0406	7.2319	0.0106
P3-Fp1	0.0329	0.0685	4.5361	0.0397
P3-F7	0.0387	0.0652	2.0586	0.1595
P3-T3	0.0400	0.0634	3.0912	0.0868
P3-T5	0.0289	0.0357	0.8677	0.3575
P3-O1	0.0301	0.0414	1.8393	0.1830
P3-F4	0.0310	0.0736	7.0513	0.0115
P3-C4	0.0286	0.0460	2.3721	0.1318
P3-P4	0.0260	0.0320	0.9830	0.3277
P3-F3	0.0324	0.0840	8.2661	0.0066
P3-C3	0.0315	0.0659	6.3474	0.0161

Beta	Mean WS	Mean TD	Signed F	p
P3-P3				
P3-Fpz1	0.0350	0.0669	3.4861	0.0696
P3-Fz	0.0386	0.0827	5.2324	0.0278
P3-Cz	0.0436	0.0667	3.2919	0.0775
P3-Pz	0.0252	0.0385	2.5823	0.1163
P3-Oz	0.0311	0.0631	7.0182	0.0117
Fpz1-Fp2	0.0594	0.0449	-0.7132	0.4037
Fpz1-F8	0.0625	0.0537	-0.3822	0.5401
Fpz1-T4	0.0586	0.0535	-0.1957	0.6607
Fpz1-T6	0.0621	0.0755	0.8852	0.3527
Fpz1-O2	0.0356	0.0626	4.0705	0.0507
Fpz1-Fp1	0.0482	0.0545	0.1232	0.7276
Fpz1-F7	0.0583	0.0521	-0.1240	0.7266
Fpz1-T3	0.0547	0.0667	0.6323	0.4314
Fpz1-T5	0.0392	0.0537	1.3862	0.2464
Fpz1-O1	0.0359	0.0619	3.8175	0.0581
Fpz1-F4	0.0630	0.0890	0.8997	0.3488
Fpz1-C4	0.0487	0.0801	2.4183	0.1282
Fpz1-P4	0.0423	0.0755	3.5745	0.0663
Fpz1-F3	0.0600	0.0893	1.1238	0.2958
Fpz1-C3	0.0475	0.0831	2.5683	0.1173
Fpz1-P3	0.0350	0.0669	3.4861	0.0696
Fpz1-Fpz1				
Fpz1-Fz	0.0624	0.0915	1.0300	0.3166
Fpz1-Cz	0.0529	0.0921	2.8827	0.0977
Fpz1-Pz	0.0381	0.0758	4.4031	0.0426
Fpz1-Oz	0.0303	0.0671	7.0983	0.0113
Fz-Fp2	0.0534	0.0679	0.4377	0.5122
Fz-F8	0.0375	0.0550	1.4714	0.2326
Fz-T4	0.0477	0.0491	0.0281	0.8678
Fz-T6	0.0706	0.0915	2.4871	0.1231
Fz-O2	0.0373	0.0742	6.3741	0.0159
Fz-Fp1	0.0497	0.0751	1.5501	0.2207
Fz-F7	0.0526	0.0487	-0.0534	0.8186
Fz-T3	0.0579	0.0668	0.3379	0.5645
Fz-T5	0.0362	0.0507	1.5886	0.2152
Fz-O1	0.0425	0.0853	7.7889	0.0082
Fz-F4	0.0496	0.0458	-0.0565	0.8134
Fz-C4	0.0528	0.0996	6.6593	0.0139
Fz-P4	0.0609	0.1072	5.6512	0.0226
Fz-F3	0.0489	0.0541	0.0807	0.7779
Fz-C3	0.0476	0.1034	6.2199	0.0171
Fz-P3	0.0386	0.0827	5.2324	0.0278
Fz-Fpz1	0.0624	0.0915	1.0300	0.3166
Fz-Fz				
Fz-Cz	0.0461	0.1279	13.5504	0.0007
Fz-Pz	0.0364	0.1019	10.5774	0.0024
Fz-Oz	0.0419	0.0964	9.8468	0.0033
Cz-Fp2	0.0453	0.0809	3.4106	0.0726
Cz-F8	0.0334	0.0799	7.6656	0.0087
Cz-T4	0.0349	0.0444	1.0665	0.3083
Cz-T6	0.0599	0.0722	1.5161	0.2258
Cz-O2	0.0364	0.0682	7.2061	0.0107
Cz-Fp1	0.0498	0.0852	2.7454	0.1058
Cz-F7	0.0501	0.0695	0.8532	0.3615
Cz-T3	0.0604	0.0517	-0.2891	0.5939

Beta	Mean WS	Mean TD	Signed F	p
Cz-T5	0.0343	0.0416	0.9384	0.3388
Cz-O1	0.0391	0.0735	9.0399	0.0047
Cz-F4	0.0316	0.0976	15.5609	0.0003
Cz-C4	0.0358	0.0510	2.2105	0.1453
Cz-P4	0.0581	0.0801	1.6956	0.2007
Cz-F3	0.0382	0.0980	10.8429	0.0022
Cz-C3	0.0405	0.0469	0.2312	0.6334
Cz-P3	0.0436	0.0667	3.2919	0.0775
Cz-Fpz1	0.0529	0.0921	2.8827	0.0977
Cz-Fz	0.0461	0.1279	13.5504	0.0007
Cz-Cz				
Cz-Pz	0.0448	0.0988	8.6714	0.0055
Cz-Oz	0.0448	0.0909	8.8266	0.0051
Pz-Fp2	0.0341	0.0725	5.5354	0.0239
Pz-F8	0.0282	0.0685	7.3589	0.0100
Pz-T4	0.0282	0.0495	3.7682	0.0597
Pz-T6	0.0376	0.0454	0.5725	0.4539
Pz-O2	0.0281	0.0440	4.7126	0.0363
Pz-Fp1	0.0361	0.0766	5.3520	0.0262
Pz-F7	0.0401	0.0739	2.9235	0.0955
Pz-T3	0.0388	0.0628	2.7977	0.1026
Pz-T5	0.0262	0.0365	2.6388	0.1125
Pz-O1	0.0260	0.0410	2.9151	0.0959
Pz-F4	0.0286	0.0898	11.8803	0.0014
Pz-C4	0.0330	0.0667	4.3081	0.0447
Pz-P4	0.0338	0.0387	0.1482	0.7024
Pz-F3	0.0321	0.0990	12.5610	0.0011
Pz-C3	0.0328	0.0778	7.5503	0.0091
Pz-P3	0.0252	0.0385	2.5823	0.1163
Pz-Fpz1	0.0381	0.0758	4.4031	0.0426
Pz-Fz	0.0364	0.1019	10.5774	0.0024
Pz-Cz	0.0448	0.0988	8.6714	0.0055
Pz-Pz				
Pz-Oz	0.0316	0.0667	5.5562	0.0237
Oz-Fp2	0.0306	0.0659	7.1720	0.0109
Oz-F8	0.0371	0.0648	4.4487	0.0416
Oz-T4	0.0385	0.0506	0.9640	0.3324
Oz-T6	0.0427	0.0480	0.2141	0.6462
Oz-O2	0.0295	0.0363	0.7318	0.3977
Oz-Fp1	0.0307	0.0673	6.6775	0.0137
Oz-F7	0.0393	0.0627	2.1926	0.1469
Oz-T3	0.0363	0.0566	2.5226	0.1205
Oz-T5	0.0326	0.0475	3.4907	0.0694
Oz-O1	0.0353	0.0383	0.2383	0.6282
Oz-F4	0.0391	0.0897	10.0115	0.0031
Oz-C4	0.0376	0.0757	5.8123	0.0209
Oz-P4	0.0420	0.0701	3.6346	0.0642
Oz-F3	0.0358	0.0911	11.7671	0.0015
Oz-C3	0.0346	0.0756	8.4137	0.0062
Oz-P3	0.0311	0.0631	7.0182	0.0117
Oz-Fpz1	0.0303	0.0671	7.0983	0.0113
Oz-Fz	0.0419	0.0964	9.8468	0.0033
Oz-Cz	0.0448	0.0909	8.8266	0.0051
Oz-Pz	0.0316	0.0667	5.5562	0.0237
Oz-Oz				

Supplementary Table S11. Probabilities for Fisher LSD Post Hoc Tests following a 3-way ANOVA with repeated measures (Group × Intrahemispheric/Interhemispheric × Band) conducted on intra- and interhemispheric REM sleep EEG broadband-1 and broadband-2 WPLI means of Williams syndrome and typically developing subjects. Significant differences are highlighted in red.

REM	Intra-left	Intra-right	Inter-HS
Broadband-1	0.000008	0.000013	0.000281
Broadband-2	0.057553	0.077433	0.095354

Supplementary Table S12. Probabilities for Fisher LSD Post Hoc Tests following a 2-way ANOVA with repeated measures (Group × Band) conducted on band-limited NREM sleep EEG global WPLI means of Williams syndrome and typically developing. Significant differences are highlighted in red.

REM	Fisher LSD p values
Slow oscillation	0.888960
Delta	0.803393
Theta	0.061174
Alpha	0.414559
Low sigma	0.006712
High sigma	0.002518
Beta	0.000005
Low gamma	0.092576
High gamma	0.889892

Supplementary Table S13. Probabilities for Fisher LSD Post Hoc Tests following a one-way ANOVA with repeated measures conducted on band-limited NREM sleep EEG intra- and interhemispheric WPLI means of Williams syndrome and typically developing subjects. Significant differences are highlighted in red.

REM	Intra-left	Intra-right	Inter-HS
Slow oscillation	0.818145	0.142291	0.709710
Delta	0.434450	0.723329	0.699920
Theta	0.000127	0.003799	0.028048
Alpha	0.054819	0.279671	0.051811
Low sigma	0.000001	0.000035	0.000017
High sigma	0.000001	0.000000	0.000132
Beta	0.000000	0.000000	0.000000
Low gamma	0.009566	0.061014	0.038574
High gamma	0.915925	0.902828	0.677172

Supplementary Table S14. Signed F-values and probabilities of region-specific REM sleep EEG Broadband-1 (0.5-30 Hz) and broadband-2 (0.5-100 Hz) WPLI differences between Williams syndrome and typically developing subjects (intra- and inter-regional pairings). Color codes: Red = WS > TD (B-H corrected), Yellow = WS > TD (uncorrected), White = WS ≈ typically developing, Light blue = WS < TD (uncorrected), Blue = WS < TD (B-H corrected).

**REM**		PF/AC		LPF		C		T		P/IP		O	
		Sign. F	p	Sign. F	p	Sign. F	p	Sign. F	p	Sign. F	p	Sign. F	p
**Broadband-1**	PF/AC	17.291	0.000	15.568	0.000	13.426	0.001	8.106	0.007	8.168	0.007	7.655	0.009
	LPF	15.568	0.000	19.550	0.000	17.017	0.000	7.730	0.008	11.552	0.002	7.691	0.009
	C	13.426	0.001	17.017	0.000	10.336	0.003	18.181	0.000	26.818	0.000	22.745	0.000
	T	8.106	0.007	7.730	0.008	18.181	0.000	7.360	0.010	15.016	0.000	7.185	0.011
	P/IP	8.168	0.007	11.552	0.002	26.818	0.000	15.016	0.000	29.698	0.000	18.055	0.000
	O	7.655	0.009	7.691	0.009	22.745	0.000	7.185	0.011	18.055	0.000	-0.011	0.917
**Broadband-2**	PF/AC	5.376	0.026	5.203	0.028	5.187	0.028	5.506	0.024	1.410	0.242	2.683	0.110
	LPF	5.203	0.028	11.945	0.001	14.836	0.000	4.244	0.046	10.536	0.002	12.044	0.001
	C	5.187	0.028	14.836	0.000	3.700	0.062	6.951	0.012	9.851	0.003	13.195	0.001
	T	5.506	0.024	4.244	0.046	6.951	0.012	1.609	0.212	7.094	0.011	6.414	0.016
	P/IP	1.410	0.242	10.536	0.002	9.851	0.003	7.094	0.011	6.913	0.012	12.381	0.001
	O	2.683	0.110	12.044	0.001	13.195	0.001	6.414	0.016	12.381	0.001	1.493	0.229

Supplementary Table S15. Group means, signed F-values and probabilities of region-specific REM sleep EEG Broadband-1 (0.5-30 Hz) WPLI differences of Williams syndrome and typically developing subjects in all the possible electrode pairings. Color codes: Red = WS > TD (B-H corrected), Yellow = WS > TD (uncorrected), White = WS ≈ typically developing, Light blue = WS < TD (uncorrected), Blue = WS < TD (B-H corrected).

Broadband-1	Mean WS	Mean TD	Signed F	p
Fp2-Fp2				
Fp2-F8	0.1483	0.1296	1.1126	0.2982
Fp2-T4	0.1305	0.1053	2.4828	0.1234
Fp2-T6	0.1404	0.0903	7.2592	0.0104
Fp2-O2	0.1227	0.0811	5.7663	0.0213
Fp2-Fp1	0.0753	0.0548	3.3150	0.0765
Fp2-F7	0.0971	0.0734	4.4446	0.0417
Fp2-T3	0.1113	0.0849	3.2362	0.0800
Fp2-T5	0.1066	0.0819	3.7105	0.0616
Fp2-O1	0.0942	0.0682	4.6819	0.0368
Fp2-F4	0.2441	0.1278	20.3430	0.0001
Fp2-C4	0.1771	0.1017	14.8980	0.0004
Fp2-P4	0.1173	0.0830	5.7156	0.0219
Fp2-F3	0.1442	0.0782	14.3237	0.0005
Fp2-C3	0.1381	0.1001	6.2264	0.0170
Fp2-P3	0.0985	0.0855	1.3124	0.2591
Fp2-Fpz1	0.1189	0.0957	1.8097	0.1865
Fp2-Fz	0.1756	0.0833	21.1802	0.0000
Fp2-Cz	0.1669	0.0963	13.5300	0.0007
Fp2-Pz	0.1159	0.0852	4.3615	0.0435
Fp2-Oz	0.1170	0.0794	5.5035	0.0243
F8-Fp2	0.1483	0.1296	1.1126	0.2982
F8-F8				
F8-T4	0.1376	0.1031	4.0904	0.0502
F8-T6	0.1542	0.1012	7.0254	0.0117
F8-O2	0.1326	0.0898	4.9844	0.0315
F8-Fp1	0.1150	0.0983	1.0029	0.3229
F8-F7	0.1067	0.0803	2.1852	0.1476
F8-T3	0.1108	0.0826	2.4339	0.1270
F8-T5	0.1116	0.0796	5.5296	0.0240
F8-O1	0.0941	0.0699	4.6112	0.0382
F8-F4	0.1648	0.0685	23.6542	0.0000
F8-C4	0.1583	0.0876	17.1124	0.0002
F8-P4	0.1166	0.0840	5.7886	0.0211
F8-F3	0.1004	0.0678	5.7107	0.0219
F8-C3	0.1038	0.0840	2.2660	0.1405
F8-P3	0.0932	0.0793	2.4844	0.1233
F8-Fpz1	0.1534	0.1330	1.0096	0.3213
F8-Fz	0.1109	0.0756	5.2547	0.0275
F8-Cz	0.1393	0.0795	16.2802	0.0003
F8-Pz	0.1104	0.0779	7.1174	0.0112
F8-Oz	0.1195	0.0855	4.3011	0.0449
T4-Fp2	0.1305	0.1053	2.4828	0.1234
T4-F8	0.1376	0.1031	4.0904	0.0502
T4-T4				
T4-T6	0.1581	0.1011	8.3737	0.0063
T4-O2	0.1436	0.0954	5.6451	0.0226
T4-Fp1	0.1271	0.0997	3.0737	0.0876
T4-F7	0.1351	0.0991	4.1628	0.0483
T4-T3	0.1200	0.0705	7.3601	0.0100

Broadband-1	Mean WS	Mean TD	Signed F	p
T4-T5	0.1079	0.0600	13.1207	0.0009
T4-O1	0.0957	0.0711	3.7873	0.0591
T4-F4	0.1105	0.0798	4.5477	0.0395
T4-C4	0.1288	0.0748	13.9124	0.0006
T4-P4	0.1198	0.0802	7.6398	0.0088
T4-F3	0.1052	0.0849	2.6121	0.1143
T4-C3	0.1001	0.0665	8.6998	0.0054
T4-P3	0.0930	0.0612	11.8130	0.0014
T4-Fpz1	0.1452	0.1105	3.9735	0.0534
T4-Fz	0.1104	0.0852	4.2538	0.0460
T4-Cz	0.1054	0.0670	7.3637	0.0099
T4-Pz	0.1050	0.0605	12.5110	0.0011
T4-Oz	0.1210	0.0855	3.9777	0.0533
T6-Fp2	0.1404	0.0903	7.2592	0.0104
T6-F8	0.1542	0.1012	7.0254	0.0117
T6-T4	0.1581	0.1011	8.3737	0.0063
T6-T6				
T6-O2	0.1587	0.1035	8.6776	0.0055
T6-Fp1	0.1414	0.0892	7.6790	0.0086
T6-F7	0.1481	0.0923	8.1720	0.0069
T6-T3	0.1373	0.0781	12.6833	0.0010
T6-T5	0.0867	0.0474	22.7481	0.0000
T6-O1	0.0909	0.0645	4.2057	0.0472
T6-F4	0.1166	0.0862	7.0805	0.0114
T6-C4	0.1193	0.0777	19.6669	0.0001
T6-P4	0.0961	0.0727	6.3412	0.0161
T6-F3	0.1183	0.0890	5.5040	0.0243
T6-C3	0.1084	0.0695	19.1637	0.0001
T6-P3	0.0823	0.0484	32.5579	0.0000
T6-Fpz1	0.1508	0.0932	8.0911	0.0071
T6-Fz	0.1172	0.0894	5.6187	0.0229
T6-Cz	0.1078	0.0727	17.7184	0.0002
T6-Pz	0.0971	0.0619	20.9541	0.0000
T6-Oz	0.1314	0.0940	4.2685	0.0457
O2-Fp2	0.1227	0.0811	5.7663	0.0213
O2-F8	0.1326	0.0898	4.9844	0.0315
O2-T4	0.1436	0.0954	5.6451	0.0226
O2-T6	0.1587	0.1035	8.6776	0.0055
O2-O2				
O2-Fp1	0.1183	0.0757	6.7088	0.0135
O2-F7	0.1182	0.0725	8.1485	0.0069
O2-T3	0.1126	0.0697	9.4450	0.0039
O2-T5	0.0915	0.0602	13.7340	0.0007
O2-O1	0.0700	0.0570	1.9441	0.1713
O2-F4	0.1125	0.0821	6.0214	0.0188
O2-C4	0.1102	0.0777	9.7721	0.0034
O2-P4	0.1163	0.0727	15.3946	0.0004
O2-F3	0.1126	0.0799	7.2118	0.0107
O2-C3	0.1042	0.0690	16.2720	0.0003
O2-P3	0.0957	0.0534	26.6154	0.0000
O2-Fpz1	0.1253	0.0809	6.3578	0.0160
O2-Fz	0.1145	0.0825	6.3047	0.0164
O2-Cz	0.1031	0.0712	17.4056	0.0002
O2-Pz	0.1000	0.0650	18.2234	0.0001
O2-Oz	0.0966	0.0955	0.0085	0.9271
Fp1-Fp2	0.0753	0.0548	3.3150	0.0765

Broadband-1	Mean WS	Mean TD	Signed F	p
Fp1-F8	0.1150	0.0983	1.0029	0.3229
Fp1-T4	0.1271	0.0997	3.0737	0.0876
Fp1-T6	0.1414	0.0892	7.6790	0.0086
Fp1-O2	0.1183	0.0757	6.7088	0.0135
Fp1-Fp1				
Fp1-F7	0.1202	0.0944	5.3409	0.0264
Fp1-T3	0.1195	0.0790	7.3611	0.0100
Fp1-T5	0.1119	0.0765	6.6316	0.0140
Fp1-O1	0.0976	0.0665	7.0919	0.0113
Fp1-F4	0.1805	0.0916	14.1844	0.0006
Fp1-C4	0.1601	0.0908	13.9365	0.0006
Fp1-P4	0.1193	0.0792	7.8128	0.0081
Fp1-F3	0.2299	0.1202	16.4378	0.0002
Fp1-C3	0.1707	0.1025	10.3274	0.0027
Fp1-P3	0.1063	0.0825	3.2209	0.0807
Fp1-Fpz1	0.0870	0.0668	4.1237	0.0493
Fp1-Fz	0.1955	0.0830	20.9884	0.0000
Fp1-Cz	0.1737	0.0916	15.0244	0.0004
Fp1-Pz	0.1229	0.0826	7.1875	0.0108
Fp1-Oz	0.1136	0.0729	7.1947	0.0108
F7-Fp2	0.0971	0.0734	4.4446	0.0417
F7-F8	0.1067	0.0803	2.1852	0.1476
F7-T4	0.1351	0.0991	4.1628	0.0483
F7-T6	0.1481	0.0923	8.1720	0.0069
F7-O2	0.1182	0.0725	8.1485	0.0069
F7-Fp1	0.1202	0.0944	5.3409	0.0264
F7-F7				
F7-T3	0.1097	0.0850	2.7453	0.1058
F7-T5	0.1156	0.0816	6.2727	0.0167
F7-O1	0.0954	0.0753	2.7635	0.1047
F7-F4	0.1406	0.0652	14.4923	0.0005
F7-C4	0.1474	0.0766	17.3980	0.0002
F7-P4	0.1201	0.0748	11.6156	0.0016
F7-F3	0.1910	0.0747	22.1109	0.0000
F7-C3	0.1722	0.0908	14.2691	0.0005
F7-P3	0.1083	0.0812	4.5448	0.0395
F7-Fpz1	0.1152	0.0923	3.4464	0.0712
F7-Fz	0.1433	0.0634	16.2992	0.0003
F7-Cz	0.1618	0.0747	18.8127	0.0001
F7-Pz	0.1243	0.0753	12.7667	0.0010
F7-Oz	0.1097	0.0851	1.7918	0.1887
T3-Fp2	0.1113	0.0849	3.2362	0.0800
T3-F8	0.1108	0.0826	2.4339	0.1270
T3-T4	0.1200	0.0705	7.3601	0.0100
T3-T6	0.1373	0.0781	12.6833	0.0010
T3-O2	0.1126	0.0697	9.4450	0.0039
T3-Fp1	0.1195	0.0790	7.3611	0.0100
T3-F7	0.1097	0.0850	2.7453	0.1058
T3-T3				
T3-T5	0.1179	0.0788	9.3744	0.0040
T3-O1	0.1064	0.0730	6.0504	0.0186
T3-F4	0.1141	0.0682	7.6484	0.0087
T3-C4	0.1256	0.0615	16.9763	0.0002
T3-P4	0.1138	0.0652	12.2428	0.0012
T3-F3	0.1365	0.0776	8.7473	0.0053
T3-C3	0.1744	0.0830	17.8444	0.0001

Broadband-1	Mean WS	Mean TD	Signed F	p
T3-P3	0.1356	0.0853	7.3713	0.0099
T3-Fpz1	0.1247	0.0888	4.9762	0.0317
T3-Fz	0.1179	0.0723	7.6743	0.0086
T3-Cz	0.1411	0.0635	19.6603	0.0001
T3-Pz	0.1280	0.0659	16.4926	0.0002
T3-Oz	0.1052	0.0681	7.4875	0.0094
T5-Fp2	0.1066	0.0819	3.7105	0.0616
T5-F8	0.1116	0.0796	5.5296	0.0240
T5-T4	0.1079	0.0600	13.1207	0.0009
T5-T6	0.0867	0.0474	22.7481	0.0000
T5-O2	0.0915	0.0602	13.7340	0.0007
T5-Fp1	0.1119	0.0765	6.6316	0.0140
T5-F7	0.1156	0.0816	6.2727	0.0167
T5-T3	0.1179	0.0788	9.3744	0.0040
T5-T5				
T5-O1	0.1172	0.0811	8.3265	0.0064
T5-F4	0.1078	0.0750	9.6768	0.0035
T5-C4	0.1122	0.0638	22.0673	0.0000
T5-P4	0.0887	0.0573	11.8499	0.0014
T5-F3	0.1212	0.0824	8.6907	0.0054
T5-C3	0.1502	0.0862	25.1311	0.0000
T5-P3	0.1421	0.0909	15.1140	0.0004
T5-Fpz1	0.1173	0.0846	4.9582	0.0320
T5-Fz	0.1088	0.0784	9.1881	0.0044
T5-Cz	0.1306	0.0752	26.0987	0.0000
T5-Pz	0.1274	0.0750	21.7672	0.0000
T5-Oz	0.1077	0.0775	7.9661	0.0075
O1-Fp2	0.0942	0.0682	4.6819	0.0368
O1-F8	0.0941	0.0699	4.6112	0.0382
O1-T4	0.0957	0.0711	3.7873	0.0591
O1-T6	0.0909	0.0645	4.2057	0.0472
O1-O2	0.0700	0.0570	1.9441	0.1713
O1-Fp1	0.0976	0.0665	7.0919	0.0113
O1-F7	0.0954	0.0753	2.7635	0.1047
O1-T3	0.1064	0.0730	6.0504	0.0186
O1-T5	0.1172	0.0811	8.3265	0.0064
O1-O1				
O1-F4	0.0994	0.0742	6.4606	0.0152
O1-C4	0.0955	0.0698	10.6914	0.0023
O1-P4	0.0855	0.0593	7.5624	0.0091
O1-F3	0.1017	0.0720	13.7478	0.0007
O1-C3	0.1082	0.0715	27.3584	0.0000
O1-P3	0.1050	0.0702	16.0044	0.0003
O1-Fpz1	0.1008	0.0701	5.7348	0.0217
O1-Fz	0.1004	0.0743	6.7324	0.0134
O1-Cz	0.1027	0.0699	25.1860	0.0000
O1-Pz	0.0966	0.0685	11.5053	0.0016
O1-Oz	0.0879	0.1046	-3.0886	0.0869
F4-Fp2	0.2441	0.1278	20.3430	0.0001
F4-F8	0.1648	0.0685	23.6542	0.0000
F4-T4	0.1105	0.0798	4.5477	0.0395
F4-T6	0.1166	0.0862	7.0805	0.0114
F4-O2	0.1125	0.0821	6.0214	0.0188
F4-Fp1	0.1805	0.0916	14.1844	0.0006
F4-F7	0.1406	0.0652	14.4923	0.0005
F4-T3	0.1141	0.0682	7.6484	0.0087

Broadband-1	Mean WS	Mean TD	Signed F	p
F4-T5	0.1078	0.0750	9.6768	0.0035
F4-O1	0.0994	0.0742	6.4606	0.0152
F4-F4				
F4-C4	0.1299	0.1106	3.0914	0.0868
F4-P4	0.1117	0.0936	3.6393	0.0640
F4-F3	0.0927	0.0529	8.2745	0.0066
F4-C3	0.1039	0.0953	0.9357	0.3395
F4-P3	0.1014	0.0879	1.8093	0.1866
F4-Fpz1	0.2384	0.1343	14.2948	0.0005
F4-Fz	0.1333	0.0951	3.6430	0.0639
F4-Cz	0.1415	0.0913	14.0924	0.0006
F4-Pz	0.1161	0.0943	4.4176	0.0423
F4-Oz	0.1230	0.0863	8.5615	0.0058
C4-Fp2	0.1771	0.1017	14.8980	0.0004
C4-F8	0.1583	0.0876	17.1124	0.0002
C4-T4	0.1288	0.0748	13.9124	0.0006
C4-T6	0.1193	0.0777	19.6669	0.0001
C4-O2	0.1102	0.0777	9.7721	0.0034
C4-Fp1	0.1601	0.0908	13.9365	0.0006
C4-F7	0.1474	0.0766	17.3980	0.0002
C4-T3	0.1256	0.0615	16.9763	0.0002
C4-T5	0.1122	0.0638	22.0673	0.0000
C4-O1	0.0955	0.0698	10.6914	0.0023
C4-F4	0.1299	0.1106	3.0914	0.0868
C4-C4				
C4-P4	0.1226	0.0880	9.2709	0.0042
C4-F3	0.1178	0.0882	7.4534	0.0095
C4-C3	0.0722	0.0442	17.3098	0.0002
C4-P3	0.0987	0.0673	9.0473	0.0046
C4-Fpz1	0.1815	0.1042	12.7414	0.0010
C4-Fz	0.1485	0.1168	5.1276	0.0293
C4-Cz	0.0954	0.0800	2.7964	0.1027
C4-Pz	0.1104	0.0752	10.1305	0.0029
C4-Oz	0.1166	0.0798	10.9792	0.0020
P4-Fp2	0.1173	0.0830	5.7156	0.0219
P4-F8	0.1166	0.0840	5.7886	0.0211
P4-T4	0.1198	0.0802	7.6398	0.0088
P4-T6	0.0961	0.0727	6.3412	0.0161
P4-O2	0.1163	0.0727	15.3946	0.0004
P4-Fp1	0.1193	0.0792	7.8128	0.0081
P4-F7	0.1201	0.0748	11.6156	0.0016
P4-T3	0.1138	0.0652	12.2428	0.0012
P4-T5	0.0887	0.0573	11.8499	0.0014
P4-O1	0.0855	0.0593	7.5624	0.0091
P4-F4	0.1117	0.0936	3.6393	0.0640
P4-C4	0.1226	0.0880	9.2709	0.0042
P4-P4				
P4-F3	0.1043	0.0873	3.1040	0.0861
P4-C3	0.1042	0.0641	28.5918	0.0000
P4-P3	0.0652	0.0392	35.9916	0.0000
P4-Fpz1	0.1282	0.0843	7.3574	0.0100
P4-Fz	0.1080	0.0989	0.8454	0.3637
P4-Cz	0.1239	0.0884	14.0911	0.0006
P4-Pz	0.0975	0.0555	22.6224	0.0000
P4-Oz	0.1221	0.0762	12.6148	0.0010
F3-Fp2	0.1442	0.0782	14.3237	0.0005

Broadband-1	Mean WS	Mean TD	Signed F	p
F3-F8	0.1004	0.0678	5.7107	0.0219
F3-T4	0.1052	0.0849	2.6121	0.1143
F3-T6	0.1183	0.0890	5.5040	0.0243
F3-O2	0.1126	0.0799	7.2118	0.0107
F3-Fp1	0.2299	0.1202	16.4378	0.0002
F3-F7	0.1910	0.0747	22.1109	0.0000
F3-T3	0.1365	0.0776	8.7473	0.0053
F3-T5	0.1212	0.0824	8.6907	0.0054
F3-O1	0.1017	0.0720	13.7478	0.0007
F3-F4	0.0927	0.0529	8.2745	0.0066
F3-C4	0.1178	0.0882	7.4534	0.0095
F3-P4	0.1043	0.0873	3.1040	0.0861
F3-F3				
F3-C3	0.1373	0.1118	3.7525	0.0602
F3-P3	0.1101	0.0938	4.7777	0.0351
F3-Fpz1	0.2085	0.1174	13.8551	0.0006
F3-Fz	0.1124	0.0644	18.7916	0.0001
F3-Cz	0.1553	0.0867	15.6121	0.0003
F3-Pz	0.1121	0.0941	3.4558	0.0708
F3-Oz	0.1173	0.0814	9.9181	0.0032
C3-Fp2	0.1381	0.1001	6.2264	0.0170
C3-F8	0.1038	0.0840	2.2660	0.1405
C3-T4	0.1001	0.0665	8.6998	0.0054
C3-T6	0.1084	0.0695	19.1637	0.0001
C3-O2	0.1042	0.0690	16.2720	0.0003
C3-Fp1	0.1707	0.1025	10.3274	0.0027
C3-F7	0.1722	0.0908	14.2691	0.0005
C3-T3	0.1744	0.0830	17.8444	0.0001
C3-T5	0.1502	0.0862	25.1311	0.0000
C3-O1	0.1082	0.0715	27.3584	0.0000
C3-F4	0.1039	0.0953	0.9357	0.3395
C3-C4	0.0722	0.0442	17.3098	0.0002
C3-P4	0.1042	0.0641	28.5918	0.0000
C3-F3	0.1373	0.1118	3.7525	0.0602
C3-C3				
C3-P3	0.1321	0.0900	12.9565	0.0009
C3-Fpz1	0.1645	0.1085	8.0324	0.0073
C3-Fz	0.1346	0.1154	2.8449	0.0999
C3-Cz	0.1055	0.0757	6.1939	0.0173
C3-Pz	0.1165	0.0755	21.9359	0.0000
C3-Oz	0.1142	0.0736	20.5158	0.0001
P3-Fp2	0.0985	0.0855	1.3124	0.2591
P3-F8	0.0932	0.0793	2.4844	0.1233
P3-T4	0.0930	0.0612	11.8130	0.0014
P3-T6	0.0823	0.0484	32.5579	0.0000
P3-O2	0.0957	0.0534	26.6154	0.0000
P3-Fp1	0.1063	0.0825	3.2209	0.0807
P3-F7	0.1083	0.0812	4.5448	0.0395
P3-T3	0.1356	0.0853	7.3713	0.0099
P3-T5	0.1421	0.0909	15.1140	0.0004
P3-O1	0.1050	0.0702	16.0044	0.0003
P3-F4	0.1014	0.0879	1.8093	0.1866
P3-C4	0.0987	0.0673	9.0473	0.0046
P3-P4	0.0652	0.0392	35.9916	0.0000
P3-F3	0.1101	0.0938	4.7777	0.0351
P3-C3	0.1321	0.0900	12.9565	0.0009

Broadband-1	Mean WS	Mean TD	Signed F	p
P3-P3				
P3-Fpz1	0.1098	0.0884	2.3597	0.1328
P3-Fz	0.1029	0.0961	0.6447	0.4270
P3-Cz	0.1338	0.0892	11.9412	0.0014
P3-Pz	0.1050	0.0604	14.1295	0.0006
P3-Oz	0.1151	0.0686	18.1036	0.0001
Fpz1-Fp2	0.1189	0.0957	1.8097	0.1865
Fpz1-F8	0.1534	0.1330	1.0096	0.3213
Fpz1-T4	0.1452	0.1105	3.9735	0.0534
Fpz1-T6	0.1508	0.0932	8.0911	0.0071
Fpz1-O2	0.1253	0.0809	6.3578	0.0160
Fpz1-Fp1	0.0870	0.0668	4.1237	0.0493
Fpz1-F7	0.1152	0.0923	3.4464	0.0712
Fpz1-T3	0.1247	0.0888	4.9762	0.0317
Fpz1-T5	0.1173	0.0846	4.9582	0.0320
Fpz1-O1	0.1008	0.0701	5.7348	0.0217
Fpz1-F4	0.2384	0.1343	14.2948	0.0005
Fpz1-C4	0.1815	0.1042	12.7414	0.0010
Fpz1-P4	0.1282	0.0843	7.3574	0.0100
Fpz1-F3	0.2085	0.1174	13.8551	0.0006
Fpz1-C3	0.1645	0.1085	8.0324	0.0073
Fpz1-P3	0.1098	0.0884	2.3597	0.1328
Fpz1-Fpz1				
Fpz1-Fz	0.2343	0.1124	20.6646	0.0001
Fpz1-Cz	0.1811	0.1017	12.8736	0.0009
Fpz1-Pz	0.1260	0.0885	5.3108	0.0268
Fpz1-Oz	0.1190	0.0779	6.6377	0.0140
Fz-Fp2	0.1756	0.0833	21.1802	0.0000
Fz-F8	0.1109	0.0756	5.2547	0.0275
Fz-T4	0.1104	0.0852	4.2538	0.0460
Fz-T6	0.1172	0.0894	5.6187	0.0229
Fz-O2	0.1145	0.0825	6.3047	0.0164
Fz-Fp1	0.1955	0.0830	20.9884	0.0000
Fz-F7	0.1433	0.0634	16.2992	0.0003
Fz-T3	0.1179	0.0723	7.6743	0.0086
Fz-T5	0.1088	0.0784	9.1881	0.0044
Fz-O1	0.1004	0.0743	6.7324	0.0134
Fz-F4	0.1333	0.0951	3.6430	0.0639
Fz-C4	0.1485	0.1168	5.1276	0.0293
Fz-P4	0.1080	0.0989	0.8454	0.3637
Fz-F3	0.1124	0.0644	18.7916	0.0001
Fz-C3	0.1346	0.1154	2.8449	0.0999
Fz-P3	0.1029	0.0961	0.6447	0.4270
Fz-Fpz1	0.2343	0.1124	20.6646	0.0001
Fz-Fz				
Fz-Cz	0.1997	0.1105	21.9801	0.0000
Fz-Pz	0.1158	0.1022	1.6573	0.2058
Fz-Oz	0.1213	0.0867	6.8689	0.0125
Cz-Fp2	0.1669	0.0963	13.5300	0.0007
Cz-F8	0.1393	0.0795	16.2802	0.0003
Cz-T4	0.1054	0.0670	7.3637	0.0099
Cz-T6	0.1078	0.0727	17.7184	0.0002
Cz-O2	0.1031	0.0712	17.4056	0.0002
Cz-Fp1	0.1737	0.0916	15.0244	0.0004
Cz-F7	0.1618	0.0747	18.8127	0.0001
Cz-T3	0.1411	0.0635	19.6603	0.0001

Broadband-1	Mean WS	Mean TD	Signed F	p
Cz-T5	0.1306	0.0752	26.0987	0.0000
Cz-O1	0.1027	0.0699	25.1860	0.0000
Cz-F4	0.1415	0.0913	14.0924	0.0006
Cz-C4	0.0954	0.0800	2.7964	0.1027
Cz-P4	0.1239	0.0884	14.0911	0.0006
Cz-F3	0.1553	0.0867	15.6121	0.0003
Cz-C3	0.1055	0.0757	6.1939	0.0173
Cz-P3	0.1338	0.0892	11.9412	0.0014
Cz-Fpz1	0.1811	0.1017	12.8736	0.0009
Cz-Fz	0.1997	0.1105	21.9801	0.0000
Cz-Cz				
Cz-Pz	0.1326	0.1006	6.5984	0.0143
Cz-Oz	0.1144	0.0793	16.9214	0.0002
Pz-Fp2	0.1159	0.0852	4.3615	0.0435
Pz-F8	0.1104	0.0779	7.1174	0.0112
Pz-T4	0.1050	0.0605	12.5110	0.0011
Pz-T6	0.0971	0.0619	20.9541	0.0000
Pz-O2	0.1000	0.0650	18.2234	0.0001
Pz-Fp1	0.1229	0.0826	7.1875	0.0108
Pz-F7	0.1243	0.0753	12.7667	0.0010
Pz-T3	0.1280	0.0659	16.4926	0.0002
Pz-T5	0.1274	0.0750	21.7672	0.0000
Pz-O1	0.0966	0.0685	11.5053	0.0016
Pz-F4	0.1161	0.0943	4.4176	0.0423
Pz-C4	0.1104	0.0752	10.1305	0.0029
Pz-P4	0.0975	0.0555	22.6224	0.0000
Pz-F3	0.1121	0.0941	3.4558	0.0708
Pz-C3	0.1165	0.0755	21.9359	0.0000
Pz-P3	0.1050	0.0604	14.1295	0.0006
Pz-Fpz1	0.1260	0.0885	5.3108	0.0268
Pz-Fz	0.1158	0.1022	1.6573	0.2058
Pz-Cz	0.1326	0.1006	6.5984	0.0143
Pz-Pz				
Pz-Oz	0.1142	0.0726	18.4893	0.0001
Oz-Fp2	0.1170	0.0794	5.5035	0.0243
Oz-F8	0.1195	0.0855	4.3011	0.0449
Oz-T4	0.1210	0.0855	3.9777	0.0533
Oz-T6	0.1314	0.0940	4.2685	0.0457
Oz-O2	0.0966	0.0955	0.0085	0.9271
Oz-Fp1	0.1136	0.0729	7.1947	0.0108
Oz-F7	0.1097	0.0851	1.7918	0.1887
Oz-T3	0.1052	0.0681	7.4875	0.0094
Oz-T5	0.1077	0.0775	7.9661	0.0075
Oz-O1	0.0879	0.1046	-3.0886	0.0869
Oz-F4	0.1230	0.0863	8.5615	0.0058
Oz-C4	0.1166	0.0798	10.9792	0.0020
Oz-P4	0.1221	0.0762	12.6148	0.0010
Oz-F3	0.1173	0.0814	9.9181	0.0032
Oz-C3	0.1142	0.0736	20.5158	0.0001
Oz-P3	0.1151	0.0686	18.1036	0.0001
Oz-Fpz1	0.1190	0.0779	6.6377	0.0140
Oz-Fz	0.1213	0.0867	6.8689	0.0125
Oz-Cz	0.1144	0.0793	16.9214	0.0002
Oz-Pz	0.1142	0.0726	18.4893	0.0001
Oz-Oz				

Supplementary Table S16. Group means, signed F-values and probabilities of region-specific REM sleep EEG Broadband-2 (0.5-30 Hz) WPLI differences of Williams syndrome and typically developing subjects in all the possible electrode pairings. Color codes: Red = WS > TD (B-H corrected), Yellow = WS > TD (uncorrected), White = WS ≈ typically developing, Light blue = WS < TD (uncorrected), Blue = WS < TD (B-H corrected).

Broadband-2	Mean WS	Mean TD	Signed F	p
Fp2-Fp2				
Fp2-F8	0.1224	0.1304	-0.4116	0.5250
Fp2-T4	0.0769	0.0663	2.5147	0.1211
Fp2-T6	0.0851	0.0672	4.9921	0.0314
Fp2-O2	0.0825	0.0756	0.8001	0.3767
Fp2-Fp1	0.0734	0.0528	3.4353	0.0716
Fp2-F7	0.0914	0.0834	0.3622	0.5508
Fp2-T3	0.0822	0.0609	3.8543	0.0570
Fp2-T5	0.0716	0.0708	0.0114	0.9154
Fp2-O1	0.0691	0.0636	0.8665	0.3578
Fp2-F4	0.1218	0.0812	14.1928	0.0006
Fp2-C4	0.1001	0.0839	3.3838	0.0737
Fp2-P4	0.0846	0.0775	0.7482	0.3925
Fp2-F3	0.0878	0.0623	11.0570	0.0020
Fp2-C3	0.0887	0.0816	0.7077	0.4055
Fp2-P3	0.0798	0.0872	-0.6484	0.4257
Fp2-Fpz1	0.0837	0.0807	0.0797	0.7793
Fp2-Fz	0.1015	0.0730	5.9748	0.0193
Fp2-Cz	0.1028	0.0826	5.4899	0.0245
Fp2-Pz	0.0941	0.0859	0.8893	0.3516
Fp2-Oz	0.0795	0.0724	1.0021	0.3231
F8-Fp2	0.1224	0.1304	-0.4116	0.5250
F8-F8				
F8-T4	0.0751	0.0646	2.0242	0.1630
F8-T6	0.0860	0.0644	6.8778	0.0125
F8-O2	0.0749	0.0519	11.3306	0.0018
F8-Fp1	0.0934	0.0882	0.1930	0.6629
F8-F7	0.0735	0.0602	1.8905	0.1772
F8-T3	0.0739	0.0584	4.0615	0.0510
F8-T5	0.0646	0.0508	6.4984	0.0150
F8-O1	0.0651	0.0484	8.4986	0.0059
F8-F4	0.0881	0.0583	14.9199	0.0004
F8-C4	0.0907	0.0617	14.4677	0.0005
F8-P4	0.0788	0.0565	9.9719	0.0031
F8-F3	0.0690	0.0533	5.9579	0.0194
F8-C3	0.0699	0.0546	5.4211	0.0253
F8-P3	0.0645	0.0523	5.9260	0.0197
F8-Fpz1	0.1285	0.1352	-0.2006	0.6568
F8-Fz	0.0751	0.0646	1.8025	0.1874
F8-Cz	0.0849	0.0556	17.2694	0.0002
F8-Pz	0.0731	0.0515	11.6143	0.0016
F8-Oz	0.0747	0.0528	11.1990	0.0019
T4-Fp2	0.0769	0.0663	2.5147	0.1211
T4-F8	0.0751	0.0646	2.0242	0.1630
T4-T4				
T4-T6	0.0866	0.0658	2.9459	0.0942
T4-O2	0.0779	0.0586	5.3959	0.0256
T4-Fp1	0.0806	0.0643	3.1364	0.0846
T4-F7	0.0747	0.0622	2.4884	0.1230
T4-T3	0.0753	0.0586	1.6087	0.2124

Broadband-2	Mean WS	Mean TD	Signed F	p
T4-T5	0.0623	0.0465	7.8811	0.0078
T4-O1	0.0629	0.0516	3.3499	0.0751
T4-F4	0.0661	0.0585	1.6790	0.2029
T4-C4	0.0757	0.0599	3.1307	0.0849
T4-P4	0.0761	0.0621	1.6540	0.2062
T4-F3	0.0671	0.0585	1.9668	0.1689
T4-C3	0.0662	0.0507	3.6577	0.0634
T4-P3	0.0601	0.0503	2.8920	0.0972
T4-Fpz1	0.0833	0.0686	3.9337	0.0546
T4-Fz	0.0715	0.0605	2.6071	0.1147
T4-Cz	0.0665	0.0499	7.6933	0.0085
T4-Pz	0.0691	0.0469	11.3428	0.0017
T4-Oz	0.0728	0.0568	4.2315	0.0466
T6-Fp2	0.0851	0.0672	4.9921	0.0314
T6-F8	0.0860	0.0644	6.8778	0.0125
T6-T4	0.0866	0.0658	2.9459	0.0942
T6-T6				
T6-O2	0.0825	0.0701	2.4210	0.1280
T6-Fp1	0.0818	0.0652	6.1276	0.0179
T6-F7	0.0863	0.0600	8.4514	0.0061
T6-T3	0.0749	0.0621	1.4409	0.2374
T6-T5	0.0616	0.0443	4.6174	0.0381
T6-O1	0.0646	0.0481	3.1886	0.0821
T6-F4	0.0832	0.0677	4.2122	0.0471
T6-C4	0.0776	0.0603	5.9002	0.0200
T6-P4	0.0617	0.0572	0.3534	0.5557
T6-F3	0.0843	0.0662	4.7888	0.0349
T6-C3	0.0746	0.0588	5.7855	0.0211
T6-P3	0.0606	0.0484	2.2920	0.1383
T6-Fpz1	0.0901	0.0750	3.7522	0.0602
T6-Fz	0.0921	0.0745	2.3341	0.1348
T6-Cz	0.0850	0.0709	2.3907	0.1303
T6-Pz	0.0681	0.0496	5.8158	0.0208
T6-Oz	0.0764	0.0609	3.4845	0.0697
O2-Fp2	0.0825	0.0756	0.8001	0.3767
O2-F8	0.0749	0.0519	11.3306	0.0018
O2-T4	0.0779	0.0586	5.3959	0.0256
O2-T6	0.0825	0.0701	2.4210	0.1280
O2-O2				
O2-Fp1	0.0822	0.0769	0.5549	0.4609
O2-F7	0.0697	0.0542	4.6587	0.0373
O2-T3	0.0662	0.0477	11.8876	0.0014
O2-T5	0.0565	0.0424	11.4017	0.0017
O2-O1	0.0516	0.0433	2.5895	0.1159
O2-F4	0.0723	0.0530	11.6548	0.0015
O2-C4	0.0715	0.0558	6.4751	0.0151
O2-P4	0.0738	0.0542	13.9036	0.0006
O2-F3	0.0741	0.0544	11.0594	0.0020
O2-C3	0.0677	0.0477	16.4805	0.0002
O2-P3	0.0617	0.0416	14.3498	0.0005
O2-Fpz1	0.0898	0.0876	0.0675	0.7964
O2-Fz	0.0768	0.0570	7.6536	0.0087
O2-Cz	0.0726	0.0558	7.6390	0.0088
O2-Pz	0.0649	0.0454	16.5056	0.0002
O2-Oz	0.0588	0.0526	1.7340	0.1958
Fp1-Fp2	0.0734	0.0528	3.4353	0.0716

Broadband-2	Mean WS	Mean TD	Signed F	p
Fp1-F8	0.0934	0.0882	0.1930	0.6629
Fp1-T4	0.0806	0.0643	3.1364	0.0846
Fp1-T6	0.0818	0.0652	6.1276	0.0179
Fp1-O2	0.0822	0.0769	0.5549	0.4609
Fp1-Fp1				
Fp1-F7	0.1211	0.1244	-0.0364	0.8497
Fp1-T3	0.0788	0.0584	8.1152	0.0070
Fp1-T5	0.0774	0.0711	0.4635	0.5001
Fp1-O1	0.0785	0.0639	2.8317	0.1006
Fp1-F4	0.0993	0.0685	10.8141	0.0022
Fp1-C4	0.0995	0.0806	4.6245	0.0379
Fp1-P4	0.0870	0.0763	2.1437	0.1514
Fp1-F3	0.1186	0.0801	13.0066	0.0009
Fp1-C3	0.0990	0.0867	1.8523	0.1815
Fp1-P3	0.0877	0.0908	-0.1082	0.7440
Fp1-Fpz1	0.0759	0.0691	0.4258	0.5180
Fp1-Fz	0.1083	0.0744	6.3620	0.0160
Fp1-Cz	0.1125	0.0853	7.5455	0.0091
Fp1-Pz	0.1012	0.0899	1.6040	0.2130
Fp1-Oz	0.0829	0.0731	1.9472	0.1710
F7-Fp2	0.0914	0.0834	0.3622	0.5508
F7-F8	0.0735	0.0602	1.8905	0.1772
F7-T4	0.0747	0.0622	2.4884	0.1230
F7-T6	0.0863	0.0600	8.4514	0.0061
F7-O2	0.0697	0.0542	4.6587	0.0373
F7-Fp1	0.1211	0.1244	-0.0364	0.8497
F7-F7				
F7-T3	0.0746	0.0613	1.0552	0.3108
F7-T5	0.0675	0.0555	3.4807	0.0698
F7-O1	0.0639	0.0527	2.3586	0.1329
F7-F4	0.0833	0.0616	6.8168	0.0129
F7-C4	0.0859	0.0595	10.0227	0.0030
F7-P4	0.0774	0.0574	5.7655	0.0213
F7-F3	0.1003	0.0742	6.7726	0.0131
F7-C3	0.0967	0.0679	7.9269	0.0077
F7-P3	0.0719	0.0620	1.5651	0.2186
F7-Fpz1	0.1183	0.1277	-0.3057	0.5836
F7-Fz	0.0904	0.0690	5.2586	0.0275
F7-Cz	0.0918	0.0601	13.4303	0.0008
F7-Pz	0.0811	0.0587	7.1647	0.0109
F7-Oz	0.0715	0.0589	1.1876	0.2827
T3-Fp2	0.0822	0.0609	3.8543	0.0570
T3-F8	0.0739	0.0584	4.0615	0.0510
T3-T4	0.0753	0.0586	1.6087	0.2124
T3-T6	0.0749	0.0621	1.4409	0.2374
T3-O2	0.0662	0.0477	11.8876	0.0014
T3-Fp1	0.0788	0.0584	8.1152	0.0070
T3-F7	0.0746	0.0613	1.0552	0.3108
T3-T3				
T3-T5	0.0759	0.0516	6.0553	0.0185
T3-O1	0.0762	0.0574	2.2485	0.1420
T3-F4	0.0820	0.0600	6.9166	0.0123
T3-C4	0.0796	0.0584	4.5073	0.0403
T3-P4	0.0726	0.0522	7.4018	0.0098
T3-F3	0.0804	0.0593	5.1457	0.0291
T3-C3	0.0895	0.0578	14.4298	0.0005

Broadband-2	Mean WS	Mean TD	Signed F	p
T3-P3	0.0841	0.0592	7.2239	0.0106
T3-Fpz1	0.0876	0.0635	5.8209	0.0208
T3-Fz	0.0877	0.0668	2.7759	0.1039
T3-Cz	0.0886	0.0648	3.4294	0.0718
T3-Pz	0.0823	0.0518	12.5056	0.0011
T3-Oz	0.0714	0.0492	9.0317	0.0047
T5-Fp2	0.0716	0.0708	0.0114	0.9154
T5-F8	0.0646	0.0508	6.4984	0.0150
T5-T4	0.0623	0.0465	7.8811	0.0078
T5-T6	0.0616	0.0443	4.6174	0.0381
T5-O2	0.0565	0.0424	11.4017	0.0017
T5-Fp1	0.0774	0.0711	0.4635	0.5001
T5-F7	0.0675	0.0555	3.4807	0.0698
T5-T3	0.0759	0.0516	6.0553	0.0185
T5-T5				
T5-O1	0.0689	0.0546	6.1511	0.0177
T5-F4	0.0648	0.0509	10.9869	0.0020
T5-C4	0.0682	0.0475	11.4078	0.0017
T5-P4	0.0606	0.0444	5.4733	0.0247
T5-F3	0.0748	0.0572	6.3072	0.0164
T5-C3	0.0824	0.0558	11.3105	0.0018
T5-P3	0.0743	0.0557	10.4664	0.0025
T5-Fpz1	0.0789	0.0822	-0.1374	0.7129
T5-Fz	0.0698	0.0549	7.0837	0.0113
T5-Cz	0.0739	0.0560	10.4755	0.0025
T5-Pz	0.0691	0.0491	16.6005	0.0002
T5-Oz	0.0672	0.0510	10.9531	0.0021
O1-Fp2	0.0691	0.0636	0.8665	0.3578
O1-F8	0.0651	0.0484	8.4986	0.0059
O1-T4	0.0629	0.0516	3.3499	0.0751
O1-T6	0.0646	0.0481	3.1886	0.0821
O1-O2	0.0516	0.0433	2.5895	0.1159
O1-Fp1	0.0785	0.0639	2.8317	0.1006
O1-F7	0.0639	0.0527	2.3586	0.1329
O1-T3	0.0762	0.0574	2.2485	0.1420
O1-T5	0.0689	0.0546	6.1511	0.0177
O1-O1				
O1-F4	0.0739	0.0555	11.0516	0.0020
O1-C4	0.0717	0.0536	10.4781	0.0025
O1-P4	0.0628	0.0502	2.8063	0.1021
O1-F3	0.0772	0.0520	19.1606	0.0001
O1-C3	0.0762	0.0544	8.2377	0.0067
O1-P3	0.0649	0.0504	6.0022	0.0190
O1-Fpz1	0.0772	0.0741	0.1597	0.6917
O1-Fz	0.0812	0.0580	7.3341	0.0101
O1-Cz	0.0759	0.0597	10.7390	0.0022
O1-Pz	0.0624	0.0484	6.0743	0.0184
O1-Oz	0.0634	0.0606	0.1434	0.7070
F4-Fp2	0.1218	0.0812	14.1928	0.0006
F4-F8	0.0881	0.0583	14.9199	0.0004
F4-T4	0.0661	0.0585	1.6790	0.2029
F4-T6	0.0832	0.0677	4.2122	0.0471
F4-O2	0.0723	0.0530	11.6548	0.0015
F4-Fp1	0.0993	0.0685	10.8141	0.0022
F4-F7	0.0833	0.0616	6.8168	0.0129
F4-T3	0.0820	0.0600	6.9166	0.0123

Broadband-2	Mean WS	Mean TD	Signed F	p
F4-T5	0.0648	0.0509	10.9869	0.0020
F4-O1	0.0739	0.0555	11.0516	0.0020
F4-F4				
F4-C4	0.0872	0.0727	3.8861	0.0560
F4-P4	0.0853	0.0665	7.3832	0.0099
F4-F3	0.0632	0.0414	10.5887	0.0024
F4-C3	0.0739	0.0617	3.5451	0.0674
F4-P3	0.0713	0.0610	3.4237	0.0721
F4-Fpz1	0.1217	0.0815	14.1630	0.0006
F4-Fz	0.0728	0.0615	2.1052	0.1550
F4-Cz	0.0933	0.0637	15.8683	0.0003
F4-Pz	0.0814	0.0610	10.3273	0.0027
F4-Oz	0.0831	0.0583	14.1735	0.0006
C4-Fp2	0.1001	0.0839	3.3838	0.0737
C4-F8	0.0907	0.0617	14.4677	0.0005
C4-T4	0.0757	0.0599	3.1307	0.0849
C4-T6	0.0776	0.0603	5.9002	0.0200
C4-O2	0.0715	0.0558	6.4751	0.0151
C4-Fp1	0.0995	0.0806	4.6245	0.0379
C4-F7	0.0859	0.0595	10.0227	0.0030
C4-T3	0.0796	0.0584	4.5073	0.0403
C4-T5	0.0682	0.0475	11.4078	0.0017
C4-O1	0.0717	0.0536	10.4781	0.0025
C4-F4	0.0872	0.0727	3.8861	0.0560
C4-C4				
C4-P4	0.0803	0.0642	5.5402	0.0239
C4-F3	0.0781	0.0594	8.3650	0.0063
C4-C3	0.0537	0.0390	6.6114	0.0142
C4-P3	0.0651	0.0549	2.5991	0.1152
C4-Fpz1	0.1033	0.0892	1.9748	0.1681
C4-Fz	0.0992	0.0792	3.2786	0.0781
C4-Cz	0.0652	0.0555	2.3707	0.1319
C4-Pz	0.0793	0.0576	7.6287	0.0088
C4-Oz	0.0809	0.0588	9.6151	0.0036
P4-Fp2	0.0846	0.0775	0.7482	0.3925
P4-F8	0.0788	0.0565	9.9719	0.0031
P4-T4	0.0761	0.0621	1.6540	0.2062
P4-T6	0.0617	0.0572	0.3534	0.5557
P4-O2	0.0738	0.0542	13.9036	0.0006
P4-Fp1	0.0870	0.0763	2.1437	0.1514
P4-F7	0.0774	0.0574	5.7655	0.0213
P4-T3	0.0726	0.0522	7.4018	0.0098
P4-T5	0.0606	0.0444	5.4733	0.0247
P4-O1	0.0628	0.0502	2.8063	0.1021
P4-F4	0.0853	0.0665	7.3832	0.0099
P4-C4	0.0803	0.0642	5.5402	0.0239
P4-P4				
P4-F3	0.0803	0.0594	10.3065	0.0027
P4-C3	0.0708	0.0474	29.4208	0.0000
P4-P3	0.0521	0.0414	2.5326	0.1198
P4-Fpz1	0.0927	0.0870	0.4648	0.4995
P4-Fz	0.0902	0.0731	2.3543	0.1332
P4-Cz	0.0883	0.0744	2.3595	0.1328
P4-Pz	0.0655	0.0474	4.3770	0.0432
P4-Oz	0.0806	0.0552	15.4463	0.0003
F3-Fp2	0.0878	0.0623	11.0570	0.0020

Broadband-2	Mean WS	Mean TD	Signed F	p
F3-F8	0.0690	0.0533	5.9579	0.0194
F3-T4	0.0671	0.0585	1.9668	0.1689
F3-T6	0.0843	0.0662	4.7888	0.0349
F3-O2	0.0741	0.0544	11.0594	0.0020
F3-Fp1	0.1186	0.0801	13.0066	0.0009
F3-F7	0.1003	0.0742	6.7726	0.0131
F3-T3	0.0804	0.0593	5.1457	0.0291
F3-T5	0.0748	0.0572	6.3072	0.0164
F3-O1	0.0772	0.0520	19.1606	0.0001
F3-F4	0.0632	0.0414	10.5887	0.0024
F3-C4	0.0781	0.0594	8.3650	0.0063
F3-P4	0.0803	0.0594	10.3065	0.0027
F3-F3				
F3-C3	0.0902	0.0705	5.9223	0.0198
F3-P3	0.0797	0.0679	2.8197	0.1013
F3-Fpz1	0.1108	0.0763	11.4946	0.0016
F3-Fz	0.0749	0.0518	5.7084	0.0220
F3-Cz	0.0961	0.0610	21.1611	0.0000
F3-Pz	0.0854	0.0628	8.5072	0.0059
F3-Oz	0.0828	0.0555	16.6930	0.0002
C3-Fp2	0.0887	0.0816	0.7077	0.4055
C3-F8	0.0699	0.0546	5.4211	0.0253
C3-T4	0.0662	0.0507	3.6577	0.0634
C3-T6	0.0746	0.0588	5.7855	0.0211
C3-O2	0.0677	0.0477	16.4805	0.0002
C3-Fp1	0.0990	0.0867	1.8523	0.1815
C3-F7	0.0967	0.0679	7.9269	0.0077
C3-T3	0.0895	0.0578	14.4298	0.0005
C3-T5	0.0824	0.0558	11.3105	0.0018
C3-O1	0.0762	0.0544	8.2377	0.0067
C3-F4	0.0739	0.0617	3.5451	0.0674
C3-C4	0.0537	0.0390	6.6114	0.0142
C3-P4	0.0708	0.0474	29.4208	0.0000
C3-F3	0.0902	0.0705	5.9223	0.0198
C3-C3				
C3-P3	0.0822	0.0655	4.2661	0.0457
C3-Fpz1	0.0988	0.0917	0.5466	0.4642
C3-Fz	0.0939	0.0774	2.1765	0.1484
C3-Cz	0.0700	0.0568	2.1929	0.1469
C3-Pz	0.0832	0.0548	11.6048	0.0016
C3-Oz	0.0798	0.0527	16.9222	0.0002
P3-Fp2	0.0798	0.0872	-0.6484	0.4257
P3-F8	0.0645	0.0523	5.9260	0.0197
P3-T4	0.0601	0.0503	2.8920	0.0972
P3-T6	0.0606	0.0484	2.2920	0.1383
P3-O2	0.0617	0.0416	14.3498	0.0005
P3-Fp1	0.0877	0.0908	-0.1082	0.7440
P3-F7	0.0719	0.0620	1.5651	0.2186
P3-T3	0.0841	0.0592	7.2239	0.0106
P3-T5	0.0743	0.0557	10.4664	0.0025
P3-O1	0.0649	0.0504	6.0022	0.0190
P3-F4	0.0713	0.0610	3.4237	0.0721
P3-C4	0.0651	0.0549	2.5991	0.1152
P3-P4	0.0521	0.0414	2.5326	0.1198
P3-F3	0.0797	0.0679	2.8197	0.1013
P3-C3	0.0822	0.0655	4.2661	0.0457

Broadband-2	Mean WS	Mean TD	Signed F	p
P3-P3				
P3-Fpz1	0.0904	0.1008	-1.0158	0.3199
P3-Fz	0.0776	0.0682	1.5605	0.2192
P3-Cz	0.0795	0.0721	1.2738	0.2661
P3-Pz	0.0639	0.0447	11.2354	0.0018
P3-Oz	0.0747	0.0500	16.0840	0.0003
Fpz1-Fp2	0.0837	0.0807	0.0797	0.7793
Fpz1-F8	0.1285	0.1352	-0.2006	0.6568
Fpz1-T4	0.0833	0.0686	3.9337	0.0546
Fpz1-T6	0.0901	0.0750	3.7522	0.0602
Fpz1-O2	0.0898	0.0876	0.0675	0.7964
Fpz1-Fp1	0.0759	0.0691	0.4258	0.5180
Fpz1-F7	0.1183	0.1277	-0.3057	0.5836
Fpz1-T3	0.0876	0.0635	5.8209	0.0208
Fpz1-T5	0.0789	0.0822	-0.1374	0.7129
Fpz1-O1	0.0772	0.0741	0.1597	0.6917
Fpz1-F4	0.1217	0.0815	14.1630	0.0006
Fpz1-C4	0.1033	0.0892	1.9748	0.1681
Fpz1-P4	0.0927	0.0870	0.4648	0.4995
Fpz1-F3	0.1108	0.0763	11.4946	0.0016
Fpz1-C3	0.0988	0.0917	0.5466	0.4642
Fpz1-P3	0.0904	0.1008	-1.0158	0.3199
Fpz1-Fpz1				
Fpz1-Fz	0.1218	0.0851	7.3017	0.0102
Fpz1-Cz	0.1144	0.0941	4.1198	0.0494
Fpz1-Pz	0.1058	0.1013	0.1994	0.6577
Fpz1-Oz	0.0874	0.0846	0.1253	0.7253
Fz-Fp2	0.1015	0.0730	5.9748	0.0193
Fz-F8	0.0751	0.0646	1.8025	0.1874
Fz-T4	0.0715	0.0605	2.6071	0.1147
Fz-T6	0.0921	0.0745	2.3341	0.1348
Fz-O2	0.0768	0.0570	7.6536	0.0087
Fz-Fp1	0.1083	0.0744	6.3620	0.0160
Fz-F7	0.0904	0.0690	5.2586	0.0275
Fz-T3	0.0877	0.0668	2.7759	0.1039
Fz-T5	0.0698	0.0549	7.0837	0.0113
Fz-O1	0.0812	0.0580	7.3341	0.0101
Fz-F4	0.0728	0.0615	2.1052	0.1550
Fz-C4	0.0992	0.0792	3.2786	0.0781
Fz-P4	0.0902	0.0731	2.3543	0.1332
Fz-F3	0.0749	0.0518	5.7084	0.0220
Fz-C3	0.0939	0.0774	2.1765	0.1484
Fz-P3	0.0776	0.0682	1.5605	0.2192
Fz-Fpz1	0.1218	0.0851	7.3017	0.0102
Fz-Fz				
Fz-Cz	0.1215	0.0780	16.1094	0.0003
Fz-Pz	0.0859	0.0665	6.8185	0.0128
Fz-Oz	0.0875	0.0613	8.2868	0.0065
Cz-Fp2	0.1028	0.0826	5.4899	0.0245
Cz-F8	0.0849	0.0556	17.2694	0.0002
Cz-T4	0.0665	0.0499	7.6933	0.0085
Cz-T6	0.0850	0.0709	2.3907	0.1303
Cz-O2	0.0726	0.0558	7.6390	0.0088
Cz-Fp1	0.1125	0.0853	7.5455	0.0091
Cz-F7	0.0918	0.0601	13.4303	0.0008
Cz-T3	0.0886	0.0648	3.4294	0.0718

Broadband-2	Mean WS	Mean TD	Signed F	p
Cz-T5	0.0739	0.0560	10.4755	0.0025
Cz-O1	0.0759	0.0597	10.7390	0.0022
Cz-F4	0.0933	0.0637	15.8683	0.0003
Cz-C4	0.0652	0.0555	2.3707	0.1319
Cz-P4	0.0883	0.0744	2.3595	0.1328
Cz-F3	0.0961	0.0610	21.1611	0.0000
Cz-C3	0.0700	0.0568	2.1929	0.1469
Cz-P3	0.0795	0.0721	1.2738	0.2661
Cz-Fpz1	0.1144	0.0941	4.1198	0.0494
Cz-Fz	0.1215	0.0780	16.1094	0.0003
Cz-Cz				
Cz-Pz	0.0922	0.0725	4.9449	0.0322
Cz-Oz	0.0859	0.0656	7.1281	0.0111
Pz-Fp2	0.0941	0.0859	0.8893	0.3516
Pz-F8	0.0731	0.0515	11.6143	0.0016
Pz-T4	0.0691	0.0469	11.3428	0.0017
Pz-T6	0.0681	0.0496	5.8158	0.0208
Pz-O2	0.0649	0.0454	16.5056	0.0002
Pz-Fp1	0.1012	0.0899	1.6040	0.2130
Pz-F7	0.0811	0.0587	7.1647	0.0109
Pz-T3	0.0823	0.0518	12.5056	0.0011
Pz-T5	0.0691	0.0491	16.6005	0.0002
Pz-O1	0.0624	0.0484	6.0743	0.0184
Pz-F4	0.0814	0.0610	10.3273	0.0027
Pz-C4	0.0793	0.0576	7.6287	0.0088
Pz-P4	0.0655	0.0474	4.3770	0.0432
Pz-F3	0.0854	0.0628	8.5072	0.0059
Pz-C3	0.0832	0.0548	11.6048	0.0016
Pz-P3	0.0639	0.0447	11.2354	0.0018
Pz-Fpz1	0.1058	0.1013	0.1994	0.6577
Pz-Fz	0.0859	0.0665	6.8185	0.0128
Pz-Cz	0.0922	0.0725	4.9449	0.0322
Pz-Pz				
Pz-Oz	0.0780	0.0521	13.1478	0.0008
Oz-Fp2	0.0795	0.0724	1.0021	0.3231
Oz-F8	0.0747	0.0528	11.1990	0.0019
Oz-T4	0.0728	0.0568	4.2315	0.0466
Oz-T6	0.0764	0.0609	3.4845	0.0697
Oz-O2	0.0588	0.0526	1.7340	0.1958
Oz-Fp1	0.0829	0.0731	1.9472	0.1710
Oz-F7	0.0715	0.0589	1.1876	0.2827
Oz-T3	0.0714	0.0492	9.0317	0.0047
Oz-T5	0.0672	0.0510	10.9531	0.0021
Oz-O1	0.0634	0.0606	0.1434	0.7070
Oz-F4	0.0831	0.0583	14.1735	0.0006
Oz-C4	0.0809	0.0588	9.6151	0.0036
Oz-P4	0.0806	0.0552	15.4463	0.0003
Oz-F3	0.0828	0.0555	16.6930	0.0002
Oz-C3	0.0798	0.0527	16.9222	0.0002
Oz-P3	0.0747	0.0500	16.0840	0.0003
Oz-Fpz1	0.0874	0.0846	0.1253	0.7253
Oz-Fz	0.0875	0.0613	8.2868	0.0065
Oz-Cz	0.0859	0.0656	7.1281	0.0111
Oz-Pz	0.0780	0.0521	13.1478	0.0008
Oz-Oz				

Supplementary Table S17. Signed F-values and probabilities of region-specific and band-limited REM sleep EEG WPLI differences between Williams syndrome and typically developing subjects (intra- and inter-regional pairings). Color codes: Red = WS > TD (B-H corrected), Yellow = WS > TD (uncorrected), White = WS ≈ typically developing, Light blue = WS < TD (uncorrected), Blue = WS < TD (B-H corrected).

**REM**		PF/AC		LPF		C		T		P/IP		O	
		Sign. F	p	Sign. F	p	Sign. F	p	Sign. F	p	Sign. F	p	Sign. F	p
**Slow oscillation**	PF/AC	0.901	0.348	0.131	0.719	0.833	0.367	0.029	0.866	0.130	0.720	1.688	0.202
	LPF	0.131	0.719	-0.742	0.394	-0.437	0.513	-3.570	0.066	-0.474	0.495	0.075	0.786
	C	0.833	0.367	-0.437	0.513	-0.113	0.739	-0.381	0.541	0.125	0.725	0.418	0.522
	T	0.029	0.866	-3.570	0.066	-0.381	0.541	-1.203	0.280	-0.223	0.639	-0.534	0.469
	P/IP	0.130	0.720	-0.474	0.495	0.125	0.725	-0.223	0.639	0.431	0.516	0.145	0.705
	O	1.688	0.202	0.075	0.786	0.418	0.522	-0.534	0.469	0.145	0.705	0.661	0.421
**Delta**	PF/AC	11.267	0.002	18.380	0.000	0.784	0.382	0.937	0.339	-1.364	0.250	-1.100	0.301
	LPF	18.380	0.000	9.368	0.004	0.009	0.926	0.207	0.652	-2.413	0.129	-2.145	0.151
	C	0.784	0.382	0.009	0.926	-0.035	0.852	5.723	0.022	0.303	0.585	-1.833	0.184
	T	0.937	0.339	0.207	0.652	5.723	0.022	4.306	0.045	0.069	0.794	-0.286	0.596
	P/IP	-1.364	0.250	-2.413	0.129	0.303	0.585	0.069	0.794	2.350	0.134	1.533	0.223
	O	-1.100	0.301	-2.145	0.151	-1.833	0.184	-0.286	0.596	1.533	0.223	3.370	0.074
**Theta**	PF/AC	8.896	0.005	4.994	0.031	0.720	0.401	0.059	0.810	0.045	0.834	0.615	0.438
	LPF	4.994	0.031	10.405	0.003	0.170	0.683	0.514	0.478	0.107	0.746	0.378	0.542
	C	0.720	0.401	0.170	0.683	-4.515	0.040	2.528	0.120	11.221	0.002	6.937	0.012
	T	0.059	0.810	0.514	0.478	2.528	0.120	4.817	0.034	0.689	0.412	1.292	0.263
	P/IP	0.045	0.834	0.107	0.746	11.221	0.002	0.689	0.412	6.575	0.014	11.130	0.002
	O	0.615	0.438	0.378	0.542	6.937	0.012	1.292	0.263	11.130	0.002	2.535	0.120
**Alpha**	PF/AC	4.145	0.049	1.856	0.181	0.237	0.629	0.418	0.522	-0.645	0.427	-0.124	0.727
	LPF	1.856	0.181	7.793	0.008	-0.071	0.792	0.708	0.405	0.035	0.852	0.209	0.650
	C	0.237	0.629	-0.071	0.792	-1.644	0.208	5.152	0.029	3.266	0.079	4.306	0.045
	T	0.418	0.522	0.708	0.405	5.152	0.029	6.864	0.013	6.369	0.016	3.064	0.088
	P/IP	-0.645	0.427	0.035	0.852	3.266	0.079	6.369	0.016	0.011	0.918	0.269	0.607
	O	-0.124	0.727	0.209	0.650	4.306	0.045	3.064	0.088	0.269	0.607	-10.579	0.002
**Low sigma**	PF/AC	5.621	0.023	4.160	0.048	6.039	0.019	10.626	0.002	8.529	0.006	5.602	0.023
	LPF	4.160	0.048	8.481	0.006	6.071	0.018	7.143	0.011	7.210	0.011	6.997	0.012
	C	6.039	0.019	6.071	0.018	0.154	0.697	10.864	0.002	3.214	0.081	2.975	0.093
	T	10.626	0.002	7.143	0.011	10.864	0.002	6.411	0.016	14.523	0.000	4.365	0.043
	P/IP	8.529	0.006	7.210	0.011	3.214	0.081	14.523	0.000	8.238	0.007	0.092	0.763
	O	5.602	0.023	6.997	0.012	2.975	0.093	4.365	0.043	0.092	0.763	-7.366	0.010
**High sigma**	PF/AC	13.784	0.001	12.470	0.001	9.671	0.004	9.914	0.003	3.042	0.089	3.569	0.067
	LPF	12.470	0.001	12.491	0.001	7.260	0.010	7.315	0.010	2.756	0.105	2.215	0.145
	C	9.671	0.004	7.260	0.010	12.652	0.001	16.791	0.000	4.230	0.047	1.865	0.180
	T	9.914	0.003	7.315	0.010	16.791	0.000	5.375	0.026	20.373	0.000	4.445	0.042
	P/IP	3.042	0.089	2.756	0.105	4.230	0.047	20.373	0.000	13.285	0.001	2.255	0.141
	O	3.569	0.067	2.215	0.145	1.865	0.180	4.445	0.042	2.255	0.141	-0.987	0.327
**Beta**	PF/AC	8.861	0.005	8.271	0.007	9.023	0.005	5.638	0.023	9.156	0.004	7.742	0.008
	LPF	8.271	0.007	14.109	0.001	15.817	0.000	7.596	0.009	13.451	0.001	7.940	0.008
	C	9.023	0.005	15.817	0.000	16.274	0.000	12.189	0.001	15.532	0.000	12.462	0.001
	T	5.638	0.023	7.596	0.009	12.189	0.001	5.110	0.030	12.523	0.001	7.036	0.012
	P/IP	9.156	0.004	13.451	0.001	15.532	0.000	12.523	0.001	18.574	0.000	12.309	0.001
	O	7.742	0.008	7.940	0.008	12.462	0.001	7.036	0.012	12.309	0.001	0.963	0.333
**Low gamma**	PF/AC	0.005	0.946	-0.816	0.372	2.313	0.137	0.054	0.818	3.596	0.066	3.666	0.063
	LPF	-0.816	0.372	1.138	0.293	8.811	0.005	1.309	0.260	7.363	0.010	7.831	0.008
	C	2.313	0.137	8.811	0.005	3.269	0.079	3.551	0.067	5.002	0.031	8.763	0.005
	T	0.054	0.818	1.309	0.260	3.551	0.067	0.285	0.597	4.682	0.037	5.685	0.022
	P/IP	3.596	0.066	7.363	0.010	5.002	0.031	4.682	0.037	2.873	0.098	5.722	0.022
	O	3.666	0.063	7.831	0.008	8.763	0.005	5.685	0.022	5.722	0.022	3.283	0.078
**High**	PF/AC	0.059	0.809	0.096	0.759	-1.060	0.310	1.210	0.278	-1.023	0.318	-0.436	0.513
	LPF	0.096	0.759	0.021	0.884	0.186	0.669	0.185	0.670	0.408	0.527	1.918	0.174

C	-1.060	0.310	0.186	0.669	0.351	0.557	0.019	0.892	0.095	0.759	0.724	0.400
T	1.210	0.278	0.185	0.670	0.019	0.892	0.010	0.921	0.115	0.737	0.398	0.532
P/IP	-1.023	0.318	0.408	0.527	0.095	0.759	0.115	0.737	0.213	0.647	0.580	0.451
O	-0.436	0.513	1.918	0.174	0.724	0.400	0.398	0.532	0.580	0.451	1.087	0.304

Supplementary Table S18. Group means, signed F-values and probabilities of region-specific REM sleep EEG theta WPLI differences of Williams syndrome and typically developing subjects in all the possible electrode pairings. Color codes: Red = WS > TD (B-H corrected), Yellow = WS > TD (uncorrected), White = WS ≈ typically developing, Light blue = WS < TD (uncorrected), Blue = WS < TD (B-H corrected).

Theta	Mean WS	Mean TD	Signed F	p
Fp2-Fp2				
Fp2-F8	0.1341	0.1260	0.0879	0.7684
Fp2-T4	0.1370	0.1347	0.0081	0.9286
Fp2-T6	0.1308	0.1190	0.3099	0.5810
Fp2-O2	0.1157	0.1086	0.1647	0.6871
Fp2-Fp1	0.0610	0.0432	2.8206	0.1013
Fp2-F7	0.0975	0.0686	2.9083	0.0963
Fp2-T3	0.1065	0.0976	0.2229	0.6395
Fp2-T5	0.1095	0.0946	0.5862	0.4486
Fp2-O1	0.1120	0.0996	0.4760	0.4944
Fp2-F4	0.2857	0.1896	6.6377	0.0140
Fp2-C4	0.2318	0.2031	0.6821	0.4140
Fp2-P4	0.1509	0.1485	0.0087	0.9264
Fp2-F3	0.2092	0.1241	7.6372	0.0088
Fp2-C3	0.1983	0.1752	0.5775	0.4520
Fp2-P3	0.1333	0.1301	0.0175	0.8955
Fp2-Fpz1	0.1138	0.1077	0.1035	0.7494
Fp2-Fz	0.2418	0.1326	11.8742	0.0014
Fp2-Cz	0.2222	0.1724	2.4528	0.1256
Fp2-Pz	0.1524	0.1389	0.2515	0.6189
Fp2-Oz	0.1219	0.1019	1.0729	0.3068
F8-Fp2	0.1341	0.1260	0.0879	0.7684
F8-F8				
F8-T4	0.1262	0.1319	-0.0578	0.8113
F8-T6	0.1300	0.1265	0.0306	0.8621
F8-O2	0.1195	0.1124	0.1772	0.6761
F8-Fp1	0.0857	0.0891	-0.0448	0.8335
F8-F7	0.0647	0.0448	2.9740	0.0927
F8-T3	0.0837	0.0770	0.2163	0.6445
F8-T5	0.1121	0.0965	0.9056	0.3473
F8-O1	0.1126	0.1033	0.3000	0.5871
F8-F4	0.1838	0.1328	3.0726	0.0877
F8-C4	0.1894	0.1961	-0.0427	0.8374
F8-P4	0.1414	0.1484	-0.1000	0.7535
F8-F3	0.1253	0.0788	5.0543	0.0304
F8-C3	0.1430	0.1469	-0.0231	0.8800
F8-P3	0.1187	0.1247	-0.0868	0.7699
F8-Fpz1	0.1329	0.1351	-0.0077	0.9304
F8-Fz	0.1380	0.0967	3.3812	0.0738
F8-Cz	0.1669	0.1487	0.3781	0.5423
F8-Pz	0.1334	0.1303	0.0204	0.8873
F8-Oz	0.1105	0.0993	0.3865	0.5379
T4-Fp2	0.1370	0.1347	0.0081	0.9286
T4-F8	0.1262	0.1319	-0.0578	0.8113
T4-T4				
T4-T6	0.1554	0.1379	0.6046	0.4417
T4-O2	0.1506	0.1184	2.0405	0.1613

Theta	Mean WS	Mean TD	Signed F	p
T4-Fp1	0.1194	0.1263	-0.1235	0.7272
T4-F7	0.1029	0.1019	0.0050	0.9438
T4-T3	0.0770	0.0494	4.8167	0.0344
T4-T5	0.0939	0.0704	2.9954	0.0916
T4-O1	0.1231	0.1037	1.0411	0.3140
T4-F4	0.1373	0.1132	0.7753	0.3841
T4-C4	0.1815	0.1407	1.2762	0.2657
T4-P4	0.1539	0.1427	0.1428	0.7076
T4-F3	0.1105	0.1073	0.0270	0.8704
T4-C3	0.1243	0.0931	1.8091	0.1866
T4-P3	0.1077	0.0990	0.1869	0.6679
T4-Fpz1	0.1448	0.1477	-0.0138	0.9070
T4-Fz	0.1276	0.1152	0.3215	0.5741
T4-Cz	0.1506	0.1111	2.0460	0.1608
T4-Pz	0.1280	0.1082	0.6432	0.4276
T4-Oz	0.1256	0.1091	0.5424	0.4660
T6-Fp2	0.1308	0.1190	0.3099	0.5810
T6-F8	0.1300	0.1265	0.0306	0.8621
T6-T4	0.1554	0.1379	0.6046	0.4417
T6-T6				
T6-O2	0.1638	0.1052	6.1874	0.0174
T6-Fp1	0.1278	0.1187	0.2042	0.6540
T6-F7	0.1226	0.1190	0.0376	0.8474
T6-T3	0.1116	0.1012	0.3600	0.5521
T6-T5	0.0736	0.0483	3.3164	0.0765
T6-O1	0.0941	0.0789	0.8740	0.3558
T6-F4	0.1222	0.1019	1.1007	0.3007
T6-C4	0.1784	0.0817	19.7345	0.0001
T6-P4	0.1364	0.0826	5.6261	0.0229
T6-F3	0.1161	0.1156	0.0007	0.9794
T6-C3	0.1341	0.0687	12.3890	0.0011
T6-P3	0.1044	0.0544	12.5383	0.0011
T6-Fpz1	0.1416	0.1302	0.2539	0.6173
T6-Fz	0.1141	0.1191	-0.0637	0.8021
T6-Cz	0.1510	0.0929	7.0347	0.0116
T6-Pz	0.1281	0.0771	5.8109	0.0209
T6-Oz	0.1302	0.1086	0.8759	0.3552
O2-Fp2	0.1157	0.1086	0.1647	0.6871
O2-F8	0.1195	0.1124	0.1772	0.6761
O2-T4	0.1506	0.1184	2.0405	0.1613
O2-T6	0.1638	0.1052	6.1874	0.0174
O2-O2				
O2-Fp1	0.1139	0.1065	0.1831	0.6712
O2-F7	0.1123	0.1065	0.1110	0.7408
O2-T3	0.1199	0.1093	0.2677	0.6079
O2-T5	0.1096	0.0830	1.7854	0.1894
O2-O1	0.0834	0.0614	1.7425	0.1947
O2-F4	0.1171	0.1204	-0.0319	0.8591
O2-C4	0.1450	0.0873	10.0637	0.0030
O2-P4	0.1369	0.0719	10.0204	0.0030
O2-F3	0.1146	0.1223	-0.1561	0.6950
O2-C3	0.1165	0.0888	2.3563	0.1331
O2-P3	0.1128	0.0707	5.1705	0.0287

Theta	Mean WS	Mean TD	Signed F	p
O2-Fpz1	0.1230	0.1105	0.4482	0.5072
O2-Fz	0.1181	0.1307	-0.3782	0.5422
O2-Cz	0.1177	0.0849	3.0356	0.0895
O2-Pz	0.1316	0.0775	7.2956	0.0103
O2-Oz	0.1415	0.1117	1.1531	0.2897
Fp1-Fp2	0.0610	0.0432	2.8206	0.1013
Fp1-F8	0.0857	0.0891	-0.0448	0.8335
Fp1-T4	0.1194	0.1263	-0.1235	0.7272
Fp1-T6	0.1278	0.1187	0.2042	0.6540
Fp1-O2	0.1139	0.1065	0.1831	0.6712
Fp1-Fp1				
Fp1-F7	0.1210	0.1114	0.1941	0.6621
Fp1-T3	0.1156	0.1163	-0.0010	0.9751
Fp1-T5	0.1096	0.0949	0.4712	0.4966
Fp1-O1	0.1097	0.0921	1.0451	0.3131
Fp1-F4	0.2003	0.1267	6.5355	0.0147
Fp1-C4	0.2028	0.1774	0.7049	0.4064
Fp1-P4	0.1447	0.1390	0.0558	0.8145
Fp1-F3	0.2664	0.1784	5.5581	0.0236
Fp1-C3	0.2176	0.1937	0.5229	0.4740
Fp1-P3	0.1364	0.1326	0.0206	0.8866
Fp1-Fpz1	0.1059	0.0930	0.3275	0.5705
Fp1-Fz	0.2329	0.1310	12.1976	0.0012
Fp1-Cz	0.2167	0.1671	2.4364	0.1268
Fp1-Pz	0.1525	0.1363	0.3689	0.5472
Fp1-Oz	0.1209	0.0963	1.8433	0.1826
F7-Fp2	0.0975	0.0686	2.9083	0.0963
F7-F8	0.0647	0.0448	2.9740	0.0927
F7-T4	0.1029	0.1019	0.0050	0.9438
F7-T6	0.1226	0.1190	0.0376	0.8474
F7-O2	0.1123	0.1065	0.1110	0.7408
F7-Fp1	0.1210	0.1114	0.1941	0.6621
F7-F7				
F7-T3	0.1140	0.1219	-0.1180	0.7331
F7-T5	0.1181	0.0968	0.9515	0.3355
F7-O1	0.1128	0.0928	1.4930	0.2293
F7-F4	0.1172	0.0743	6.4330	0.0154
F7-C4	0.1602	0.1410	0.6289	0.4327
F7-P4	0.1331	0.1266	0.1015	0.7518
F7-F3	0.1999	0.1175	8.1429	0.0070
F7-C3	0.2048	0.1804	0.5646	0.4571
F7-P3	0.1409	0.1291	0.2451	0.6234
F7-Fpz1	0.1261	0.1175	0.1539	0.6970
F7-Fz	0.1378	0.0890	5.9295	0.0197
F7-Cz	0.1823	0.1315	2.9893	0.0919
F7-Pz	0.1456	0.1242	0.8969	0.3496
F7-Oz	0.1140	0.0934	1.6477	0.2070
T3-Fp2	0.1065	0.0976	0.2229	0.6395
T3-F8	0.0837	0.0770	0.2163	0.6445
T3-T4	0.0770	0.0494	4.8167	0.0344
T3-T6	0.1116	0.1012	0.3600	0.5521
T3-O2	0.1199	0.1093	0.2677	0.6079
T3-Fp1	0.1156	0.1163	-0.0010	0.9751

Theta	Mean WS	Mean TD	Signed F	p
T3-F7	0.1140	0.1219	-0.1180	0.7331
T3-T3				
T3-T5	0.1383	0.1188	0.6652	0.4198
T3-O1	0.1402	0.1082	2.0078	0.1646
T3-F4	0.1003	0.0759	1.8820	0.1782
T3-C4	0.1339	0.0964	2.4012	0.1295
T3-P4	0.1228	0.1148	0.1234	0.7273
T3-F3	0.1569	0.1134	2.3049	0.1372
T3-C3	0.2199	0.1698	1.5332	0.2232
T3-P3	0.1700	0.1608	0.0816	0.7767
T3-Fpz1	0.1196	0.1228	-0.0184	0.8927
T3-Fz	0.1266	0.1018	1.0668	0.3082
T3-Cz	0.1740	0.1078	4.2266	0.0467
T3-Pz	0.1516	0.1167	1.4823	0.2309
T3-Oz	0.1248	0.1024	1.0354	0.3153
T5-Fp2	0.1095	0.0946	0.5862	0.4486
T5-F8	0.1121	0.0965	0.9056	0.3473
T5-T4	0.0939	0.0704	2.9954	0.0916
T5-T6	0.0736	0.0483	3.3164	0.0765
T5-O2	0.1096	0.0830	1.7854	0.1894
T5-Fp1	0.1096	0.0949	0.4712	0.4966
T5-F7	0.1181	0.0968	0.9515	0.3355
T5-T3	0.1383	0.1188	0.6652	0.4198
T5-T5				
T5-O1	0.1523	0.1016	4.7327	0.0359
T5-F4	0.1122	0.0825	2.7252	0.1070
T5-C4	0.1285	0.0642	11.2493	0.0018
T5-P4	0.0791	0.0695	0.3072	0.5827
T5-F3	0.1398	0.0876	6.3461	0.0161
T5-C3	0.2040	0.0942	19.7065	0.0001
T5-P3	0.1614	0.1189	2.4566	0.1253
T5-Fpz1	0.1138	0.1027	0.2478	0.6215
T5-Fz	0.1202	0.0889	2.4010	0.1295
T5-Cz	0.1667	0.0865	10.0896	0.0030
T5-Pz	0.1357	0.0966	2.3197	0.1360
T5-Oz	0.1209	0.1016	0.7623	0.3881
O1-Fp2	0.1120	0.0996	0.4760	0.4944
O1-F8	0.1126	0.1033	0.3000	0.5871
O1-T4	0.1231	0.1037	1.0411	0.3140
O1-T6	0.0941	0.0789	0.8740	0.3558
O1-O2	0.0834	0.0614	1.7425	0.1947
O1-Fp1	0.1097	0.0921	1.0451	0.3131
O1-F7	0.1128	0.0928	1.4930	0.2293
O1-T3	0.1402	0.1082	2.0078	0.1646
O1-T5	0.1523	0.1016	4.7327	0.0359
O1-O1				
O1-F4	0.1180	0.1073	0.3491	0.5581
O1-C4	0.1254	0.0745	7.3436	0.0100
O1-P4	0.0974	0.0598	5.3933	0.0257
O1-F3	0.1201	0.0949	2.3732	0.1317
O1-C3	0.1489	0.0751	17.0678	0.0002
O1-P3	0.1561	0.0776	15.8793	0.0003
O1-Fpz1	0.1174	0.0988	1.0039	0.3227

Theta	Mean WS	Mean TD	Signed F	p
O1-Fz	0.1214	0.1090	0.4455	0.5085
O1-Cz	0.1278	0.0742	9.1245	0.0045
O1-Pz	0.1322	0.0718	10.1689	0.0029
O1-Oz	0.1149	0.0983	0.7996	0.3768
F4-Fp2	0.2857	0.1896	6.6377	0.0140
F4-F8	0.1838	0.1328	3.0726	0.0877
F4-T4	0.1373	0.1132	0.7753	0.3841
F4-T6	0.1222	0.1019	1.1007	0.3007
F4-O2	0.1171	0.1204	-0.0319	0.8591
F4-Fp1	0.2003	0.1267	6.5355	0.0147
F4-F7	0.1172	0.0743	6.4330	0.0154
F4-T3	0.1003	0.0759	1.8820	0.1782
F4-T5	0.1122	0.0825	2.7252	0.1070
F4-O1	0.1180	0.1073	0.3491	0.5581
F4-F4				
F4-C4	0.2188	0.2323	-0.1589	0.6924
F4-P4	0.1398	0.1550	-0.4301	0.5159
F4-F3	0.0930	0.0549	5.5039	0.0243
F4-C3	0.1680	0.1842	-0.3564	0.5540
F4-P3	0.1250	0.1334	-0.1434	0.7070
F4-Fpz1	0.2862	0.2023	4.9264	0.0325
F4-Fz	0.1387	0.1122	0.9026	0.3481
F4-Cz	0.2307	0.1838	2.1473	0.1510
F4-Pz	0.1465	0.1577	-0.1874	0.6675
F4-Oz	0.1239	0.1168	0.1085	0.7437
C4-Fp2	0.2318	0.2031	0.6821	0.4140
C4-F8	0.1894	0.1961	-0.0427	0.8374
C4-T4	0.1815	0.1407	1.2762	0.2657
C4-T6	0.1784	0.0817	19.7345	0.0001
C4-O2	0.1450	0.0873	10.0637	0.0030
C4-Fp1	0.2028	0.1774	0.7049	0.4064
C4-F7	0.1602	0.1410	0.6289	0.4327
C4-T3	0.1339	0.0964	2.4012	0.1295
C4-T5	0.1285	0.0642	11.2493	0.0018
C4-O1	0.1254	0.0745	7.3436	0.0100
C4-F4	0.2188	0.2323	-0.1589	0.6924
C4-C4				
C4-P4	0.1823	0.1044	10.5027	0.0025
C4-F3	0.1681	0.1912	-0.7560	0.3900
C4-C3	0.0671	0.0593	0.4621	0.5008
C4-P3	0.1153	0.0769	4.1127	0.0496
C4-Fpz1	0.2409	0.2089	0.7729	0.3848
C4-Fz	0.2355	0.2599	-0.5398	0.4670
C4-Cz	0.1156	0.1725	-5.7827	0.0212
C4-Pz	0.1302	0.0875	3.5921	0.0657
C4-Oz	0.1287	0.0891	3.4354	0.0716
P4-Fp2	0.1509	0.1485	0.0087	0.9264
P4-F8	0.1414	0.1484	-0.1000	0.7535
P4-T4	0.1539	0.1427	0.1428	0.7076
P4-T6	0.1364	0.0826	5.6261	0.0229
P4-O2	0.1369	0.0719	10.0204	0.0030
P4-Fp1	0.1447	0.1390	0.0558	0.8145
P4-F7	0.1331	0.1266	0.1015	0.7518

Theta	Mean WS	Mean TD	Signed F	p
P4-T3	0.1228	0.1148	0.1234	0.7273
P4-T5	0.0791	0.0695	0.3072	0.5827
P4-O1	0.0974	0.0598	5.3933	0.0257
P4-F4	0.1398	0.1550	-0.4301	0.5159
P4-C4	0.1823	0.1044	10.5027	0.0025
P4-P4				
P4-F3	0.1318	0.1539	-0.9200	0.3435
P4-C3	0.1405	0.0827	7.6549	0.0087
P4-P3	0.0701	0.0481	3.6799	0.0626
P4-Fpz1	0.1644	0.1562	0.0891	0.7670
P4-Fz	0.1388	0.1816	-2.9956	0.0916
P4-Cz	0.1676	0.1257	2.3464	0.1339
P4-Pz	0.1337	0.0732	6.7704	0.0131
P4-Oz	0.1331	0.0874	5.2218	0.0280
F3-Fp2	0.2092	0.1241	7.6372	0.0088
F3-F8	0.1253	0.0788	5.0543	0.0304
F3-T4	0.1105	0.1073	0.0270	0.8704
F3-T6	0.1161	0.1156	0.0007	0.9794
F3-O2	0.1146	0.1223	-0.1561	0.6950
F3-Fp1	0.2664	0.1784	5.5581	0.0236
F3-F7	0.1999	0.1175	8.1429	0.0070
F3-T3	0.1569	0.1134	2.3049	0.1372
F3-T5	0.1398	0.0876	6.3461	0.0161
F3-O1	0.1201	0.0949	2.3732	0.1317
F3-F4	0.0930	0.0549	5.5039	0.0243
F3-C4	0.1681	0.1912	-0.7560	0.3900
F3-P4	0.1318	0.1539	-0.9200	0.3435
F3-F3				
F3-C3	0.2167	0.2252	-0.0750	0.7857
F3-P3	0.1361	0.1304	0.0560	0.8143
F3-Fpz1	0.2812	0.1966	4.7506	0.0356
F3-Fz	0.1446	0.0989	2.3222	0.1358
F3-Cz	0.2211	0.1841	1.2713	0.2666
F3-Pz	0.1451	0.1560	-0.1666	0.6855
F3-Oz	0.1218	0.1116	0.2515	0.6189
C3-Fp2	0.1983	0.1752	0.5775	0.4520
C3-F8	0.1430	0.1469	-0.0231	0.8800
C3-T4	0.1243	0.0931	1.8091	0.1866
C3-T6	0.1341	0.0687	12.3890	0.0011
C3-O2	0.1165	0.0888	2.3563	0.1331
C3-Fp1	0.2176	0.1937	0.5229	0.4740
C3-F7	0.2048	0.1804	0.5646	0.4571
C3-T3	0.2199	0.1698	1.5332	0.2232
C3-T5	0.2040	0.0942	19.7065	0.0001
C3-O1	0.1489	0.0751	17.0678	0.0002
C3-F4	0.1680	0.1842	-0.3564	0.5540
C3-C4	0.0671	0.0593	0.4621	0.5008
C3-P4	0.1405	0.0827	7.6549	0.0087
C3-F3	0.2167	0.2252	-0.0750	0.7857
C3-C3				
C3-P3	0.1714	0.0897	11.0399	0.0020
C3-Fpz1	0.2283	0.2039	0.4931	0.4868
C3-Fz	0.2290	0.2528	-0.5226	0.4741

Theta	Mean WS	Mean TD	Signed F	p
C3-Cz	0.0921	0.1355	-3.6127	0.0649
C3-Pz	0.1350	0.0914	5.1521	0.0290
C3-Oz	0.1206	0.0927	1.9181	0.1741
P3-Fp2	0.1333	0.1301	0.0175	0.8955
P3-F8	0.1187	0.1247	-0.0868	0.7699
P3-T4	0.1077	0.0990	0.1869	0.6679
P3-T6	0.1044	0.0544	12.5383	0.0011
P3-O2	0.1128	0.0707	5.1705	0.0287
P3-Fp1	0.1364	0.1326	0.0206	0.8866
P3-F7	0.1409	0.1291	0.2451	0.6234
P3-T3	0.1700	0.1608	0.0816	0.7767
P3-T5	0.1614	0.1189	2.4566	0.1253
P3-O1	0.1561	0.0776	15.8793	0.0003
P3-F4	0.1250	0.1334	-0.1434	0.7070
P3-C4	0.1153	0.0769	4.1127	0.0496
P3-P4	0.0701	0.0481	3.6799	0.0626
P3-F3	0.1361	0.1304	0.0560	0.8143
P3-C3	0.1714	0.0897	11.0399	0.0020
P3-P3				
P3-Fpz1	0.1469	0.1419	0.0325	0.8580
P3-Fz	0.1365	0.1549	-0.5267	0.4724
P3-Cz	0.1485	0.1128	1.8666	0.1799
P3-Pz	0.1023	0.0819	1.0267	0.3173
P3-Oz	0.1408	0.0948	4.6985	0.0365
Fpz1-Fp2	0.1138	0.1077	0.1035	0.7494
Fpz1-F8	0.1329	0.1351	-0.0077	0.9304
Fpz1-T4	0.1448	0.1477	-0.0138	0.9070
Fpz1-T6	0.1416	0.1302	0.2539	0.6173
Fpz1-O2	0.1230	0.1105	0.4482	0.5072
Fpz1-Fp1	0.1059	0.0930	0.3275	0.5705
Fpz1-F7	0.1261	0.1175	0.1539	0.6970
Fpz1-T3	0.1196	0.1228	-0.0184	0.8927
Fpz1-T5	0.1138	0.1027	0.2478	0.6215
Fpz1-O1	0.1174	0.0988	1.0039	0.3227
Fpz1-F4	0.2862	0.2023	4.9264	0.0325
Fpz1-C4	0.2409	0.2089	0.7729	0.3848
Fpz1-P4	0.1644	0.1562	0.0891	0.7670
Fpz1-F3	0.2812	0.1966	4.7506	0.0356
Fpz1-C3	0.2283	0.2039	0.4931	0.4868
Fpz1-P3	0.1469	0.1419	0.0325	0.8580
Fpz1-Fpz1				
Fpz1-Fz	0.3098	0.1858	10.3552	0.0026
Fpz1-Cz	0.2435	0.1842	2.8025	0.1023
Fpz1-Pz	0.1670	0.1469	0.4683	0.4979
Fpz1-Oz	0.1306	0.1015	2.2109	0.1453
Fz-Fp2	0.2418	0.1326	11.8742	0.0014
Fz-F8	0.1380	0.0967	3.3812	0.0738
Fz-T4	0.1276	0.1152	0.3215	0.5741
Fz-T6	0.1141	0.1191	-0.0637	0.8021
Fz-O2	0.1181	0.1307	-0.3782	0.5422
Fz-Fp1	0.2329	0.1310	12.1976	0.0012
Fz-F7	0.1378	0.0890	5.9295	0.0197
Fz-T3	0.1266	0.1018	1.0668	0.3082

Theta	Mean WS	Mean TD	Signed F	p
Fz-T5	0.1202	0.0889	2.4010	0.1295
Fz-O1	0.1214	0.1090	0.4455	0.5085
Fz-F4	0.1387	0.1122	0.9026	0.3481
Fz-C4	0.2355	0.2599	-0.5398	0.4670
Fz-P4	0.1388	0.1816	-2.9956	0.0916
Fz-F3	0.1446	0.0989	2.3222	0.1358
Fz-C3	0.2290	0.2528	-0.5226	0.4741
Fz-P3	0.1365	0.1549	-0.5267	0.4724
Fz-Fpz1	0.3098	0.1858	10.3552	0.0026
Fz-Fz				
Fz-Cz	0.3029	0.2509	1.6849	0.2021
Fz-Pz	0.1617	0.1868	-0.7093	0.4050
Fz-Oz	0.1325	0.1234	0.1563	0.6948
Cz-Fp2	0.2222	0.1724	2.4528	0.1256
Cz-F8	0.1669	0.1487	0.3781	0.5423
Cz-T4	0.1506	0.1111	2.0460	0.1608
Cz-T6	0.1510	0.0929	7.0347	0.0116
Cz-O2	0.1177	0.0849	3.0356	0.0895
Cz-Fp1	0.2167	0.1671	2.4364	0.1268
Cz-F7	0.1823	0.1315	2.9893	0.0919
Cz-T3	0.1740	0.1078	4.2266	0.0467
Cz-T5	0.1667	0.0865	10.0896	0.0030
Cz-O1	0.1278	0.0742	9.1245	0.0045
Cz-F4	0.2307	0.1838	2.1473	0.1510
Cz-C4	0.1156	0.1725	-5.7827	0.0212
Cz-P4	0.1676	0.1257	2.3464	0.1339
Cz-F3	0.2211	0.1841	1.2713	0.2666
Cz-C3	0.0921	0.1355	-3.6127	0.0649
Cz-P3	0.1485	0.1128	1.8666	0.1799
Cz-Fpz1	0.2435	0.1842	2.8025	0.1023
Cz-Fz	0.3029	0.2509	1.6849	0.2021
Cz-Cz				
Cz-Pz	0.1400	0.1249	0.2932	0.5913
Cz-Oz	0.1177	0.0910	2.1139	0.1542
Pz-Fp2	0.1524	0.1389	0.2515	0.6189
Pz-F8	0.1334	0.1303	0.0204	0.8873
Pz-T4	0.1280	0.1082	0.6432	0.4276
Pz-T6	0.1281	0.0771	5.8109	0.0209
Pz-O2	0.1316	0.0775	7.2956	0.0103
Pz-Fp1	0.1525	0.1363	0.3689	0.5472
Pz-F7	0.1456	0.1242	0.8969	0.3496
Pz-T3	0.1516	0.1167	1.4823	0.2309
Pz-T5	0.1357	0.0966	2.3197	0.1360
Pz-O1	0.1322	0.0718	10.1689	0.0029
Pz-F4	0.1465	0.1577	-0.1874	0.6675
Pz-C4	0.1302	0.0875	3.5921	0.0657
Pz-P4	0.1337	0.0732	6.7704	0.0131
Pz-F3	0.1451	0.1560	-0.1666	0.6855
Pz-C3	0.1350	0.0914	5.1521	0.0290
Pz-P3	0.1023	0.0819	1.0267	0.3173
Pz-Fpz1	0.1670	0.1469	0.4683	0.4979
Pz-Fz	0.1617	0.1868	-0.7093	0.4050
Pz-Cz	0.1400	0.1249	0.2932	0.5913

Theta	Mean WS	Mean TD	Signed F	p
Pz-Pz				
Pz-Oz	0.1488	0.0926	6.9347	0.0122
Oz-Fp2	0.1219	0.1019	1.0729	0.3068
Oz-F8	0.1105	0.0993	0.3865	0.5379
Oz-T4	0.1256	0.1091	0.5424	0.4660
Oz-T6	0.1302	0.1086	0.8759	0.3552
Oz-O2	0.1415	0.1117	1.1531	0.2897
Oz-Fp1	0.1209	0.0963	1.8433	0.1826
Oz-F7	0.1140	0.0934	1.6477	0.2070
Oz-T3	0.1248	0.1024	1.0354	0.3153
Oz-T5	0.1209	0.1016	0.7623	0.3881
Oz-O1	0.1149	0.0983	0.7996	0.3768
Oz-F4	0.1239	0.1168	0.1085	0.7437
Oz-C4	0.1287	0.0891	3.4354	0.0716
Oz-P4	0.1331	0.0874	5.2218	0.0280
Oz-F3	0.1218	0.1116	0.2515	0.6189
Oz-C3	0.1206	0.0927	1.9181	0.1741
Oz-P3	0.1408	0.0948	4.6985	0.0365
Oz-Fpz1	0.1306	0.1015	2.2109	0.1453
Oz-Fz	0.1325	0.1234	0.1563	0.6948
Oz-Cz	0.1177	0.0910	2.1139	0.1542
Oz-Pz	0.1488	0.0926	6.9347	0.0122
Oz-Oz				

Supplementary Table S19. Group means, signed F-values and probabilities of region-specific REM sleep EEG alpha WPLI differences of Williams syndrome and typically developing subjects in all the possible electrode pairings. Color codes: Red = WS > TD (B-H corrected), Yellow = WS > TD (uncorrected), White = WS ≈ typically developing, Light blue = WS < TD (uncorrected), Blue = WS < TD (B-H corrected).

Alpha	Mean WS	Mean TD	Signed F	p
Fp2-Fp2				
Fp2-F8	0.1576	0.1669	-0.0983	0.7556
Fp2-T4	0.1375	0.1259	0.2525	0.6182
Fp2-T6	0.0956	0.0834	0.4634	0.5002
Fp2-O2	0.0722	0.0794	-0.1994	0.6577
Fp2-Fp1	0.0569	0.0371	5.2327	0.0278
Fp2-F7	0.0832	0.0838	-0.0012	0.9730
Fp2-T3	0.1066	0.1003	0.1121	0.7396
Fp2-T5	0.0815	0.0773	0.0568	0.8129
Fp2-O1	0.0787	0.0795	-0.0019	0.9655
Fp2-F4	0.2142	0.1709	1.7362	0.1955
Fp2-C4	0.1771	0.1823	-0.0383	0.8458
Fp2-P4	0.0917	0.1180	-2.5754	0.1168
Fp2-F3	0.1489	0.1028	3.5991	0.0654
Fp2-C3	0.1533	0.1616	-0.1431	0.7074
Fp2-P3	0.0796	0.1149	-5.1258	0.0294
Fp2-Fpz1	0.1126	0.0944	1.2305	0.2743
Fp2-Fz	0.1482	0.0984	3.5601	0.0668
Fp2-Cz	0.1548	0.1337	0.8293	0.3682
Fp2-Pz	0.0832	0.1284	-8.3550	0.0063
Fp2-Oz	0.0813	0.1127	-2.5260	0.1203
F8-Fp2	0.1576	0.1669	-0.0983	0.7556
F8-F8				
F8-T4	0.1455	0.1149	1.4712	0.2326
F8-T6	0.1232	0.0857	2.7061	0.1082
F8-O2	0.0913	0.0785	0.4964	0.4854
F8-Fp1	0.1284	0.1086	0.5959	0.4449
F8-F7	0.0942	0.0521	4.5558	0.0393
F8-T3	0.1018	0.0675	2.9409	0.0945
F8-T5	0.1070	0.0756	2.6596	0.1112
F8-O1	0.0923	0.0828	0.2744	0.6034
F8-F4	0.0863	0.0674	1.2385	0.2727
F8-C4	0.1260	0.1331	-0.1275	0.7230
F8-P4	0.0953	0.1013	-0.1083	0.7439
F8-F3	0.0791	0.0537	3.1241	0.0852
F8-C3	0.0917	0.1085	-1.2677	0.2673
F8-P3	0.0847	0.1030	-1.2360	0.2732
F8-Fpz1	0.1699	0.1607	0.0933	0.7617
F8-Fz	0.0747	0.0685	0.1633	0.6884
F8-Cz	0.0887	0.0917	-0.0416	0.8394
F8-Pz	0.0822	0.1078	-2.8039	0.1022
F8-Oz	0.0879	0.1076	-1.1723	0.2858
T4-Fp2	0.1375	0.1259	0.2525	0.6182
T4-F8	0.1455	0.1149	1.4712	0.2326
T4-T4				
T4-T6	0.1348	0.0903	3.9630	0.0537
T4-O2	0.1075	0.0898	0.8534	0.3614
T4-Fp1	0.1287	0.1175	0.2886	0.5942
T4-F7	0.1100	0.0950	0.6578	0.4224
T4-T3	0.0970	0.0537	6.8644	0.0126

Alpha	Mean WS	Mean TD	Signed F	p
T4-T5	0.0924	0.0639	2.7751	0.1040
T4-O1	0.0908	0.0776	0.6477	0.4259
T4-F4	0.1190	0.1020	0.5328	0.4699
T4-C4	0.1158	0.0802	3.3311	0.0758
T4-P4	0.0924	0.0750	0.7355	0.3965
T4-F3	0.0933	0.0962	-0.0283	0.8672
T4-C3	0.0818	0.0659	1.8300	0.1841
T4-P3	0.0872	0.0708	1.1487	0.2906
T4-Fpz1	0.1503	0.1292	0.7971	0.3776
T4-Fz	0.1108	0.1030	0.1558	0.6953
T4-Cz	0.1012	0.0740	2.5987	0.1152
T4-Pz	0.0884	0.0577	3.7948	0.0588
T4-Oz	0.0953	0.0724	1.9623	0.1694
T6-Fp2	0.0956	0.0834	0.4634	0.5002
T6-F8	0.1232	0.0857	2.7061	0.1082
T6-T4	0.1348	0.0903	3.9630	0.0537
T6-T6				
T6-O2	0.1316	0.1549	-0.7310	0.3979
T6-Fp1	0.1002	0.0822	0.8995	0.3489
T6-F7	0.1034	0.0830	1.2384	0.2728
T6-T3	0.1112	0.0607	10.3356	0.0027
T6-T5	0.0797	0.0567	2.2318	0.1435
T6-O1	0.0801	0.0629	1.2878	0.2636
T6-F4	0.1062	0.0833	1.8892	0.1773
T6-C4	0.1269	0.1003	1.8618	0.1804
T6-P4	0.1053	0.1186	-0.2481	0.6213
T6-F3	0.0869	0.0814	0.0975	0.7565
T6-C3	0.1046	0.0690	4.3119	0.0447
T6-P3	0.0799	0.0591	1.6994	0.2002
T6-Fpz1	0.1052	0.0845	1.1738	0.2855
T6-Fz	0.0892	0.0847	0.0750	0.7857
T6-Cz	0.1120	0.0770	3.9861	0.0531
T6-Pz	0.0972	0.0860	0.2878	0.5947
T6-Oz	0.1091	0.1406	-1.6341	0.2089
O2-Fp2	0.0722	0.0794	-0.1994	0.6577
O2-F8	0.0913	0.0785	0.4964	0.4854
O2-T4	0.1075	0.0898	0.8534	0.3614
O2-T6	0.1316	0.1549	-0.7310	0.3979
O2-O2				
O2-Fp1	0.0797	0.0779	0.0107	0.9180
O2-F7	0.0910	0.0764	0.7069	0.4057
O2-T3	0.1016	0.0666	6.2185	0.0171
O2-T5	0.0967	0.0669	2.4285	0.1274
O2-O1	0.0780	0.0701	0.2170	0.6440
O2-F4	0.0803	0.0766	0.0614	0.8056
O2-C4	0.1011	0.0869	0.6459	0.4266
O2-P4	0.1088	0.0998	0.2129	0.6471
O2-F3	0.0877	0.0774	0.4515	0.5057
O2-C3	0.1099	0.0652	7.4727	0.0095
O2-P3	0.1115	0.0650	8.9715	0.0048
O2-Fpz1	0.0803	0.0805	-0.0002	0.9880
O2-Fz	0.0812	0.0780	0.0513	0.8220
O2-Cz	0.1020	0.0696	3.7077	0.0617
O2-Pz	0.1230	0.0996	1.0297	0.3166
O2-Oz	0.1211	0.2204	-9.4568	0.0039
Fp1-Fp2	0.0569	0.0371	5.2327	0.0278

Alpha	Mean WS	Mean TD	Signed F	p
Fp1-F8	0.1284	0.1086	0.5959	0.4449
Fp1-T4	0.1287	0.1175	0.2886	0.5942
Fp1-T6	0.1002	0.0822	0.8995	0.3489
Fp1-O2	0.0797	0.0779	0.0107	0.9180
Fp1-Fp1				
Fp1-F7	0.1201	0.1428	-0.8787	0.3545
Fp1-T3	0.1301	0.1145	0.5940	0.4457
Fp1-T5	0.0893	0.0783	0.2965	0.5893
Fp1-O1	0.0830	0.0766	0.1157	0.7356
Fp1-F4	0.1675	0.1138	3.8295	0.0577
Fp1-C4	0.1685	0.1546	0.3395	0.5636
Fp1-P4	0.1014	0.1109	-0.2877	0.5948
Fp1-F3	0.2132	0.1631	2.7787	0.1037
Fp1-C3	0.1809	0.1792	0.0053	0.9421
Fp1-P3	0.0917	0.1104	-1.2023	0.2798
Fp1-Fpz1	0.0897	0.0825	0.1701	0.6823
Fp1-Fz	0.1589	0.1062	3.7375	0.0607
Fp1-Cz	0.1614	0.1291	1.9833	0.1672
Fp1-Pz	0.0946	0.1213	-2.4877	0.1230
Fp1-Oz	0.0881	0.1115	-1.2421	0.2721
F7-Fp2	0.0832	0.0838	-0.0012	0.9730
F7-F8	0.0942	0.0521	4.5558	0.0393
F7-T4	0.1100	0.0950	0.6578	0.4224
F7-T6	0.1034	0.0830	1.2384	0.2728
F7-O2	0.0910	0.0764	0.7069	0.4057
F7-Fp1	0.1201	0.1428	-0.8787	0.3545
F7-F7				
F7-T3	0.1198	0.1135	0.0927	0.7624
F7-T5	0.1082	0.0811	1.9264	0.1732
F7-O1	0.0923	0.0729	0.9846	0.3273
F7-F4	0.0881	0.0591	4.0865	0.0503
F7-C4	0.1252	0.1046	1.4611	0.2342
F7-P4	0.1023	0.0918	0.3960	0.5329
F7-F3	0.1225	0.0730	5.4670	0.0247
F7-C3	0.1459	0.1352	0.2936	0.5911
F7-P3	0.0975	0.0934	0.0542	0.8172
F7-Fpz1	0.1205	0.1328	-0.2672	0.6082
F7-Fz	0.0839	0.0611	2.3277	0.1354
F7-Cz	0.1155	0.0908	2.0917	0.1563
F7-Pz	0.0975	0.0964	0.0041	0.9493
F7-Oz	0.0967	0.1079	-0.3088	0.5817
T3-Fp2	0.1066	0.1003	0.1121	0.7396
T3-F8	0.1018	0.0675	2.9409	0.0945
T3-T4	0.0970	0.0537	6.8644	0.0126
T3-T6	0.1112	0.0607	10.3356	0.0027
T3-O2	0.1016	0.0666	6.2185	0.0171
T3-Fp1	0.1301	0.1145	0.5940	0.4457
T3-F7	0.1198	0.1135	0.0927	0.7624
T3-T3				
T3-T5	0.1034	0.0819	1.4425	0.2372
T3-O1	0.1017	0.0747	2.0405	0.1613
T3-F4	0.0870	0.0846	0.0213	0.8846
T3-C4	0.1056	0.0565	8.7105	0.0054
T3-P4	0.1026	0.0556	9.7421	0.0034
T3-F3	0.1007	0.0975	0.0171	0.8965
T3-C3	0.1228	0.0865	4.1116	0.0496

Alpha	Mean WS	Mean TD	Signed F	p
T3-P3	0.1124	0.0771	5.3932	0.0257
T3-Fpz1	0.1297	0.1137	0.5734	0.4536
T3-Fz	0.0900	0.0947	-0.0676	0.7963
T3-Cz	0.0963	0.0799	1.0991	0.3011
T3-Pz	0.1015	0.0589	8.5784	0.0057
T3-Oz	0.1041	0.0764	2.7797	0.1037
T5-Fp2	0.0815	0.0773	0.0568	0.8129
T5-F8	0.1070	0.0756	2.6596	0.1112
T5-T4	0.0924	0.0639	2.7751	0.1040
T5-T6	0.0797	0.0567	2.2318	0.1435
T5-O2	0.0967	0.0669	2.4285	0.1274
T5-Fp1	0.0893	0.0783	0.2965	0.5893
T5-F7	0.1082	0.0811	1.9264	0.1732
T5-T3	0.1034	0.0819	1.4425	0.2372
T5-T5				
T5-O1	0.1278	0.1282	-0.0003	0.9865
T5-F4	0.1027	0.0819	1.6508	0.2066
T5-C4	0.1204	0.0818	3.5804	0.0661
T5-P4	0.0856	0.0756	0.2737	0.6039
T5-F3	0.1205	0.0899	1.8482	0.1820
T5-C3	0.1448	0.1008	3.7357	0.0607
T5-P3	0.1209	0.1077	0.2963	0.5894
T5-Fpz1	0.0858	0.0784	0.1352	0.7152
T5-Fz	0.0990	0.0846	0.7866	0.3807
T5-Cz	0.1223	0.0871	3.6952	0.0621
T5-Pz	0.1112	0.1066	0.0442	0.8346
T5-Oz	0.1067	0.1367	-1.4295	0.2393
O1-Fp2	0.0787	0.0795	-0.0019	0.9655
O1-F8	0.0923	0.0828	0.2744	0.6034
O1-T4	0.0908	0.0776	0.6477	0.4259
O1-T6	0.0801	0.0629	1.2878	0.2636
O1-O2	0.0780	0.0701	0.2170	0.6440
O1-Fp1	0.0830	0.0766	0.1157	0.7356
O1-F7	0.0923	0.0729	0.9846	0.3273
O1-T3	0.1017	0.0747	2.0405	0.1613
O1-T5	0.1278	0.1282	-0.0003	0.9865
O1-O1				
O1-F4	0.0935	0.0779	0.7956	0.3780
O1-C4	0.1106	0.0807	1.9428	0.1715
O1-P4	0.0925	0.0840	0.1694	0.6830
O1-F3	0.0982	0.0783	1.2092	0.2784
O1-C3	0.1248	0.0816	4.2848	0.0453
O1-P3	0.1299	0.0977	1.5898	0.2150
O1-Fpz1	0.0830	0.0786	0.0523	0.8203
O1-Fz	0.0906	0.0765	0.8070	0.3747
O1-Cz	0.1103	0.0804	2.7314	0.1066
O1-Pz	0.1196	0.1205	-0.0011	0.9741
O1-Oz	0.1136	0.2334	-14.0366	0.0006
F4-Fp2	0.2142	0.1709	1.7362	0.1955
F4-F8	0.0863	0.0674	1.2385	0.2727
F4-T4	0.1190	0.1020	0.5328	0.4699
F4-T6	0.1062	0.0833	1.8892	0.1773
F4-O2	0.0803	0.0766	0.0614	0.8056
F4-Fp1	0.1675	0.1138	3.8295	0.0577
F4-F7	0.0881	0.0591	4.0865	0.0503
F4-T3	0.0870	0.0846	0.0213	0.8846

Alpha	Mean WS	Mean TD	Signed F	p
F4-T5	0.1027	0.0819	1.6508	0.2066
F4-O1	0.0935	0.0779	0.7956	0.3780
F4-F4				
F4-C4	0.1750	0.2079	-1.8482	0.1820
F4-P4	0.1086	0.1161	-0.1357	0.7146
F4-F3	0.0794	0.0468	4.6670	0.0371
F4-C3	0.1274	0.1603	-2.9116	0.0961
F4-P3	0.0966	0.1168	-1.1936	0.2815
F4-Fpz1	0.2350	0.1780	2.7787	0.1037
F4-Fz	0.1355	0.0895	4.9004	0.0329
F4-Cz	0.1421	0.1285	0.4184	0.5216
F4-Pz	0.0937	0.1288	-4.2323	0.0466
F4-Oz	0.1000	0.0975	0.0191	0.8909
C4-Fp2	0.1771	0.1823	-0.0383	0.8458
C4-F8	0.1260	0.1331	-0.1275	0.7230
C4-T4	0.1158	0.0802	3.3311	0.0758
C4-T6	0.1269	0.1003	1.8618	0.1804
C4-O2	0.1011	0.0869	0.6459	0.4266
C4-Fp1	0.1685	0.1546	0.3395	0.5636
C4-F7	0.1252	0.1046	1.4611	0.2342
C4-T3	0.1056	0.0565	8.7105	0.0054
C4-T5	0.1204	0.0818	3.5804	0.0661
C4-O1	0.1106	0.0807	1.9428	0.1715
C4-F4	0.1750	0.2079	-1.8482	0.1820
C4-C4				
C4-P4	0.1292	0.1023	1.0212	0.3186
C4-F3	0.1366	0.1544	-0.7495	0.3921
C4-C3	0.0654	0.0418	4.8221	0.0343
C4-P3	0.1168	0.0892	1.4197	0.2408
C4-Fpz1	0.1926	0.1765	0.3430	0.5616
C4-Fz	0.1895	0.2015	-0.2038	0.6543
C4-Cz	0.1031	0.1317	-1.6387	0.2083
C4-Pz	0.1214	0.0858	2.0290	0.1625
C4-Oz	0.1148	0.0763	4.8768	0.0333
P4-Fp2	0.0917	0.1180	-2.5754	0.1168
P4-F8	0.0953	0.1013	-0.1083	0.7439
P4-T4	0.0924	0.0750	0.7355	0.3965
P4-T6	0.1053	0.1186	-0.2481	0.6213
P4-O2	0.1088	0.0998	0.2129	0.6471
P4-Fp1	0.1014	0.1109	-0.2877	0.5948
P4-F7	0.1023	0.0918	0.3960	0.5329
P4-T3	0.1026	0.0556	9.7421	0.0034
P4-T5	0.0856	0.0756	0.2737	0.6039
P4-O1	0.0925	0.0840	0.1694	0.6830
P4-F4	0.1086	0.1161	-0.1357	0.7146
P4-C4	0.1292	0.1023	1.0212	0.3186
P4-P4				
P4-F3	0.1017	0.1128	-0.3555	0.5545
P4-C3	0.1225	0.0642	7.9257	0.0077
P4-P3	0.0736	0.0597	0.9462	0.3368
P4-Fpz1	0.1081	0.1168	-0.2320	0.6328
P4-Fz	0.0989	0.1232	-1.5922	0.2147
P4-Cz	0.1343	0.1035	1.6908	0.2013
P4-Pz	0.0879	0.1031	-0.6243	0.4344
P4-Oz	0.0995	0.1031	-0.0418	0.8390
F3-Fp2	0.1489	0.1028	3.5991	0.0654

Alpha	Mean WS	Mean TD	Signed F	p
F3-F8	0.0791	0.0537	3.1241	0.0852
F3-T4	0.0933	0.0962	-0.0283	0.8672
F3-T6	0.0869	0.0814	0.0975	0.7565
F3-O2	0.0877	0.0774	0.4515	0.5057
F3-Fp1	0.2132	0.1631	2.7787	0.1037
F3-F7	0.1225	0.0730	5.4670	0.0247
F3-T3	0.1007	0.0975	0.0171	0.8965
F3-T5	0.1205	0.0899	1.8482	0.1820
F3-O1	0.0982	0.0783	1.2092	0.2784
F3-F4	0.0794	0.0468	4.6670	0.0371
F3-C4	0.1366	0.1544	-0.7495	0.3921
F3-P4	0.1017	0.1128	-0.3555	0.5545
F3-F3				
F3-C3	0.1847	0.2061	-0.7039	0.4067
F3-P3	0.1083	0.1156	-0.1358	0.7145
F3-Fpz1	0.2242	0.1703	2.8213	0.1012
F3-Fz	0.1310	0.0720	7.6368	0.0088
F3-Cz	0.1468	0.1317	0.4876	0.4893
F3-Pz	0.0965	0.1245	-2.7516	0.1054
F3-Oz	0.1021	0.1013	0.0020	0.9647
C3-Fp2	0.1533	0.1616	-0.1431	0.7074
C3-F8	0.0917	0.1085	-1.2677	0.2673
C3-T4	0.0818	0.0659	1.8300	0.1841
C3-T6	0.1046	0.0690	4.3119	0.0447
C3-O2	0.1099	0.0652	7.4727	0.0095
C3-Fp1	0.1809	0.1792	0.0053	0.9421
C3-F7	0.1459	0.1352	0.2936	0.5911
C3-T3	0.1228	0.0865	4.1116	0.0496
C3-T5	0.1448	0.1008	3.7357	0.0607
C3-O1	0.1248	0.0816	4.2848	0.0453
C3-F4	0.1274	0.1603	-2.9116	0.0961
C3-C4	0.0654	0.0418	4.8221	0.0343
C3-P4	0.1225	0.0642	7.9257	0.0077
C3-F3	0.1847	0.2061	-0.7039	0.4067
C3-C3				
C3-P3	0.1481	0.1127	1.9884	0.1666
C3-Fpz1	0.1881	0.1784	0.1589	0.6924
C3-Fz	0.1923	0.2058	-0.2916	0.5924
C3-Cz	0.0863	0.1397	-5.9275	0.0197
C3-Pz	0.1274	0.0762	4.6949	0.0366
C3-Oz	0.1258	0.0782	8.5786	0.0057
P3-Fp2	0.0796	0.1149	-5.1258	0.0294
P3-F8	0.0847	0.1030	-1.2360	0.2732
P3-T4	0.0872	0.0708	1.1487	0.2906
P3-T6	0.0799	0.0591	1.6994	0.2002
P3-O2	0.1115	0.0650	8.9715	0.0048
P3-Fp1	0.0917	0.1104	-1.2023	0.2798
P3-F7	0.0975	0.0934	0.0542	0.8172
P3-T3	0.1124	0.0771	5.3932	0.0257
P3-T5	0.1209	0.1077	0.2963	0.5894
P3-O1	0.1299	0.0977	1.5898	0.2150
P3-F4	0.0966	0.1168	-1.1936	0.2815
P3-C4	0.1168	0.0892	1.4197	0.2408
P3-P4	0.0736	0.0597	0.9462	0.3368
P3-F3	0.1083	0.1156	-0.1358	0.7145
P3-C3	0.1481	0.1127	1.9884	0.1666

Alpha	Mean WS	Mean TD	Signed F	p
P3-P3				
P3-Fpz1	0.0957	0.1139	-1.1957	0.2811
P3-Fz	0.0915	0.1222	-2.8748	0.0982
P3-Cz	0.1289	0.1156	0.3395	0.5635
P3-Pz	0.0880	0.1393	-4.2289	0.0467
P3-Oz	0.1159	0.0982	0.9680	0.3314
Fpz1-Fp2	0.1126	0.0944	1.2305	0.2743
Fpz1-F8	0.1699	0.1607	0.0933	0.7617
Fpz1-T4	0.1503	0.1292	0.7971	0.3776
Fpz1-T6	0.1052	0.0845	1.1738	0.2855
Fpz1-O2	0.0803	0.0805	-0.0002	0.9880
Fpz1-Fp1	0.0897	0.0825	0.1701	0.6823
Fpz1-F7	0.1205	0.1328	-0.2672	0.6082
Fpz1-T3	0.1297	0.1137	0.5734	0.4536
Fpz1-T5	0.0858	0.0784	0.1352	0.7152
Fpz1-O1	0.0830	0.0786	0.0523	0.8203
Fpz1-F4	0.2350	0.1780	2.7787	0.1037
Fpz1-C4	0.1926	0.1765	0.3430	0.5616
Fpz1-P4	0.1081	0.1168	-0.2320	0.6328
Fpz1-F3	0.2242	0.1703	2.8213	0.1012
Fpz1-C3	0.1881	0.1784	0.1589	0.6924
Fpz1-P3	0.0957	0.1139	-1.1957	0.2811
Fpz1-Fpz1				
Fpz1-Fz	0.2193	0.1486	4.4071	0.0425
Fpz1-Cz	0.1807	0.1436	2.1049	0.1550
Fpz1-Pz	0.1003	0.1273	-2.4094	0.1289
Fpz1-Oz	0.0889	0.1149	-1.5128	0.2263
Fz-Fp2	0.1482	0.0984	3.5601	0.0668
Fz-F8	0.0747	0.0685	0.1633	0.6884
Fz-T4	0.1108	0.1030	0.1558	0.6953
Fz-T6	0.0892	0.0847	0.0750	0.7857
Fz-O2	0.0812	0.0780	0.0513	0.8220
Fz-Fp1	0.1589	0.1062	3.7375	0.0607
Fz-F7	0.0839	0.0611	2.3277	0.1354
Fz-T3	0.0900	0.0947	-0.0676	0.7963
Fz-T5	0.0990	0.0846	0.7866	0.3807
Fz-O1	0.0906	0.0765	0.8070	0.3747
Fz-F4	0.1355	0.0895	4.9004	0.0329
Fz-C4	0.1895	0.2015	-0.2038	0.6543
Fz-P4	0.0989	0.1232	-1.5922	0.2147
Fz-F3	0.1310	0.0720	7.6368	0.0088
Fz-C3	0.1923	0.2058	-0.2916	0.5924
Fz-P3	0.0915	0.1222	-2.8748	0.0982
Fz-Fpz1	0.2193	0.1486	4.4071	0.0425
Fz-Fz				
Fz-Cz	0.2206	0.1858	1.6594	0.2055
Fz-Pz	0.0925	0.1386	-6.7586	0.0132
Fz-Oz	0.0975	0.1010	-0.0341	0.8545
Cz-Fp2	0.1548	0.1337	0.8293	0.3682
Cz-F8	0.0887	0.0917	-0.0416	0.8394
Cz-T4	0.1012	0.0740	2.5987	0.1152
Cz-T6	0.1120	0.0770	3.9861	0.0531
Cz-O2	0.1020	0.0696	3.7077	0.0617
Cz-Fp1	0.1614	0.1291	1.9833	0.1672
Cz-F7	0.1155	0.0908	2.0917	0.1563
Cz-T3	0.0963	0.0799	1.0991	0.3011

Alpha	Mean WS	Mean TD	Signed F	p
Cz-T5	0.1223	0.0871	3.6952	0.0621
Cz-O1	0.1103	0.0804	2.7314	0.1066
Cz-F4	0.1421	0.1285	0.4184	0.5216
Cz-C4	0.1031	0.1317	-1.6387	0.2083
Cz-P4	0.1343	0.1035	1.6908	0.2013
Cz-F3	0.1468	0.1317	0.4876	0.4893
Cz-C3	0.0863	0.1397	-5.9275	0.0197
Cz-P3	0.1289	0.1156	0.3395	0.5635
Cz-Fpz1	0.1807	0.1436	2.1049	0.1550
Cz-Fz	0.2206	0.1858	1.6594	0.2055
Cz-Cz				
Cz-Pz	0.1293	0.1247	0.0369	0.8487
Cz-Oz	0.1137	0.0922	1.3035	0.2607
Pz-Fp2	0.0832	0.1284	-8.3550	0.0063
Pz-F8	0.0822	0.1078	-2.8039	0.1022
Pz-T4	0.0884	0.0577	3.7948	0.0588
Pz-T6	0.0972	0.0860	0.2878	0.5947
Pz-O2	0.1230	0.0996	1.0297	0.3166
Pz-Fp1	0.0946	0.1213	-2.4877	0.1230
Pz-F7	0.0975	0.0964	0.0041	0.9493
Pz-T3	0.1015	0.0589	8.5784	0.0057
Pz-T5	0.1112	0.1066	0.0442	0.8346
Pz-O1	0.1196	0.1205	-0.0011	0.9741
Pz-F4	0.0937	0.1288	-4.2323	0.0466
Pz-C4	0.1214	0.0858	2.0290	0.1625
Pz-P4	0.0879	0.1031	-0.6243	0.4344
Pz-F3	0.0965	0.1245	-2.7516	0.1054
Pz-C3	0.1274	0.0762	4.6949	0.0366
Pz-P3	0.0880	0.1393	-4.2289	0.0467
Pz-Fpz1	0.1003	0.1273	-2.4094	0.1289
Pz-Fz	0.0925	0.1386	-6.7586	0.0132
Pz-Cz	0.1293	0.1247	0.0369	0.8487
Pz-Pz				
Pz-Oz	0.1236	0.0980	1.4532	0.2355
Oz-Fp2	0.0813	0.1127	-2.5260	0.1203
Oz-F8	0.0879	0.1076	-1.1723	0.2858
Oz-T4	0.0953	0.0724	1.9623	0.1694
Oz-T6	0.1091	0.1406	-1.6341	0.2089
Oz-O2	0.1211	0.2204	-9.4568	0.0039
Oz-Fp1	0.0881	0.1115	-1.2421	0.2721
Oz-F7	0.0967	0.1079	-0.3088	0.5817
Oz-T3	0.1041	0.0764	2.7797	0.1037
Oz-T5	0.1067	0.1367	-1.4295	0.2393
Oz-O1	0.1136	0.2334	-14.0366	0.0006
Oz-F4	0.1000	0.0975	0.0191	0.8909
Oz-C4	0.1148	0.0763	4.8768	0.0333
Oz-P4	0.0995	0.1031	-0.0418	0.8390
Oz-F3	0.1021	0.1013	0.0020	0.9647
Oz-C3	0.1258	0.0782	8.5786	0.0057
Oz-P3	0.1159	0.0982	0.9680	0.3314
Oz-Fpz1	0.0889	0.1149	-1.5128	0.2263
Oz-Fz	0.0975	0.1010	-0.0341	0.8545
Oz-Cz	0.1137	0.0922	1.3035	0.2607
Oz-Pz	0.1236	0.0980	1.4532	0.2355
Oz-Oz				

Supplementary Table S20. Group means, signed F-values and probabilities of region-specific REM sleep EEG beta WPLI differences of Williams syndrome and typically developing subjects in all the possible electrode pairings. Color codes: Red = WS > TD (B-H corrected), Yellow = WS > TD (uncorrected), White = WS ≈ typically developing, Light blue = WS < TD (uncorrected), Blue = WS < TD (B-H corrected).

Beta	Mean WS	Mean TD	Signed F	p
Fp2-Fp2				
Fp2-F8	0.1534	0.1319	0.8370	0.3660
Fp2-T4	0.1264	0.0981	1.8453	0.1823
Fp2-T6	0.1617	0.0876	7.6026	0.0089
Fp2-O2	0.1417	0.0718	6.6033	0.0142
Fp2-Fp1	0.0813	0.0603	1.4815	0.2310
Fp2-F7	0.1011	0.0757	1.9260	0.1733
Fp2-T3	0.1141	0.0829	1.8167	0.1857
Fp2-T5	0.1149	0.0782	3.3885	0.0735
Fp2-O1	0.0971	0.0504	4.8714	0.0334
Fp2-F4	0.2762	0.1126	12.5714	0.0011
Fp2-C4	0.1917	0.0713	11.1947	0.0019
Fp2-P4	0.1322	0.0618	8.4441	0.0061
Fp2-F3	0.1470	0.0642	6.1692	0.0175
Fp2-C3	0.1400	0.0812	3.8263	0.0578
Fp2-P3	0.1027	0.0697	2.6963	0.1088
Fp2-Fpz1	0.1250	0.0997	0.7945	0.3783
Fp2-Fz	0.1909	0.0670	10.5229	0.0025
Fp2-Cz	0.1857	0.0771	8.5170	0.0059
Fp2-Pz	0.1287	0.0639	5.8916	0.0201
Fp2-Oz	0.1298	0.0597	6.3628	0.0160
F8-Fp2	0.1534	0.1319	0.8370	0.3660
F8-F8				
F8-T4	0.1392	0.0873	5.2298	0.0279
F8-T6	0.1733	0.0977	6.6289	0.0141
F8-O2	0.1546	0.0836	6.0041	0.0190
F8-Fp1	0.1202	0.1038	0.4948	0.4861
F8-F7	0.1230	0.0976	1.0236	0.3181
F8-T3	0.1214	0.0851	2.0397	0.1614
F8-T5	0.1145	0.0693	4.9817	0.0316
F8-O1	0.0978	0.0500	5.7574	0.0214
F8-F4	0.2163	0.0542	20.9570	0.0000
F8-C4	0.1939	0.0574	19.5237	0.0001
F8-P4	0.1330	0.0620	9.4831	0.0038
F8-F3	0.1122	0.0694	3.0489	0.0889
F8-C3	0.1128	0.0703	3.0621	0.0882
F8-P3	0.0966	0.0587	5.5859	0.0233
F8-Fpz1	0.1590	0.1381	0.5035	0.4823
F8-Fz	0.1288	0.0765	3.4530	0.0709
F8-Cz	0.1741	0.0630	14.4806	0.0005
F8-Pz	0.1265	0.0543	10.2326	0.0028
F8-Oz	0.1386	0.0705	6.0932	0.0182
T4-Fp2	0.1264	0.0981	1.8453	0.1823
T4-F8	0.1392	0.0873	5.2298	0.0279
T4-T4				
T4-T6	0.1628	0.0888	7.2403	0.0105
T4-O2	0.1544	0.0840	5.9252	0.0197
T4-Fp1	0.1283	0.0935	2.3047	0.1373
T4-F7	0.1535	0.0999	4.0021	0.0526
T4-T3	0.1411	0.0825	5.1103	0.0296

Beta	Mean WS	Mean TD	Signed F	p
T4-T5	0.1132	0.0508	11.2749	0.0018
T4-O1	0.0938	0.0497	4.3344	0.0441
T4-F4	0.1050	0.0619	3.7550	0.0601
T4-C4	0.1235	0.0523	6.7706	0.0131
T4-P4	0.1206	0.0581	6.3931	0.0157
T4-F3	0.1080	0.0770	2.2596	0.1411
T4-C3	0.1024	0.0618	4.1447	0.0488
T4-P3	0.0910	0.0430	9.5331	0.0038
T4-Fpz1	0.1457	0.1047	2.8874	0.0975
T4-Fz	0.1080	0.0763	2.5785	0.1166
T4-Cz	0.1036	0.0504	4.1327	0.0491
T4-Pz	0.1085	0.0407	8.3715	0.0063
T4-Oz	0.1359	0.0739	4.6306	0.0378
T6-Fp2	0.1617	0.0876	7.6026	0.0089
T6-F8	0.1733	0.0977	6.6289	0.0141
T6-T4	0.1628	0.0888	7.2403	0.0105
T6-T6				
T6-O2	0.1665	0.0797	11.0697	0.0020
T6-Fp1	0.1622	0.0861	7.2428	0.0105
T6-F7	0.1735	0.0857	8.8643	0.0050
T6-T3	0.1517	0.0723	9.3472	0.0041
T6-T5	0.0914	0.0425	13.9009	0.0006
T6-O1	0.0876	0.0520	3.3124	0.0766
T6-F4	0.1191	0.0807	4.7303	0.0359
T6-C4	0.1066	0.0625	9.3505	0.0041
T6-P4	0.0810	0.0481	4.9317	0.0324
T6-F3	0.1331	0.0858	4.9238	0.0325
T6-C3	0.1072	0.0640	8.3045	0.0065
T6-P3	0.0775	0.0360	16.3647	0.0002
T6-Fpz1	0.1728	0.0899	7.6565	0.0087
T6-Fz	0.1284	0.0850	4.5426	0.0396
T6-Cz	0.0945	0.0555	8.7924	0.0052
T6-Pz	0.0841	0.0395	11.2037	0.0018
T6-Oz	0.1350	0.0679	6.0384	0.0187
O2-Fp2	0.1417	0.0718	6.6033	0.0142
O2-F8	0.1546	0.0836	6.0041	0.0190
O2-T4	0.1544	0.0840	5.9252	0.0197
O2-T6	0.1665	0.0797	11.0697	0.0020
O2-O2				
O2-Fp1	0.1337	0.0639	7.2104	0.0107
O2-F7	0.1330	0.0559	9.9437	0.0031
O2-T3	0.1158	0.0503	9.4750	0.0039
O2-T5	0.0816	0.0453	7.2838	0.0103
O2-O1	0.0616	0.0492	0.8382	0.3657
O2-F4	0.1245	0.0683	7.6354	0.0088
O2-C4	0.1137	0.0607	9.6081	0.0036
O2-P4	0.1249	0.0572	14.5321	0.0005
O2-F3	0.1237	0.0644	8.6040	0.0057
O2-C3	0.1053	0.0549	10.2364	0.0028
O2-P3	0.0937	0.0381	13.0965	0.0009
O2-Fpz1	0.1403	0.0699	6.3748	0.0159
O2-Fz	0.1283	0.0646	9.0252	0.0047
O2-Cz	0.0938	0.0503	10.6454	0.0023
O2-Pz	0.0894	0.0421	12.7508	0.0010
O2-Oz	0.0753	0.0552	1.8979	0.1764
Fp1-Fp2	0.0813	0.0603	1.4815	0.2310

Beta	Mean WS	Mean TD	Signed F	p
Fp1-F8	0.1202	0.1038	0.4948	0.4861
Fp1-T4	0.1283	0.0935	2.3047	0.1373
Fp1-T6	0.1622	0.0861	7.2428	0.0105
Fp1-O2	0.1337	0.0639	7.2104	0.0107
Fp1-Fp1				
Fp1-F7	0.1191	0.0862	2.9775	0.0926
Fp1-T3	0.1164	0.0645	4.7599	0.0354
Fp1-T5	0.1179	0.0683	5.6222	0.0229
Fp1-O1	0.0976	0.0501	5.8728	0.0202
Fp1-F4	0.2032	0.0839	8.4207	0.0061
Fp1-C4	0.1733	0.0668	9.8521	0.0033
Fp1-P4	0.1327	0.0601	9.2039	0.0043
Fp1-F3	0.2551	0.1036	10.3923	0.0026
Fp1-C3	0.1830	0.0742	7.9904	0.0075
Fp1-P3	0.1092	0.0654	3.6564	0.0634
Fp1-Fpz1	0.0817	0.0599	1.3627	0.2503
Fp1-Fz	0.2217	0.0632	12.7791	0.0010
Fp1-Cz	0.1967	0.0713	9.6177	0.0036
Fp1-Pz	0.1362	0.0630	7.0738	0.0114
Fp1-Oz	0.1201	0.0515	6.8092	0.0129
F7-Fp2	0.1011	0.0757	1.9260	0.1733
F7-F8	0.1230	0.0976	1.0236	0.3181
F7-T4	0.1535	0.0999	4.0021	0.0526
F7-T6	0.1735	0.0857	8.8643	0.0050
F7-O2	0.1330	0.0559	9.9437	0.0031
F7-Fp1	0.1191	0.0862	2.9775	0.0926
F7-F7				
F7-T3	0.1006	0.0652	2.6602	0.1111
F7-T5	0.1095	0.0685	4.3373	0.0441
F7-O1	0.0885	0.0623	1.8011	0.1875
F7-F4	0.1787	0.0657	10.9625	0.0020
F7-C4	0.1742	0.0580	14.8318	0.0004
F7-P4	0.1349	0.0544	14.1178	0.0006
F7-F3	0.2364	0.0642	17.2969	0.0002
F7-C3	0.2021	0.0633	15.1486	0.0004
F7-P3	0.1079	0.0618	5.2920	0.0270
F7-Fpz1	0.1106	0.0846	1.4407	0.2374
F7-Fz	0.1804	0.0556	11.9220	0.0014
F7-Cz	0.1997	0.0586	14.8973	0.0004
F7-Pz	0.1398	0.0534	12.5752	0.0011
F7-Oz	0.1112	0.0725	1.4232	0.2403
T3-Fp2	0.1141	0.0829	1.8167	0.1857
T3-F8	0.1214	0.0851	2.0397	0.1614
T3-T4	0.1411	0.0825	5.1103	0.0296
T3-T6	0.1517	0.0723	9.3472	0.0041
T3-O2	0.1158	0.0503	9.4750	0.0039
T3-Fp1	0.1164	0.0645	4.7599	0.0354
T3-F7	0.1006	0.0652	2.6602	0.1111
T3-T3				
T3-T5	0.1084	0.0567	7.8181	0.0081
T3-O1	0.0985	0.0501	5.4716	0.0247
T3-F4	0.1277	0.0574	6.9955	0.0118
T3-C4	0.1364	0.0520	12.1654	0.0012
T3-P4	0.1183	0.0511	10.1081	0.0029
T3-F3	0.1484	0.0591	8.5707	0.0057
T3-C3	0.1938	0.0580	17.5759	0.0002

Beta	Mean WS	Mean TD	Signed F	p
T3-P3	0.1352	0.0636	6.6031	0.0142
T3-Fpz1	0.1238	0.0818	2.6155	0.1141
T3-Fz	0.1278	0.0564	6.8776	0.0125
T3-Cz	0.1603	0.0460	15.5836	0.0003
T3-Pz	0.1374	0.0491	12.6330	0.0010
T3-Oz	0.1035	0.0475	6.1994	0.0173
T5-Fp2	0.1149	0.0782	3.3885	0.0735
T5-F8	0.1145	0.0693	4.9817	0.0316
T5-T4	0.1132	0.0508	11.2749	0.0018
T5-T6	0.0914	0.0425	13.9009	0.0006
T5-O2	0.0816	0.0453	7.2838	0.0103
T5-Fp1	0.1179	0.0683	5.6222	0.0229
T5-F7	0.1095	0.0685	4.3373	0.0441
T5-T3	0.1084	0.0567	7.8181	0.0081
T5-T5				
T5-O1	0.1047	0.0491	7.8114	0.0081
T5-F4	0.1087	0.0584	8.2080	0.0068
T5-C4	0.1023	0.0465	12.2096	0.0012
T5-P4	0.0882	0.0424	9.2991	0.0042
T5-F3	0.1182	0.0640	7.4938	0.0094
T5-C3	0.1390	0.0673	13.5294	0.0007
T5-P3	0.1322	0.0724	9.0900	0.0046
T5-Fpz1	0.1282	0.0810	4.2104	0.0471
T5-Fz	0.1084	0.0600	8.9243	0.0049
T5-Cz	0.1192	0.0495	17.1136	0.0002
T5-Pz	0.1234	0.0524	15.7285	0.0003
T5-Oz	0.1004	0.0489	7.5358	0.0092
O1-Fp2	0.0971	0.0504	4.8714	0.0334
O1-F8	0.0978	0.0500	5.7574	0.0214
O1-T4	0.0938	0.0497	4.3344	0.0441
O1-T6	0.0876	0.0520	3.3124	0.0766
O1-O2	0.0616	0.0492	0.8382	0.3657
O1-Fp1	0.0976	0.0501	5.8728	0.0202
O1-F7	0.0885	0.0623	1.8011	0.1875
O1-T3	0.0985	0.0501	5.4716	0.0247
O1-T5	0.1047	0.0491	7.8114	0.0081
O1-O1				
O1-F4	0.1013	0.0542	7.0827	0.0113
O1-C4	0.0857	0.0514	5.4238	0.0253
O1-P4	0.0836	0.0420	6.0317	0.0187
O1-F3	0.0983	0.0509	10.7979	0.0022
O1-C3	0.0947	0.0495	13.7922	0.0007
O1-P3	0.0900	0.0446	8.4375	0.0061
O1-Fpz1	0.1021	0.0531	5.0555	0.0304
O1-Fz	0.1017	0.0515	7.5091	0.0093
O1-Cz	0.0840	0.0449	13.3359	0.0008
O1-Pz	0.0778	0.0409	9.2683	0.0042
O1-Oz	0.0656	0.0630	0.0436	0.8357
F4-Fp2	0.2762	0.1126	12.5714	0.0011
F4-F8	0.2163	0.0542	20.9570	0.0000
F4-T4	0.1050	0.0619	3.7550	0.0601
F4-T6	0.1191	0.0807	4.7303	0.0359
F4-O2	0.1245	0.0683	7.6354	0.0088
F4-Fp1	0.2032	0.0839	8.4207	0.0061
F4-F7	0.1787	0.0657	10.9625	0.0020
F4-T3	0.1277	0.0574	6.9955	0.0118

Beta	Mean WS	Mean TD	Signed F	p
F4-T5	0.1087	0.0584	8.2080	0.0068
F4-O1	0.1013	0.0542	7.0827	0.0113
F4-F4				
F4-C4	0.1158	0.0677	8.6698	0.0055
F4-P4	0.1124	0.0705	6.6823	0.0137
F4-F3	0.1029	0.0562	4.3139	0.0446
F4-C3	0.0976	0.0675	3.1043	0.0861
F4-P3	0.0987	0.0637	4.3577	0.0436
F4-Fpz1	0.2564	0.1199	8.0290	0.0073
F4-Fz	0.1387	0.1014	1.5242	0.2246
F4-Cz	0.1453	0.0662	10.7469	0.0022
F4-Pz	0.1215	0.0650	8.9050	0.0049
F4-Oz	0.1359	0.0680	9.1521	0.0044
C4-Fp2	0.1917	0.0713	11.1947	0.0019
C4-F8	0.1939	0.0574	19.5237	0.0001
C4-T4	0.1235	0.0523	6.7706	0.0131
C4-T6	0.1066	0.0625	9.3505	0.0041
C4-O2	0.1137	0.0607	9.6081	0.0036
C4-Fp1	0.1733	0.0668	9.8521	0.0033
C4-F7	0.1742	0.0580	14.8318	0.0004
C4-T3	0.1364	0.0520	12.1654	0.0012
C4-T5	0.1023	0.0465	12.2096	0.0012
C4-O1	0.0857	0.0514	5.4238	0.0253
C4-F4	0.1158	0.0677	8.6698	0.0055
C4-C4				
C4-P4	0.1031	0.0663	5.4534	0.0249
C4-F3	0.1125	0.0565	8.7908	0.0052
C4-C3	0.0751	0.0405	8.5563	0.0058
C4-P3	0.0846	0.0474	6.6725	0.0138
C4-Fpz1	0.1901	0.0770	8.4425	0.0061
C4-Fz	0.1333	0.0790	5.4966	0.0244
C4-Cz	0.0948	0.0434	11.8649	0.0014
C4-Pz	0.1011	0.0536	7.2765	0.0104
C4-Oz	0.1238	0.0653	7.8661	0.0079
P4-Fp2	0.1322	0.0618	8.4441	0.0061
P4-F8	0.1330	0.0620	9.4831	0.0038
P4-T4	0.1206	0.0581	6.3931	0.0157
P4-T6	0.0810	0.0481	4.9317	0.0324
P4-O2	0.1249	0.0572	14.5321	0.0005
P4-Fp1	0.1327	0.0601	9.2039	0.0043
P4-F7	0.1349	0.0544	14.1178	0.0006
P4-T3	0.1183	0.0511	10.1081	0.0029
P4-T5	0.0882	0.0424	9.2991	0.0042
P4-O1	0.0836	0.0420	6.0317	0.0187
P4-F4	0.1124	0.0705	6.6823	0.0137
P4-C4	0.1031	0.0663	5.4534	0.0249
P4-P4				
P4-F3	0.1060	0.0624	7.8715	0.0079
P4-C3	0.0931	0.0491	11.9842	0.0013
P4-P3	0.0629	0.0295	28.3072	0.0000
P4-Fpz1	0.1410	0.0632	7.9500	0.0076
P4-Fz	0.1115	0.0719	5.2518	0.0276
P4-Cz	0.1035	0.0603	9.0384	0.0047
P4-Pz	0.0875	0.0345	11.0023	0.0020
P4-Oz	0.1388	0.0561	10.4540	0.0025
F3-Fp2	0.1470	0.0642	6.1692	0.0175

Beta	Mean WS	Mean TD	Signed F	p
F3-F8	0.1122	0.0694	3.0489	0.0889
F3-T4	0.1080	0.0770	2.2596	0.1411
F3-T6	0.1331	0.0858	4.9238	0.0325
F3-O2	0.1237	0.0644	8.6040	0.0057
F3-Fp1	0.2551	0.1036	10.3923	0.0026
F3-F7	0.2364	0.0642	17.2969	0.0002
F3-T3	0.1484	0.0591	8.5707	0.0057
F3-T5	0.1182	0.0640	7.4938	0.0094
F3-O1	0.0983	0.0509	10.7979	0.0022
F3-F4	0.1029	0.0562	4.3139	0.0446
F3-C4	0.1125	0.0565	8.7908	0.0052
F3-P4	0.1060	0.0624	7.8715	0.0079
F3-F3				
F3-C3	0.1238	0.0739	5.4282	0.0252
F3-P3	0.1051	0.0731	5.4328	0.0252
F3-Fpz1	0.2095	0.0935	7.0152	0.0117
F3-Fz	0.1123	0.0576	8.7568	0.0053
F3-Cz	0.1682	0.0588	11.8988	0.0014
F3-Pz	0.1125	0.0661	7.7446	0.0083
F3-Oz	0.1235	0.0603	8.9626	0.0048
C3-Fp2	0.1400	0.0812	3.8263	0.0578
C3-F8	0.1128	0.0703	3.0621	0.0882
C3-T4	0.1024	0.0618	4.1447	0.0488
C3-T6	0.1072	0.0640	8.3045	0.0065
C3-O2	0.1053	0.0549	10.2364	0.0028
C3-Fp1	0.1830	0.0742	7.9904	0.0075
C3-F7	0.2021	0.0633	15.1486	0.0004
C3-T3	0.1938	0.0580	17.5759	0.0002
C3-T5	0.1390	0.0673	13.5294	0.0007
C3-O1	0.0947	0.0495	13.7922	0.0007
C3-F4	0.0976	0.0675	3.1043	0.0861
C3-C4	0.0751	0.0405	8.5563	0.0058
C3-P4	0.0931	0.0491	11.9842	0.0013
C3-F3	0.1238	0.0739	5.4282	0.0252
C3-C3				
C3-P3	0.1180	0.0701	6.0700	0.0184
C3-Fpz1	0.1656	0.0840	4.7439	0.0357
C3-Fz	0.1149	0.0755	4.0393	0.0516
C3-Cz	0.1222	0.0438	13.2496	0.0008
C3-Pz	0.1103	0.0559	10.4060	0.0026
C3-Oz	0.1145	0.0558	10.1479	0.0029
P3-Fp2	0.1027	0.0697	2.6963	0.1088
P3-F8	0.0966	0.0587	5.5859	0.0233
P3-T4	0.0910	0.0430	9.5331	0.0038
P3-T6	0.0775	0.0360	16.3647	0.0002
P3-O2	0.0937	0.0381	13.0965	0.0009
P3-Fp1	0.1092	0.0654	3.6564	0.0634
P3-F7	0.1079	0.0618	5.2920	0.0270
P3-T3	0.1352	0.0636	6.6031	0.0142
P3-T5	0.1322	0.0724	9.0900	0.0046
P3-O1	0.0900	0.0446	8.4375	0.0061
P3-F4	0.0987	0.0637	4.3577	0.0436
P3-C4	0.0846	0.0474	6.6725	0.0138
P3-P4	0.0629	0.0295	28.3072	0.0000
P3-F3	0.1051	0.0731	5.4328	0.0252
P3-C3	0.1180	0.0701	6.0700	0.0184

Beta	Mean WS	Mean TD	Signed F	p
P3-P3				
P3-Fpz1	0.1125	0.0727	2.5237	0.1204
P3-Fz	0.1010	0.0701	4.0482	0.0513
P3-Cz	0.1229	0.0611	8.9636	0.0048
P3-Pz	0.1102	0.0339	14.1521	0.0006
P3-Oz	0.1198	0.0418	11.7332	0.0015
Fpz1-Fp2	0.1250	0.0997	0.7945	0.3783
Fpz1-F8	0.1590	0.1381	0.5035	0.4823
Fpz1-T4	0.1457	0.1047	2.8874	0.0975
Fpz1-T6	0.1728	0.0899	7.6565	0.0087
Fpz1-O2	0.1403	0.0699	6.3748	0.0159
Fpz1-Fp1	0.0817	0.0599	1.3627	0.2503
Fpz1-F7	0.1106	0.0846	1.4407	0.2374
Fpz1-T3	0.1238	0.0818	2.6155	0.1141
Fpz1-T5	0.1282	0.0810	4.2104	0.0471
Fpz1-O1	0.1021	0.0531	5.0555	0.0304
Fpz1-F4	0.2564	0.1199	8.0290	0.0073
Fpz1-C4	0.1901	0.0770	8.4425	0.0061
Fpz1-P4	0.1410	0.0632	7.9500	0.0076
Fpz1-F3	0.2095	0.0935	7.0152	0.0117
Fpz1-C3	0.1656	0.0840	4.7439	0.0357
Fpz1-P3	0.1125	0.0727	2.5237	0.1204
Fpz1-Fpz1				
Fpz1-Fz	0.2475	0.0870	11.2198	0.0018
Fpz1-Cz	0.1960	0.0810	7.6076	0.0089
Fpz1-Pz	0.1362	0.0685	5.0914	0.0299
Fpz1-Oz	0.1262	0.0559	6.3060	0.0164
Fz-Fp2	0.1909	0.0670	10.5229	0.0025
Fz-F8	0.1288	0.0765	3.4530	0.0709
Fz-T4	0.1080	0.0763	2.5785	0.1166
Fz-T6	0.1284	0.0850	4.5426	0.0396
Fz-O2	0.1283	0.0646	9.0252	0.0047
Fz-Fp1	0.2217	0.0632	12.7791	0.0010
Fz-F7	0.1804	0.0556	11.9220	0.0014
Fz-T3	0.1278	0.0564	6.8776	0.0125
Fz-T5	0.1084	0.0600	8.9243	0.0049
Fz-O1	0.1017	0.0515	7.5091	0.0093
Fz-F4	0.1387	0.1014	1.5242	0.2246
Fz-C4	0.1333	0.0790	5.4966	0.0244
Fz-P4	0.1115	0.0719	5.2518	0.0276
Fz-F3	0.1123	0.0576	8.7568	0.0053
Fz-C3	0.1149	0.0755	4.0393	0.0516
Fz-P3	0.1010	0.0701	4.0482	0.0513
Fz-Fpz1	0.2475	0.0870	11.2198	0.0018
Fz-Fz				
Fz-Cz	0.2092	0.0756	16.1478	0.0003
Fz-Pz	0.1203	0.0699	7.1753	0.0109
Fz-Oz	0.1326	0.0642	8.1749	0.0069
Cz-Fp2	0.1857	0.0771	8.5170	0.0059
Cz-F8	0.1741	0.0630	14.4806	0.0005
Cz-T4	0.1036	0.0504	4.1327	0.0491
Cz-T6	0.0945	0.0555	8.7924	0.0052
Cz-O2	0.0938	0.0503	10.6454	0.0023
Cz-Fp1	0.1967	0.0713	9.6177	0.0036
Cz-F7	0.1997	0.0586	14.8973	0.0004
Cz-T3	0.1603	0.0460	15.5836	0.0003

Beta	Mean WS	Mean TD	Signed F	p
Cz-T5	0.1192	0.0495	17.1136	0.0002
Cz-O1	0.0840	0.0449	13.3359	0.0008
Cz-F4	0.1453	0.0662	10.7469	0.0022
Cz-C4	0.0948	0.0434	11.8649	0.0014
Cz-P4	0.1035	0.0603	9.0384	0.0047
Cz-F3	0.1682	0.0588	11.8988	0.0014
Cz-C3	0.1222	0.0438	13.2496	0.0008
Cz-P3	0.1229	0.0611	8.9636	0.0048
Cz-Fpz1	0.1960	0.0810	7.6076	0.0089
Cz-Fz	0.2092	0.0756	16.1478	0.0003
Cz-Cz				
Cz-Pz	0.1177	0.0713	4.9525	0.0321
Cz-Oz	0.1088	0.0544	11.7071	0.0015
Pz-Fp2	0.1287	0.0639	5.8916	0.0201
Pz-F8	0.1265	0.0543	10.2326	0.0028
Pz-T4	0.1085	0.0407	8.3715	0.0063
Pz-T6	0.0841	0.0395	11.2037	0.0018
Pz-O2	0.0894	0.0421	12.7508	0.0010
Pz-Fp1	0.1362	0.0630	7.0738	0.0114
Pz-F7	0.1398	0.0534	12.5752	0.0011
Pz-T3	0.1374	0.0491	12.6330	0.0010
Pz-T5	0.1234	0.0524	15.7285	0.0003
Pz-O1	0.0778	0.0409	9.2683	0.0042
Pz-F4	0.1215	0.0650	8.9050	0.0049
Pz-C4	0.1011	0.0536	7.2765	0.0104
Pz-P4	0.0875	0.0345	11.0023	0.0020
Pz-F3	0.1125	0.0661	7.7446	0.0083
Pz-C3	0.1103	0.0559	10.4060	0.0026
Pz-P3	0.1102	0.0339	14.1521	0.0006
Pz-Fpz1	0.1362	0.0685	5.0914	0.0299
Pz-Fz	0.1203	0.0699	7.1753	0.0109
Pz-Cz	0.1177	0.0713	4.9525	0.0321
Pz-Pz				
Pz-Oz	0.1104	0.0466	11.8989	0.0014
Oz-Fp2	0.1298	0.0597	6.3628	0.0160
Oz-F8	0.1386	0.0705	6.0932	0.0182
Oz-T4	0.1359	0.0739	4.6306	0.0378
Oz-T6	0.1350	0.0679	6.0384	0.0187
Oz-O2	0.0753	0.0552	1.8979	0.1764
Oz-Fp1	0.1201	0.0515	6.8092	0.0129
Oz-F7	0.1112	0.0725	1.4232	0.2403
Oz-T3	0.1035	0.0475	6.1994	0.0173
Oz-T5	0.1004	0.0489	7.5358	0.0092
Oz-O1	0.0656	0.0630	0.0436	0.8357
Oz-F4	0.1359	0.0680	9.1521	0.0044
Oz-C4	0.1238	0.0653	7.8661	0.0079
Oz-P4	0.1388	0.0561	10.4540	0.0025
Oz-F3	0.1235	0.0603	8.9626	0.0048
Oz-C3	0.1145	0.0558	10.1479	0.0029
Oz-P3	0.1198	0.0418	11.7332	0.0015
Oz-Fpz1	0.1262	0.0559	6.3060	0.0164
Oz-Fz	0.1326	0.0642	8.1749	0.0069
Oz-Cz	0.1088	0.0544	11.7071	0.0015
Oz-Pz	0.1104	0.0466	11.8989	0.0014
Oz-Oz				

Supplementary Table S21. Demographic and descriptive data of our subjects and night sleep records, repectively.

Subjects	WS				TD			
	Age (years)	Gender (M/F)	NREM (min)	REM (min)	Age (years)	Gender (M/F)	NREM (min)	REM (min)
Nr.1	6	M	422.67	105.67	6	M	392.00	117.67
Nr.2	6	M	362.00	116.00	6	M	391.00	138.67
Nr.3	11	M	361.00	140.33	11	M	387.67	111.67
Nr.4	13	F	363.00	118.33	12	F	381.33	146.67
Nr.5	15	F	428.67	112.00	15	F	367.00	87.00
Nr.6	16	F	429.67	61.00	16	F	359.33	101.33
Nr.7	16	F	300.00	142.00	17	F	303.00	184.33
Nr.8	17	F	330.33	91.67	18	F	279.33	105.67
Nr.9	18	M	316.67	95.33	19	F	364.67	94.67
Nr.10	20	F	267.33	91.00	19	F	371.00	108.33
Nr.11	20	F	345.67	107.67	21	F	305.67	103.33
Nr.12	22	F	343.33	89.00	23	M	330.33	102.33
Nr.13	24	M	391.67	111.33	23	F	397.00	126.33
Nr.14	25	F	356.33	107.33	24	F	299.00	146.67
Nr.15	26	F	304.00	105.33	25	F	281.67	61.00
Nr.16	26	F	317.67	65.33	25	F	356.67	147.00
Nr.17	27	F	418.33	56.00	27	F	325.00	118.33
Nr.18	27	M	367.00	61.33	28	M	374.67	129.33
Nr.19	28	M	218.00	51.67	28	M	385.67	133.00
Nr.20	29	F	377.33	121.33	29	F	338.00	148.67
